# Comprehensive metabolomic profiling of Egyptian *Salvia* species reveals promising leads for chemopreventive and anti-inflammatory drug development: *In vitro* and *In silico* study

**DOI:** 10.1038/s41598-026-55968-x

**Published:** 2026-06-09

**Authors:** Ahmed R. Hamed, Sherin K. Ali, Sally A. Abdel-Halim, Shaymaa M. Bata, Nesrine M. Hegazi, Tarik A. Mohamed, Mohamed-Elamir F. Hegazy

**Affiliations:** 1 Chemistry of Medicinal Plants Department, National Research Centre, 33 El-Bohouth St., Dokki, 12622 Giza Egypt; 2 Phytochemistry and Plant Systematics Department, National Research Centre, 33 El- Bohouth St., Dokki, 12622 Giza Egypt

**Keywords:** Inducible nitric oxide synthase (iNOS), *Salvia*, Molecular networking, NAD(P)H: Quinone oxidoreductase 1, Network pharmacology, Molecular dynamics, Biochemistry, Cancer, Computational biology and bioinformatics, Drug discovery

## Abstract

The vast majority of human malignancies are the result of sustained inflammatory responses. Nowadays, it is well-known that there is a substantial association between inflammation and cancer. The *Salvia* genus has garnered attention due to its richness of phytochemical profiles with anti-inflammatory and chemopreventive properties. Although the recognized therapeutic potential of *Salvia* species is recognized, there is considerable variation in their reported chemical composition, and less is known about their compositional differences. In this work, UPLC-HRMS/MS coupled with molecular networking was employed to glean a holistic overview of the chemodiversity of the metabolome of three *Salvia* species. This was followed by molecular docking of 218 compounds against KEAP1 (PDB: 4IQK) and iNOS (PDB: 1M8D), validated by positive controls. Network pharmacology, Protein–Protein Interaction (PPI), and enrichment analyses identified hub genes (TNF, CASP3, CASP8, PARP1, iNOS, EGFR, PPARG, AKR1C isoforms, and NQO1) linked to inflammatory and antioxidant signaling. Docking analysis predicted that several major compounds may exhibit dual binding affinities, supported by chemoinformatics, ADME, and toxicity predictions. Cirsimaritin was evaluated against AKR1C3 and PARP1 using molecular docking and 100-ns molecular dynamics simulations, representing different stability and interaction profiles between the two targets. The results indicate a dual binding activity, with Cirsimaritin exhibiting primary and stable interaction with AKR1C3 alongside a secondary, dynamic modulatory interaction with PARP1. Mostly bioactive compounds were predominantly found in *Salvia multicaulis*. The extracts were screened for their *in vitro* anti-inflammatory and chemoprevention activities. Preliminary experiments confirmed that *S. multicaulis* acts as an inducer of chemopreventive NAD(P)H: Quinone oxidoreductase 1 (NQO1) protein expression in a murine hepatoma (Hepa1c1c7) chemoprevention model. It also demonstrated the ability to inhibit the lipopolysaccharides (LPS)-induced nitric oxide (NO) production and iNOS protein expression in the RAW264.7 macrophage model. Furthermore, flavonoids and terpenoids were identified to be the major components in this extract, which might be responsible for anti-inflammatory and chemoprevention activities. Our results support the possible application of *S. multicaulis* in the treatment of human malignancies.

**Supplementary Information:**

The online version contains supplementary material available at 10.1038/s41598-026-55968-x.

## Introduction

The *Salvia* genus, belonging to the Lamiaceae family, comprises more than 900 species distributed worldwide. In recent years, there has been increasing global interest in *Salvia* L. species due to their applications in folk medicine, cosmetics, and pharmaceutical industries^1^. Additionally, some species are cultivated as ornamental plants^2^. In Egypt, nine *Salvia* species have been recorded, including *S. aegyptiaca*,* S. multicaulis*,* and S. lanigera*^3^. Previous phytochemical studies of leaves and fruits of various *Salvia* species have revealed a rich diversity of phenolic acids, flavonoids, and terpenoids^4^. Owing to the diversity of these phytochemical constituents, *Salvia* species exhibit a wide array of biological activities, including antioxidant, anti-inflammatory, cytotoxic, acetylcholinesterase-inhibitory, and antimicrobial effects^5^.

In some countries, cancer has exceeded cardiovascular disorders as the leading cause of mortality, particularly due to increasing urbanization and environmental changes^2^. Chronic inflammation is a well-recognized factor in the initiation and progression of various human malignancies. Persistent inflammatory responses can lead to DNA damage, promote uncontrolled cell proliferation, and inhibit programmed cell death, thereby creating a microenvironment conducive to tumor initiation and growth^6^. Currently, numerous studies have investigated the chemopreventive properties of plant-derived natural compounds, both* in vitro* and *in vivo*.

Among the effective strategies for cancer prevention, chemoprevention has gained considerable attention, as it aims to block, reverse, or delay the multistep process of carcinogenesis. This can be achieved, in part, through the activation of phase II detoxifying and antioxidant enzymes, such as NAD(P)H: quinone oxidoreductase 1 (NQO1) and heme oxygenase-1 (HO-1), which play crucial roles in cellular defense mechanisms against oxidative stress and xenobiotic-induced damage^6^. Notably, anti-inflammatory agents are often effective in preventing the elevation of nitric oxide (NO) levels^7^.

Metabolomics approaches have become essential for the comprehensive profiling of complex plant matrices, providing detailed insights into their chemical composition and bioactive potential. In this context, UPLC-HRMS/MS (Ultra-performance liquid chromatography coupled to high-resolution tandem mass spectrometry) was employed in both ionization modes and coupled to feature-based molecular networking (FBMN) *via* the GNPS platform (Global Natural Products Social Molecular Networking).

By integrating these approaches, metabolomics facilitates the systematic characterization of characteristic secondary metabolites, offering valuable insights into the chemotaxonomic relationships and potential biological activities of *Salvia* species.

Network pharmacology (NP) integrates multiple disciplines and methodologies for identifying potential clinical drug targets. Molecular docking is a key technique for predicting target-drug interactions^8^. Molecular dynamics (MD) simulations are widely acknowledged as potent computational methods for investigating protein–ligand interactions, enabling the assessment of conformational stability and solvent effects in conditions that resemble physiological states^9^. These simulations offer atomistic insights into structure dynamics, functioning as computational filters that guide hit optimization and drug formulation^10^. The combination of these approaches provides a more precise framework for drug discovery and the repurposing of effective therapeutics^8^.

In this study, selected *Salvia* metabolites were examined through *in silico* ligand-docking experiments against iNOS (1M8D) as a catalytic hub for nitric oxide production and Keap1 (4IQK) as a therapeutic target molecule for NQO1^11–13^. Network pharmacology (NP) was further employed to identify disease-related networks, pathways, and target genes. The tentatively identified compounds were screened for their potential anti-inflammatory and chemopreventive activities within a network pharmacology framework.

Initially, major compounds and their intersecting genes associated with anti-inflammatory and chemopreventive activities were compiled and analyzed separately.

Next, a protein–protein interaction (PPI) network was constructed, and key hub genes were identified. Gene Ontology (GO) and Kyoto Encyclopedia of Genes and Genomes (KEGG) enrichment analyses were also performed. Molecular docking experiments provided additional validation of the potential bioactive compounds and their primary targets. Finally, MD simulations were employed to validate and enhance the stability of the chosen protein–ligand complexes.

In parallel, the efficiency of the selected *Salvia* species as inducers of the chemopreventive marker NQO1 and their anti-inflammatory potency to attenuate the LPS-induced nitric oxide and inducible nitric oxide synthase (iNOS) protein expression were evaluated in mouse models of chemoprevention and inflammation, respectively.

Owing to the economic and industrial importance of Genus *Salvia*, this study aimed to conduct an integrative metabolomic and pharmacological investigation of Egyptian *Salvia* species, linking their chemical composition to potential anti-inflammatory and chemopreventive mechanisms, and providing novel insights into their therapeutic potential.

## Materials and methods

### Plant materials

The flowering aerial parts of three *Salvia* species (*S. aegyptiaca*, *S. multicaulis*, and *S. lanigera*) were collected in April 2020 from the same natural habitat in South Sinai, Egypt. The three plants were authenticated by Dr. Mona M. Marzouk, professor of phytochemistry and plant systematics at the National Research Centre. Voucher specimens [voucher No. SA-322 (*S. aegyptiaca*), SL-305 (*S. lanigera*), and SM-310 (*S. multicaulis*)] were deposited in the herbarium of the National Research Centre (CAIRC), Cairo, Egypt.

### Phytochemical investigation

#### Chemicals

All chemicals were purchased from Sigma-Aldrich (St. Louis, MO, USA). Milli-Q water was supplied by a Millipore MR3 purifier system and was used for UPLC analysis.

For biological experiments, lipopolysaccharide (LPS) was purchased from Sigma Chemical. The inducible nitric oxide synthase (iNOS) antibody was obtained from Biomatik (Canada), whereas the NAD(P)H: quinone dehydrogenase 1 (NQO1) antibody was purchased from Elabscience (Texas, USA).

#### Preparation of the Extracts

The air-dried, powdered aerial parts of the studied *Salvia* species (100 g each) were first extracted by maceration in 1 L of CH₂Cl₂/MeOH (1:1, v/v) at room temperature for 24 h. The filtrates were evaporated under reduced pressure to obtain the CH₂Cl₂/MeOH extracts. The plant residues were subsequently subjected to a second extraction with 1 L of MeOH/H₂O (70:30, v/v) under the same conditions. The resulting filtrates were evaporated under reduced pressure, lyophilized, and stored at − 20 °C until further analysis. Each lyophilized extract was re-dissolved in HPLC-grade MeOH to a final concentration of 2 µg/mL.

#### UPLC-HRMS/MS analysis

Dried CH₂Cl₂/MeOH (1:1) and MeOH/H₂O (70:30) extracts of each *Salvia* species were re-dissolved in HPLC-grade MeOH to a final concentration of 2 mg/mL and diluted 1:1000 prior to analysis. Samples were analyzed using a 1260 Infinity II high-performance liquid chromatography (HPLC) system (Agilent Technologies, Waldbronn, Germany) coupled to a 6545 QTOF mass spectrometer (Agilent Technologies, Waldbronn, Germany) equipped with an Agilent Jet Stream electrospray ionization (ESI) source.

Chromatographic separation was achieved on an EclipsePlus C18 RRHD column (50 mm × 2.1 mm, 1.8 μm; Agilent Technologies, Waldbronn, Germany). The mobile phase consisted of 2% acetonitrile in H₂O (A) and MeOH (B) with the following gradient: 15% B for 1 min, increased linearly to 95% B over 24 min, held at 95% B for 5 min, returned to 15% B in 1 min, and re-equilibrated at 15% B for 9 min. The flow rate was maintained at 0.2 mL/min, and the injection volume was 5 µL.

Mass spectrometric analyses were performed in both positive- and negative-ion automated data-dependent acquisition (DDA) modes. Full MS scans were acquired over an m/z range of 50–1500 Da at a scan rate of 1 spectrum/s, followed by MS/MS fragmentation of the two most intense precursor ions. Dynamic exclusion was applied after five spectra for 0.5 min.

The ESI source parameters were as follows: capillary voltage 3.5 kV, nozzle voltage 1 kV, fragmentor voltage 175 (arbitrary units), drying gas temperature 320 °C, sheath gas temperature 350 °C, drying gas flow rate 10 L/min, and nebulizer pressure 35 psig. High-purity nitrogen was used as the nebulizing, auxiliary, and collision gas. Data were acquired in centroid mode, and the instrument was calibrated before each batch of samples. The collision energy was fixed at 35 V^14^.

#### Data preprocessing, molecular networking, and compound dereplication

The feature-based molecular network (FBMN) was built from each species’ HPLCHRMS/MS data in both ionization modes. Firstly, the MS Convert program was used to convert raw data files into 32-bit MzXML files, which were then loaded into Mzmine 2.5.3 for feature identification. The produced MGF files and the feature quantification tables (CSV file) were used for the construction of two feature-based molecular networks (FBMNs) following the online workflow in the GNPS platform (http://gnps.ucsd.edu)^15^. The parameters applied for the construction of the FBMNs via the GNPS platform are listed in (Supplementary Table S1). Cytoscape version 3.9.1 (https://cytoscape.org/) was used for the network visualization.

The negative FBMN is available at:

https://gnps.ucsd.edu/ProteoSAFe/status.jsp?task=39c124dd7e604607bc37a83e3c7ad81e.

The positive FBMN is available at:

https://gnps.ucsd.edu/ProteoSAFe/status.jsp?task=67297950642242adb5853a73396aec5d.

The metabolites’ dereplication was based on the chromatographic behavior, molecular composition, and fragmentation sequence synchronized with the *in silico* fragmentation trees proposed by SIRIUS CSI: Finger ID 5.6.3^16^ and searching the online structure databases (i.e., Lotus, COCONUT^17^, Pubchem, etc.,) with 10 ppm *m/z* tolerance (Supplementary Table S1), with reference to the LOTUS online database for compounds previously isolated from the *Salvia* genus^18^.

#### Molecular docking

Compound selection for molecular docking was performed using a multi-criteria strategy to ensure both biological and analytical relevance. A total of 218 compounds tentatively identified by UPLC-HRMS/MS were initially subjected to molecular docking against iNOS and KEAP1. The compounds were ranked according to their predicted binding affinities toward both targets. To further refine the selection, compound prevalence in the LC–MS/MS chromatograms was considered. In addition, only compounds with previously reported structural identification in the literature, including confirmation by NMR spectroscopy, were retained. Based on this integrated selection approach, ten compounds were ultimately selected for detailed molecular docking analysis. These small-molecule ligands, 218 identified metabolites, were generated and optimized by Chem 3D software. The PDB file for the target proteins, KEAP1 complex crystal structures (PDB ID: 4IQK), as a key player for antioxidant pathways, and the crystal structure of iNOS (PDB ID: 1M8D) as a structural and catalytic hub for nitric oxide production, integrating cofactors to produce inflammatory responses, were downloaded from the Protein Data Bank (http://www.rcsb.org/pdb/). These targets were docked with the positive controls: 4’-bromoflavone, a potent modulator of KEAP1/NRF2/ARE pathway leading to NQO1 induction, and N-(3-(aminomethyl)benzyl)acetamidine for iNOS to verify the docking method. PYMOL software was used to remove water molecules and small molecular ligands with three-dimensional structures of protein receptors^19^. The protein receptor was hydrogenated by Auto Dock Tools. Docking grid box parameters were created to encompass the active sites of the selected proteins. The docking grid was centered at coordinates X = 125.892, Y = 104.521, and Z = 84.496 in the case of inducible nitric oxide synthase (iNOS; PDB ID: 1M8D), with grid dimensions of 100 × 112 × 86 Å³ to assure complete coverage of the catalytic binding pocket. While the grid box for Kelch-like ECH-associated protein 1 (KEAP1; PDB ID: 4IQK) was established with center coordinates of X = − 43.812, Y = 2.963, and Z = − 13.919, and grid dimensions of 52 × 68 × 56 Å³. Finally, the molecular docking was performed under Vina. The docking results were then analyzed for interaction patterns using PyMOL 3.1.4.1. and The representation and graphical analyses were performed using the BIOVIA Discovery Studio Visualizer software^20^. To validate the reliability of the molecular docking workflow, a validation step was performed for all target proteins, including KEAP1 and iNOS. Co-crystallized ligands from the original protein structures were extracted and docked back into their respective binding sites using the same docking parameters applied to the test compounds^21^. The resulting poses were compared to the native ligand conformations using root mean square deviation (RMSD) analysis. An RMSD value below 2.0 Å was considered acceptable, confirming the accuracy of the docking protocol. Moreover, Docking was performed three times independently to calculate mean values and standard deviations of the lowest binding energies.

#### Chemoinformatics, drug likeness, and ADME prediction

The chemoinformatic data and drug likeness of major tentatively identified compounds present in *Salvia* species using UPLC-HRMS/MS were assessed using the Swiss ADME server (http://www.swissadme.ch), an online tool specifically designed for calculating pharmacokinetic properties, oral bioavailability, and drug-likeness^22^. To evaluate the drug-likeness of these compounds, Lipinski’s Rule of Five (RO5) was applied, which serves as a screening criterion for potential oral drugs in humans.

The parameters taken into consideration included molecular weight (MW), topological polar surface area (TPSA), the number of rotatable bonds, hydrogen bond acceptor (HBA), and hydrogen bond donor (HBD) numbers, as well as water solubility.

#### Target proteins prediction

The identification of targets for *Salvia* species metabolites was carried out using the Swiss Target Prediction databases (http://www.swisstargetprediction.ch/)^23^ and Superpred (https://prediction.charite.de/)^24^. To achieve this, the canonical SMILES representation of each compound was entered into the Swiss Target Prediction database and Superpred. Subsequently, candidate targets with high probability scores were selected and then further standardized using the UniProt database (http://www.uniprot.org/).

#### Compounds toxicity assessment

Drug toxicity refers to the harmful effects of a substance when taken in excessive amounts or when the body is unable to metabolize and eliminate it properly. It can range from mild side effects to severe, life-threatening reactions. Protox II server software was utilized (https://tox-new.charite.de/protox_II) for the prediction of various toxicity indicators, including carcinogenicity, Immunotoxicity, Irritating effect, reproductive, hepatotoxicity, and mutagenicity.

The studied compounds were also subjected to assessment for predicting their LD_50_ values and drug toxicity classifications. LD_50_ values are commonly expressed in mg/kg of body weight and represent the dose at which 50% of test subjects succumb after exposure to a substance. Toxicity classes are defined in accordance with the Global Harmonization System (GHS) for the categorization and labeling of substances^25^.

#### Potential targets associated with KEAP1 and iNOS

KEAP1-related chemoprevention targets, and NOS2 (iNOS)-related anti-inflammatory targets were retrieved from GeneCard (http://www.genecards.org). The common names of the targets were also standardized using UniProtKB (https://www.uniprot.org). Using " KEAP1-NRF2 related chemoprevention " and “NOS2 -related anti-inflammatory”, separately as keywords, potential targets for them were searched in the GeneCards database. Disease-associated targets were retrieved from the GeneCards database, and only genes with a GeneCards relevance score ≥ 10 were selected for further analysis. With p-value < 0.05and |log2FC|> 1 as screening conditions, differentially expressed genes (DEGs) were obtained by differentially analyzing the gene expression profiles linked to each disease separately that were in the Gene Expression Omnibus (GEO) database. Data was visualized by a volcano plot.

The Venn diagram was utilized to select the intersection between the selected targets in the GeneCards database and DEGs in GEO databases. GEO2R was employed as a standardized and reproducible platform for differential gene expression analysis, enabling independent verification of the results. Targets that appeared at least twice in these selected targets were identified as the primary relevant targets of each disease^26^. Additionally, the Venn diagram was used to identify common targets between the bioactive components of *Salvia* species and the primary relevant targets of each disease, with the intersecting portion being considered as potential therapeutic targets for *Salvia* species intervention in each disease, separately.

#### PPI network construction and hub gene identification

Protein-protein interactions (PPI) play a pivotal role in biological processes and are essential for a comprehensive understanding of the intricate workings of a living cell. In this study, the PPI network for the identified drug targets was constructed using the STRING database (https://string-db.org/)^27^, with a specific focus on the species ‘Homo sapiens’ To ensure the reliability of the information, a confidence score threshold of > 0.7 was set. Subsequently, the results obtained from pinpointing highly interconnected regions within the PPI network were imported to visualize with Cytoscape software version 3.9.1 (https://cytoscape.org/). Hub genes were identified based on degree centrality. Nodes ranking within the top 10% of degree values were defined as hub genes and selected for subsequent analysis.

#### GO and KEGG pathway enrichment analysis

These targets were subjected to GO and KEGG analysis using the DAVID database^28^. Three GO characteristics were analyzed: biological process (BP), cellular component (CC), and molecular function (MF). Statistical significance was assessed using P-values, with a threshold of *P* < 0.05 considered significant. To control for multiple testing and reduce false positives, the false discovery rate (FDR) was calculated using the Benjamini–Hochberg method, and terms with FDR < 0.05 were retained for interpretation.

The results were effectively visualized using bar charts. GO serves as a comprehensive resource for functional genomics, offering detailed definitions of gene functions, including molecular functions. On the other hand, KEGG comprises graphical diagrams of biochemical pathways and potential signaling pathways. Pathway enrichment analysis was performed using KEGG annotations^29,30^.

#### Molecular docking analysis between compounds and hub genes

To gain a deeper understanding of the relationship, mode of interactions, and action mechanisms between the candidate proteins (or hub targets) and the major compounds that were tentatively identified from *Salvia* species, molecular docking was employed. KEAP1 and iNOS were selected as primary molecular docking targets based on their established roles in oxidative stress and inflammatory signaling. In addition, network pharmacology and protein–protein interaction (PPI) analyses identified whereas EGFR (PDB ID: 1M17), PPARG (PDB ID: 2HFP), AKR1C1 (PDB ID: 3C3U), SRC (PDB ID: 1A09), AKR1C3 (PDB ID: 1RY0), TNF (PDB ID: 7KBA), CASP3 (PDB ID: 1RHU), CASP8 (PDB ID: 1F9E), and PARP1 (PDB ID: 2RCW), as hub genes with high degree centrality and pathway relevance. Based on their central roles in the constructed network, these targets were included for further molecular docking analysis to explore potential multi-target interactions of the identified compounds. All these target proteins were downloaded from the Protein Data Bank (http://www.rcsb.org/pdb/). PYMOL software was used to remove water molecules and small molecular ligands with three-dimensional structures of protein receptors^19^. The protein receptor was hydrogenated by Auto Dock Tools. Docking grid box parameters were created to encompass the active sites of the selected proteins. Docking grid box parameters—including center coordinates (x, y, z) and grid dimensions—were explicitly reported for all nine proteins to ensure reproducibility. These values are summarized in Supplementary Table S4. Finally, the molecular docking was performed using AutoDock Vina. The docking results were then analyzed for interaction patterns using PyMOL 3.1.4.1. and the representation and graphical analyses were performed using the BIOVIA Discovery Studio Visualizer software^20^. Validation using co-crystallized reference ligands confirmed the docking protocol’s reliability, with RMSD values below 2.0 Å indicating accurate pose prediction^21^. Docking was performed three times independently to calculate mean values and standard deviations of the lowest binding energies.

#### Molecular dynamics simulation

MD simulations enable a better understanding of the ligand through a number of statistical characteristics, which are used to analyze the stability of the ligand in the protein binding site^31,32]^. Selected top-scoring complexes (AKR1C3-cirsimaritin complex and PARP1-cirsimaritin complex) from each docking method (KEAP1–NRF2 chemopreventive and iNOS-related inflammatory) were subjected to molecular dynamics (MD) simulations. The best docked pose of cirsimaritin, along with both the AKR1C3 complex and PARP1 complex were chosen as starting coordinates for a 100 ns MD simulation using a GROMACS-2023 software package (GNU, General Public License^33^ and CHARMM36 force field^34,35^ and “Swiss Param”^36^ for both protein and ligand topology preparation. The complexes were solvated within a cubic box of dimensions (100 × 100 × 100Å) using a transferable intermolecular potential with a three-point (TIP3P) water model, thus allowing a margin of 10Å minimum between the protein and each side of the 3D box. System neutralization was achieved by replacing solvent molecules with Na⁺ and Cl⁻ ions using the GROMACS genion tool, as described in the GROMACS documentation^37^. The MD simulation was performed in three stages (minimization, equilibration, and production). The minimization and equilibration stages were conducted using a 1000 kJ mol⁻¹ nm⁻² force constant to constrain all heavy atoms and maintain the original protein folding^37^. The minimization step comprised the initial optimization of each system’s shape using the steepest descent algorithm over 5,000 iterations (5,000 steps). The equilibration step involved system equilibration for 125 ps under a constant number of particles, volume, and temperature ensemble (NVT ensemble), followed by 125 ps under a constant pressure ensemble (NPT ensemble), both of which were guided by the V-rescale temperature coupling method and the Parrinello–Rahman barostat^38,39^. Finally, the production stage was run for 100 ns under NPT ensemble, in which the isothermal and isobarometric conditions were maintained at 300 K temperature and 1 bar pressure using the Parrinello Rahman barostat. The Particle Mesh Ewald’s method was used to calculate the long-range electrostatic interactions^40^. The time step of the simulated system was set to 2.0 fs. The trajectories generated from the simulation were stored every 100 ps. All position restraints were removed during the production run. To ensure equilibrium, the first 20 ns of the production run were discarded, and all analyses were performed on the equilibrated trajectory from 20 to 100 ns^41^. The resulting trajectories were analyzed concerning various structural and dynamic properties, including the RMSD, RMSF, Rg, SASA, and hydrogen bond counts. Thereafter, the systems were appraised appropriately using the respective built-in functions from GROMACS: “gmx rms”, “gmx rmsf”, “gmx gyrate”, “gmx sasa”, and “gmx hbond”. After simulation, all results were extracted and further analysed; special interest was given to the RMSD and RMSF in the MD simulations of protein-ligand interactions, solvent accessible surface area (SASA), radius of gyration, and number of hydrogen bonds. Trajectory analysis was performed using GROMACS utilities^37^. Distances are reported in Å unless otherwise specified; center-of-mass (COM) distances and solvent-accessible surface area (SASA) are reported in nm and nm², respectively.

#### MM-GBSA

The MM-GBSA method was used to estimate binding free energies and analyze the enthalpy-associated energy contributions to ligand–protein binding, the gmx_MMPBSA technique was used to the generalized-born-surface area (GB-SA) continuum solvent model^42^. The formula was used to determine the energy contributions (in kilocalories per mole) from molecular mechanics, polar solvation, and a non-polar solvation factor.

$$\Delta {{\rm{G}}_{{\rm{bind}}}} = {\rm{ }}{{\rm{G}}_{{\rm{complex}}}} - {\rm{ }}{{\rm{G}}_{{\rm{protein}}}} - {\rm{ }}{{\rm{G}}_{{\rm{ligand}}}}$$

ΔG_bind_ = calculated binding free energy of the complex, G_complex_ = binding free energy of the minimized complex, G_protein_ = binding free energy of receptor, G_ligand_ = binding free energy of unbound ligand.

### Biological investigation

#### Cell culture

Hepa-1c1c7 cells (cat# CRL-2026, ATCC^®^) were maintained as a monolayer culture in α-modified minimum essential medium Eagle supplemented with 10% (v/v) heat and charcoal-inactivated fetal bovine serum, 2 mM L-glutamine, 100 U/ml penicillin, and 100 µg/mL streptomycin. Murine macrophage RAW 264.7 cells (cat# TIP 71, ATCC^®^) were maintained in complete Dulbecco’s modified Eagle’s medium supplemented with 10% fetal bovine serum, penicillin (100 U/ml), streptomycin sulfate (100 µg/mL), and 2 mM L-glutamine.

Both cells were maintained and incubated in a humidified 5% CO_2_ incubator. Routine subculture of Hepa-1c1c7 and RAW264.7 cells was accomplished by either trypsinization with Trypsine/EDTA or scraping using sterile rubber scrapers, respectively^6^.

#### Cancer chemopreventive

##### Assessment of induction of NAD(P)H-quinone oxidoreductase 1 in Hepa-1C1C7 cells

Hepa-1c1c7 cells were seeded in 6-well plates and incubated overnight. Monolayers were treated with either vehicle [0.1% dimethyl sulfoxide (DMSO, v/v) final concentration] in culture medium or extracts in a final concentration of 100 µg/mL. Routine microscopical examination ensured that all used concentrations are non-toxic to cells.

Cells were incubated for 48 h, after which monolayers were washed with Dulbecco’s phosphate-buffered saline (DPBS) and scraped in homogenization buffer. Cell suspensions were sonicated on ice for 5 s and centrifuged at maximum speed for 10 min. Supernatants were used for NQO1 protein expression by Western blotting^43^.

#### Anti-inflammatory activity

##### Inhibition of LPS-induced NO production in RAW264.7

Seeding 96-well microwell plates with RAW264.7 cell stock (1 × 10^5^ cells/ml) and treating them were performed as described earlier^44^. Overnight incubated RAW264.7 cell cultures were treated with DMSO at a final concentration of 0.1% v/v in fresh complete media. Wells in the inflammation-inducing group (LPS^+^) were treated with 100 ng/mL of LPS. Routine microscopical examination ensured that all used concentrations are non-toxic to cells. A single non-toxic final concentration of 100 µg/mL extracts (DMSO-dissolved) was used for preliminary screening in the presence of LPS.

After incubation for 24 h, nitric oxide (NO) levels in each well were measured using the Griess test^50^.. The absorbance at 520 nm was measured using a Tristar lb^2^ ™ microplate reader (Berthold, Germany) after equal amounts of culture supernatants and Griess reagent were mixed and incubated at room temperature for 10 min to generate the colored diazonium salt. The NO inhibition percentage of each extract was calculated in relation to the LPS-induced inflammation group.

#### Western blotting analysis

Western blotting analysis of NQO1 protein expression: For NQO1 Western blotting, Control or extract-treated cell lysate proteins (50 µg/lane) were loaded onto a 10% polyacrylamide gel and separated by electrophoresis on a Biorad Tetra Cell (Biorad, USA) for 90 min at 110 volts.

Following electrophoresis, proteins were blotted onto a nitrocellulose membrane on a Biorad transfer module (60 min at 100 volts). After transfer, each blot was subjected to splicing according to the molecular weight of the studied targets, to incubate the resulting strips with the corresponding antibody against the target. Membrane strips were blocked with 5% non-fat milk and incubated with primary NQO1 antibody (Elabscience, USA) or β-actin (Thermofisher Scientific, USA) at 4 °C/overnight with gentle rolling on a tube roller. Following 3 × 5 min washes with Tris-buffered saline with Tween 20 (TBST), membrane strips were incubated with appropriate horseradish peroxidase (HRP)-conjugated secondary antibodies for 1 h on a tube roller. After secondary antibody incubations, membrane strips were washed again for 3 × 5 min washes in TBST. Protein bands were visualized using an enzyme chemiluminescence kit (Pierce, USA). Bands were imaged and analyzed using a Biospectrum Imager (UVP, UK) powered by Visionworks Acquisition and Analysis software package (version 8.20.17096.9551, Analytik Jena, Germany).

Western blotting of the anti-inflammatory marker iNOS: RAW264.7 cells were seeded at 1.5 × 10^6^ cells/well of 6-well plates. The cells were incubated overnight and then treated with the extracts (100 µg/mL, final DMSO concentration is 0.1% v/v) in the absence or the presence of 100 ng/mL of LPS. Western blotting was employed to assess the relative protein expression of the pro-inflammatory marker iNOS, as previously described^46^.

Following 24 h of treatment, RAW264.7 cells were washed using ice-cold DPBS and scraped into homogenization buffer. Cell suspensions were sonicated (30% amplitude/10 s) on ice. Sonicates were centrifuged (12,000 g/5 min). Supernatants were assessed for iNOS protein expression as reported before^44^. Following blot development with enzyme chemiluminescence, images were acquired using Biospectrum Imager (UVP, UK). A densitometric analysis of the obtained iNOS bands was performed on Visionworks Acquisition and Analysis software (version 8.20.17096.9551, Analytik Jena, Germany).

## Results and discussion

### Metabolome profiling of the studied *Salvia* species

For a comprehensive overview of the secondary metabolome of the studied *Salvia* species, UPLC-HRMS/MS was employed in both negative and positive ionization modes. The analysis of the selected *Salvia* extracts revealed notable qualitative and quantitative variations, as evidenced by their respective base peak chromatograms in both ionization modes (Supplementary Fig. S1&S2).

**Fig. 1:**
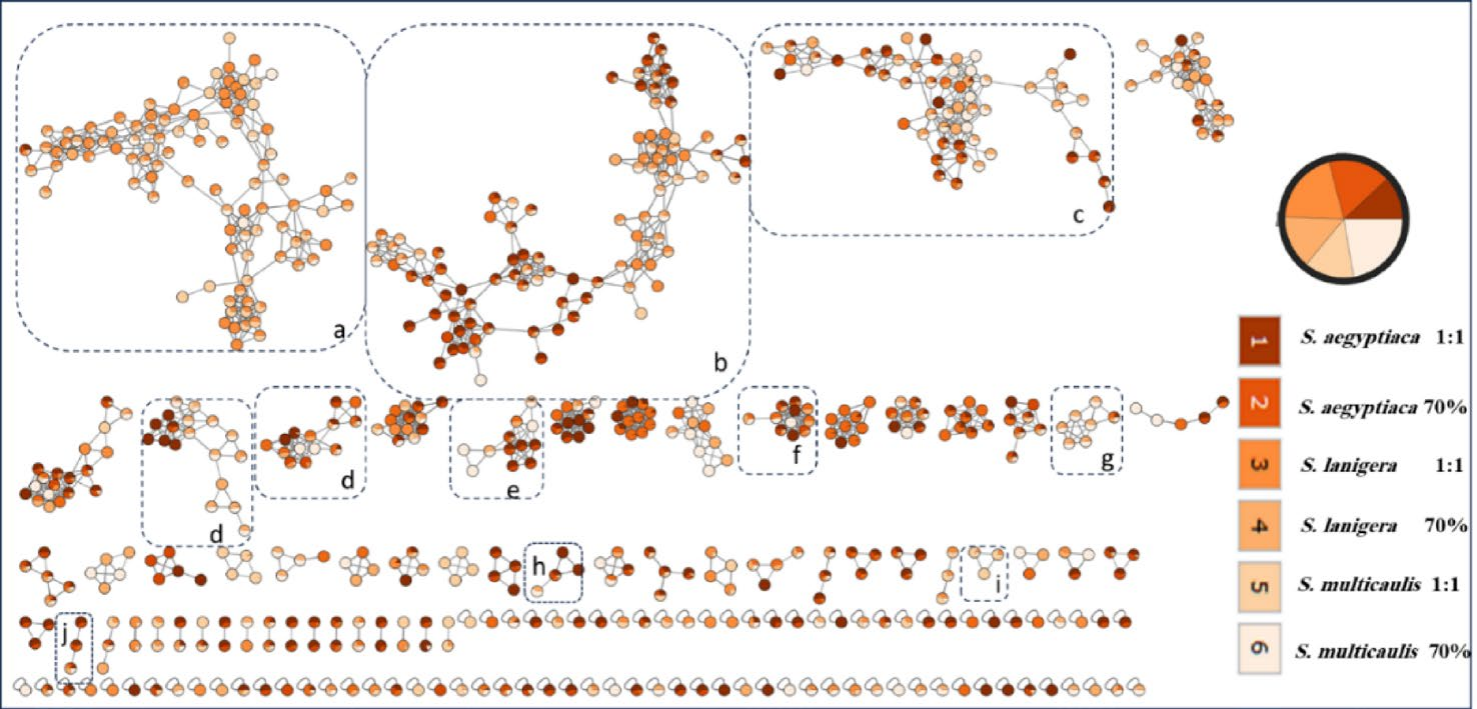
Full FBMN. created using UPLC-HRMS/MS data in the negative ionization mode from the studied *Salvia* extracts.

Following, two FBMNs were constructed to better visualize the metabolome diversity of the selected *Salvia* species and to highlight the unique chemical profile of *S. multicaulis* (Fig. 1 and Supplementary Fig. S3 & S4). In the constructed networks, node attributes were assigned such that the node color corresponded to the species, and pie charts were used to illustrate the distribution of each ion among the three species. The negative FBMN comprised 782 nodes grouped into 59 clusters, with 354 appearing as singletons (Fig. 1 and Supplementary Fig. S3). Similarly, the positive FBMN contained 1267 nodes organized into 744 clusters, with 666 represented as self-looped nodes (Supplementary Fig. S4).

In total, 218 metabolites were tentatively annotated, including phenolic acids (benzoic and cinnamic acid derivatives), flavonoid *O*-/*C*-glycosides, lignans, terpenoids (monoterpenes, diterpenes, sesquiterpenoids, megastigmanes, and triterpenes), and other miscellaneous compounds (Supplementary Table S1). Dereplication of the detected metabolites was initially performed based on their chromatographic and spectral characteristics (parent mass, molecular formula, and fragmentation pattern) (Supplementary Table S1), and was further refined using FBMNs in combination with *in silico* fragmentation trees generated by SIRIUS^16^.

#### Cinnamic acid derivatives

**Fig. 2:**
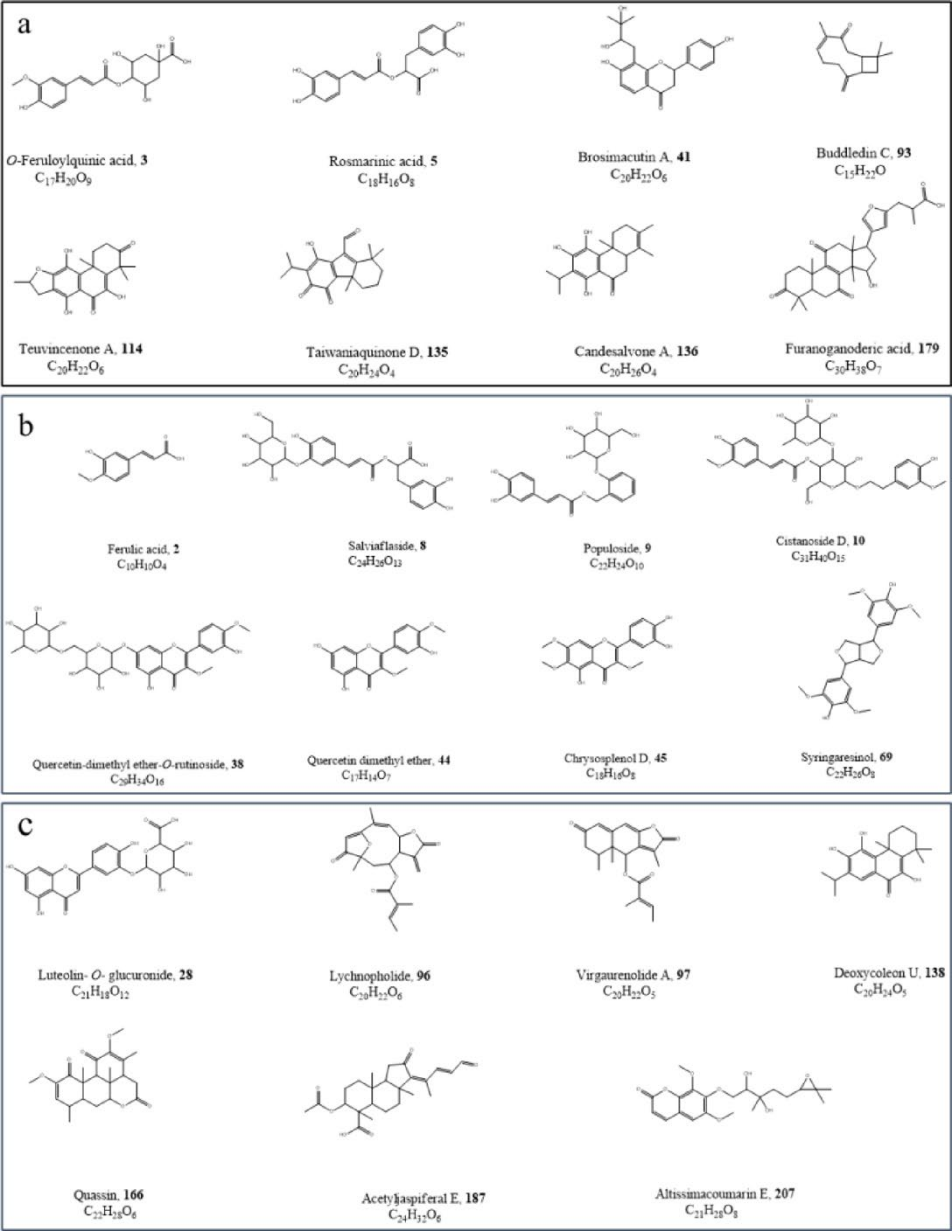
Representative examples of compounds reported in the *Salvia* species: (**a**) *S. multicaulis*, (**b**) *S.aegyptiaca*, (**c**) *S. lanigera*.

Members of the Lamiaceae family are well recognized for their richness in cinnamic acids and derivatives^47^. Eleven cinnamic acid derivatives were widely distributed among the studied species, as observed in the negative FBMN **(**Fig. 1**and Supplementary Fig. S3**). The detected cinnamic acid derivatives were identified as free acids, glycosides, or sulfated analogs. Annotated cinnamic acid derivatives included previously reported metabolites such as caffeic acid (**4**, *m/z* 179.035 [M - H]^−^, C_9_H_8_O_4_), salviaflaside (**8**, *m/z* 521.1296 [M - H]^−^, C_24_H_26_O_13_), methyl rosmarinate (**12**, *m/z* 373.0935 [M - H]^−^, C_19_H_18_O_8_)^48^, and whereas rosmarinic acid (**5**, *m/z* 359.0771 [M - H]^−^, C_18_H_16_O_8_) was predominant in *S. multicaulis* **(**Fig. 2a**)**. Pharmacological studies have demonstrated that rosmarinic acid and its derivatives exhibit a wide range biological activities, including antioxidant, anti-inflammatory, anticancer, antimicrobial, and anti-apoptotic effects^49^.

In addition, several cinnamic acid derivatives were reported here for the first time in *Salvia* species **(**Fig. 2**and Supplementary Fig. S3**), such as ferulic acid (**2**, *m/z* 193.0504 [M - H]^−^, C_10_H_10_O_4_), *O*-feruloylquinic acid (**3**, *m/z* 367.1033 [M - H]^−^, C_17_H_20_O_9_), hydroxy-allylbenzene-*O*-hexoside (**6**, *m/z* 311.1135 [M - H]^−^, C_15_H_20_O_7_), clinopodic acid B **(7**, *m/z* 373.0932 [M - H]^−^, C_19_H_18_O_8_), populoside (**9**, *m/z* 447.1297 [M - H]^−^, C_22_H_24_O_10_), cistanoside D (**10**, *m/z* 651.2287 [M - H]^−^, C_31_H_40_O_15_), and *O*-acetylmartyonoside (**11**, *m/z* 693.2407 [M - H]^−^, C_33_H_42_O_16_).

#### Flavonoids and their derivatives

Flavonoids, a major group of natural polyphenolic compounds, are highly regarded for their chemical diversity and broad spectrum of biological activities, including antioxidant, anti-inflammatory, and anticancer effects^50^. *Salvia* species are recognized as valuable sources of flavonoids, with flavones and flavonols as the predominant subclasses, particularly glycosides of apigenin, luteolin, and quercetin^47,51^.

Flavonoids were abundantly present in all the studied species, as revealed by both FBMNs (Fig. 1 and Supplementary Fig. S3& S4). The most abundant metabolites were flavonols (i.e., quercetin, kaemperfol, and isorhamnetin) and flavones (i.e., apigenin, luteolin, naringenin, hispidulin, diosmetin, and eriodictyol) occurring as *O-*/*C-* glycosylated, or free aglycones as featured in the constructed FBMNs (Supplementary Table S1). In total, 45 flavonoids were annotated, some of which were previously reported to exist in this genus (Supplementary Table S1).

Among the annotated flavonoids, *O*-glycosidic derivatives were the most abundant in **(**Fig. 1**)**, showing the typical fragmentation pattern of *O*-glycosidic cleavage of − 162 Da, − 146 Da, and − 132 Da, which correspond to *O*-hexoside, *O*-deoxyhexoside, and *O*-pentoside, respectively^52^. Annotated *O*-glycosidic flavonoids which were previously reported in *Salvia* included quercetin-*O*-hexoside (**23**, *m/z* 463.0882 [M- H]^−^, C_21_H_20_O_12_), isomers of apigenin-*O*-hexoside (**25**,** 26**,** 34**, *m/z* 431.0981 [M - H]^−^, C_21_H_20_O_10_), hispidulin-*O*-hexoside (**31**, *m/z* 461.1086 [M - H]^−^, C_22_H_22_O_11_) and quercetin-dimethyl ether-*O*-rutinoside (**38**, *m/z* 637.1761 [M- H]^−^, C_29_H_34_O_16_).

Additionally, the following tentatively assigned flavonoids were detected: eriodictyol -*O*-rutinoside (**17**, *m/z* 595.1663 [M - H]^−^, C_27_H_32_O_15_), naringenin -*O*-hexoside (**18**, *m/z* 457.1103 [M + Na]^+^, C_21_H_22_O_10_), hesperetin-*O*-hexoside (**19**, *m/z* 463.1245[M - H]^−^, C_22_H_24_O_11_), luteolin *O*- rutinoside (**21**, *m/z* 593.1504 [M - H]^−^ C_27_H_30_O_15_), luteolin methyl ether *O*-hexoside (**27**, *m/z* 461.1092 [M - H]^−^, C_22_H_22_O_11_), luteolin- *O*- glucuronide (**28**, *m/z* 463.0859 [M + H]^+^, C_21_H_18_O_12_), kaempferol-*O*-hexoside (**29**, *m/z* 447.0930 [M - H]^−^, C_21_H_20_O_11_), apigenin *O*-glucuronide (**30**, *m/z* 445.0779 [M - H]^−^, C_21_H_18_O_11_), isorhamnetin -*O*-hexoside (**32**, *m/z* 477.1034 [M - H]^−^, C_22_H_22_O_12_), luteolin -methyl ether *O*-glucuronide (**33**, *m/z* 475.0876 [M - H]^−^, C_22_H_20_O_12_), and quercetin dimethyl ether (**44**, *m/z* 329.0665 [M - H]^−^, C_17_H_14_O_7_). Additionally, luteolin-*O*-hexoside (**35**, *m/z* 447.0927 [M -H]^−^, C_21_H_20_O_11_) was found to be predominant in *S. multicaulis*.

In contrast, *C*-glycosidic flavonoids, which were rarely detected among the analyzed *Salvia* extracts, exhibited a distinctive fragmentation pattern characterized by sequential neutral losses of 18, 60, 90, and 120 Da, consistent with the established behavior of *C*-linked flavone glycosides^52^. Notably, one *C*-glycosidic flavonoid was annotated as apigenin di-*C*-hexoside (**16**, *m/z* 593.1506 [M - H]^−^, C_27_H_30_O_15_), in agreement with previous studies in *Salvia*^53^.

Furthermore, the negative FBMN clearly facilitated the identification of a couple of chalcone glycosides that had not been previously reported in *Salvia*. Among the annotated chalcone, isomers of pentahydroxy chalcone *O*-hexoside (**14&15**, *m/z* 449.1087[M- H]^−^, C_21_H_22_O_11_).

Finally, flavonoid aglycones were detected, including hesperetin (**40**, *m/z* 301.0717 [M - H]^−^, C_16_H_14_O_6_), which has been reported to inhibit LPS-induced oxidative stress *via* modulation of pro-oxidant and antioxidant enzymes^54^, brosimacutin A (**41**, *m/z* 381.1302 [M + Na]^+^, C_20_H_22_O_6_), chrysosplenol D (**45**, *m/z* 359.0770 [M - H]^−^, C_18_H_16_O_8_), which is known for its demonstrated anticancer properties, tricin (**52**, *m/z* 329.0671 [M - H]^−^, C_17_H_14_O_7_), which possesses a range of distinctive biological activities supporting its role in chemoprevention^55^, and cirsimaritin (**46**, *m/z* 313.0726 [M - H]^−^, C_17_H_14_O_6_), which was predominant in *S. multicaulis*.

#### Lignans

Besides the identified phenolic metabolites, the analyzed *Salvia* species were also found to contain lignans, as indicated by both FBMNs **(**Fig. 1, Supplementary Fig. S3 & S4), in agreement with previous studies^56,57^.

Annotated lignans included isomers of hydroxylariciresinol- *O*-hexoside (**58**,** 59**,** 62** *m/z* 561.1942 [M + Na]^+^, C_26_H_34_O_12_), pentahydroxy-methoxy oxy neolignan -*O*-hexoside (**60**, *m/z* 549.1935 [M + Na]^+^, C_25_H_34_O_12_), trihydroxy-dimethoxy-*O*-neolignan-*O* -hexoside (**61**, *m/z* 563.2090 [M + Na]^+^, C_26_H_36_O_12_), armandiside (**63**, *m/z* 575.2105 [M + Na]^+^, C_27_H_36_O_12_), and viscoloratin **(64**, *m/z* 589.1892 [M + Na]^+^, C_27_H_34_O_13_),

Additionally, fraxiresinol-*O* hexoside (**65**, *m/z* 565.1911 [M - H]^−^, C_27_H_34_O_13_), symplocosin (**66**, *m/z* 519.1868 [M - H]^−^, C_26_H_32_O_11_), acetoxypinoresinol -*O*-hexoside (**67**, *m/z* 577.1916 [M - H]^−^, C_28_H_34_O_13_), secoisolariciresinol (**68**, *m/z* 361.1657 [M - H]^−^, C_20_H_26_O_6_), syringaresinol (**69**, *m/z* 417.1542 [M - H]^−^, C_22_H_26_O_8_), saurufurin E (**70**, *m/z* 357.1346 [M - H]^−^, C_20_H_22_O_6_) and cinnamophilin (**72**, *m/z* 343.1554 [M - H]^−^, C_20_H_24_O_5_) were also identified.

Moreover, obovaten (**71**, *m/z* 339.1232 [M - H]^−^, C_20_H_20_O_5_) was predominantly detected in *S. multicaulis*, and has been reported to exhibit marked cytotoxic activity *in vitro* against P-388, KB16, A549, and HT-29 cell lines^58^.

#### Terpenoids

Similar to the previously mentioned bioactive flavonoids, *Salvia* species are well known for their richness in terpene lactones, mainly comprising sesquiterpenes, diterpenes, and triterpenes^59^, terpenoids exhibited better ionization in the positive ionization mode and were widely distributed among the studied species (Supplementary Fig. S4). Notably, megastigmanes (norisoterpenoids) and monoterpenoids were more prevalent in *S. multicaulis* and *S. lanigera* and reported for the first time in *Salvia* species **(**Fig. 2**)**.

##### Megastigmanes

Dereplicated megastigmanes included icariside of B10 and B6 (**73**,** 76** *m/z* 411.1987, 395.2035 [M + Na]^+^, C_19_H_32_O_8,_ C_19_H_32_O_7_) respectively, isomers of canangaionoside (**74**,** 75** *m/z* 425.1778 [M + Na]^+^, C_19_H_30_O_9_), and alangionosides I (**77**, *m/z* 529.2615 [M + Na]^+^, C_24_H_42_O_11_).

##### Monoterpenes

Annotated monoterpenes including iridoids derivative (Supplementary Fig. S4), such as swertiaside A (**78**, *m/z* 497.1625[M + H]^+^, C_23_H_28_O_12_) and lucidumoside D (**79**, *m/z* 591.2037[M + Na]^+^, C_27_H_36_O_13_).

##### Sesquiterpenoids

The Lamiaceae family contains a large number of sesquiterpenoid compounds, which are categorized into several groups based on their carbocyclic skeletons, including guaianolides, germacranolides, eudesmanolides, and caryophyllane^60,61^. Annotated sesquiterpenoids were distributed among all the studied species, belonging to the germacranolides, guaianolides, caryophyllane, and eudesmanolides classes (Supplementary Fig. S4). The annotated sesquiterpenoids were more abundant in *S. multicaulis*, which included karinolide 1(**90**, *m/z* 391.1397, [M - H]^−^, C_20_H_24_O_8_). Buddledin C (**93**, *m/z* 219.1744 [M + H]^+^, C_15_H_22_O), ineupatorolide A (**110**, *m/z* 389.1957[M + Na]^+^, C_20_H_30_O_6_), which is known for its remarkable antiplasmodial activity^62^, lychnopholide (**96**, *m/z* 359.1485 [M + H]^+^, C_20_H_22_O_6_), and virgaurenolide A (**97**, *m/z* 343.1537 [M + H]^+^, C_20_H_22_O_5_) were abundant in *S. lanigera* **(**Fig. 2c**)**.

##### Diterpenoids

Similar to the bioactive sesquiterpene discussed earlier, the Lamiaceae family is well known for their high content of diterpenoid^63,64^. Particularly in *S. multicaulis*, some diterpenoid were identified (Supplementary Fig. S3), including teuvincenone A (**114**, *m/z* 357.1341 [M + H]^+^, C_20_H_22_O_6_), taiwaniaquinone D (**135**, *m/z* 329.1746 [M + H]^+^, C_20_H_24_O_4_), candesalvone A (**136**, *m/z* 331.1904 [M + H]^+^, C_20_H_26_O_4_) **(**Fig. 2a**)**, methoxyvelutine C (**124**, *m/z* 393.1912 [M + H]^+^, C_21_H_28_O_7_), hydroxy-*0*- methyl rosmanol (**139**, *m/z* 375.1818 [M - H]^−^, C_21_H_28_O_6_), and tetraene-nor-friedelane-oic acid methylester (**150**, *m/z* 495.2749 [M - H]^−^, C_30_H_40_O_6_).

Deoxycoleon U (**138**, *m/z* 331.1897 [M + H]^+^, C_20_H_24_O_5_) was abundant in *S. lanigera* **(**Fig. 2c**)**. Moreover, methylcryptotanshinone (**143**, *m/z* 311.1646 [M + H]^+^, C_20_H_22_O_3_), previously reported in *Salvia* species such as *S. aegyptiaca*^65^, was also detected.

##### Triterpenoid

Triterpenes were widely distributed among the studied *Salvia* species, in accordance with previous reports^66,67]^ (Supplementary Fig. S3 & S4). The annotated triterpenoid included isomers of hederagenin (**183**,** 186** *m/z* 471.3482 [M-H]^−^, C_30_H_48_O_4_), maslinic acid (**189**,** 191** *m/z* 473.3626 [M + H]^+^, 471.3469 [M - H]^−^, C_30_H_48_O_4_), and oleanolic acid (**195**,** 197** *m/z* 455.3544 [M - H]^−^, C_30_H_48_O_3_). Additional triterpenoids included lupeone (**201**,** 202** *m/z* 425.3775 [M + H]^+^, C_30_H_48_O), 6-oxo-dihydropristimerol-23-oic acid (**175**, *m/z* 511.2700 [M - H]^−^, C_30_H_40_O_7_), echinocystic acid (**164**, *m/z* 471.3468 [M - H]^−^, 473.3626 [M + H]^+^, C_30_H_48_O_4_), furanoganoderic acid (**179**, *m/z* 509.2538[M - H]^−^ C_30_H_38_O_7_), and α-tocospiros (**204**, *m/z* 463.3785 [M + H]^+^, C_29_H_50_O_4_). Quassin **(166**, *m/z* 411.1772 [M + Na]^+^, C_22_H_28_O_6_**)** and acetyljaspiferal E (**187**, *m/z* 439.2092 [M + Na]^+^, C_24_H_32_O_6_**)** were abundant in *S. lanigera* **(**Fig. 2c**).**

Lastly, several other terpenoids were annotated in members of the *Salvia* genus (Supplementary Fig. S4), including meroterpenoids such as dimethyl-6-(1-hydroxyethyl)chroman-4-one (**203**, *m/z* 221.1170 [M + H]^+^, C_13_H_16_O_3_).

#### Miscellaneous

In addition to the metabolites previously identified, a few coumarins were annotated (Supplementary Table S1), including isomers of schinilenol (**210**,** 213** *m/z* 343.1552 [M -H]^−^, C_20_H_24_O_5_), and cordatolide E (**212**, *m/z* 359.1503 [M -H]^−^, C_20_H_24_O_6_), which is known for its anti-HIV activity^68^. Furthermore, one chromone was solely detected in *S. aegyptiaca* and was tentatively assigned as (dimethylallyl)- methoxy-methylchromone (**206**, *m/z* 259.1330 [M + H]^+^, C_16_H_18_O_3_). Altissimacoumarin E (**207**, *m/z* 407.1705 [M -H]^−^, C_21_H_28_O_8_) was abundant in *S. lanigera* **(**Fig. 2c**).**

### Integrated network pharmacology and *in silico* analysis

#### Molecular docking

**Fig. 3:**
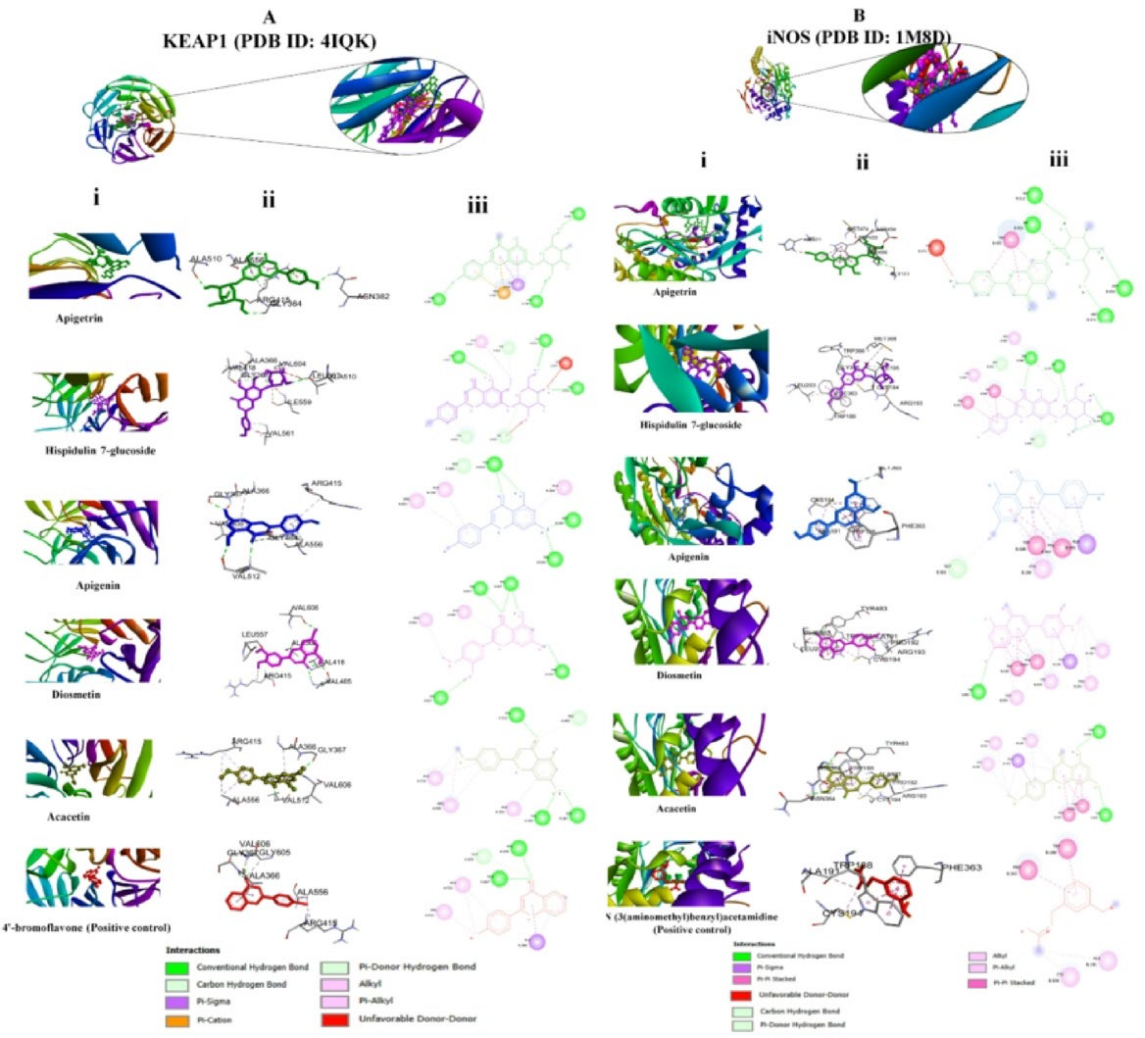
Comparative molecular docking analysis of the major compounds in LC-MS/MS-based metabolomics with KEAP1 (PDB ID: 4IQK) and iNOS (PDB ID: 1M8D). **(A)** Predicted binding modes inside the active site of 4IQK for the major compounds in LC-MS/MS-based metabolomics against KEAP1 compared to the docked 4‵-bromoflavone as a positive control **(i)** 3D docking poses showing ligand orientation inside the binding pocket; **(ii)** 2D interaction diagrams; **(iii)** The interaction types schematically, color-coded by bonding category. (**B)** Predicted binding modes inside the active site of 1M8D for the major compounds in LC-MS/MS-based metabolomics against iNOS compared to the docked N-(3-(aminomethyl)benzyl)acetamidine as a positive control; **(i)** 3D docking poses showing ligand orientation inside the binding pocket; **(ii)** 2D interaction diagrams; **(iii)** The interaction types schematically, color-coded by bonding category.

Accordingly, the selection strategy outlined in the Methodology was intended to balance predicted docking performance with UPLC-HRMS/MS -based chemical confidence and experimental relevance. The RMSD values of validation step obtained for the target proteins were 0.2968 Å for iNOS (PDB ID: 1M8D) and 1.0686 Å for KEAP1 (PDB ID: 4IQK). Both pose comparison analysis complexes exhibited RMSD values below the generally accepted threshold of 2.0 Å, indicating successful reproduction of the native binding conformations. Docking scores (binding affinities, ΔG) of all compounds were calculated using AutoDock Vina and compared with the standard 4‵-bromoflavone for KEAP1^6^ and N-(3-(aminomethyl)benzyl)acetamidine for iNOS^69^. Ten compounds exhibited superior docking scores with both receptors. Free binding energies are shown in Table 1. Predicted binding interactions in the 4IQK active site for ten compounds and 4’-bromoflavone are depicted in Fig. 3A, while predicted binding interactions in the 1M8D active site for ten compounds and N-(3-(aminomethyl)benzyl) acetamidine are depicted in Fig. 3B.

The compounds selected were all flavonoids (flavones). Molecular docking results predicted a favorable.

binding affinities of luteolin *O*-glucuronide toward KEAP1 (ΔG = − 11 kcal/mol) compared to the positive control (4’-bromoflavone for KEAP1, ΔG = − 8.5 kcal/mol). Similarly, hispidulin 7-glucoside exhibited favorable predicted interactions with iNOS (ΔG = − 10.5 kcal/mol) compared to the positive control (N-(3-(aminomethyl)benzyl) acetamidine for iNOS, ΔG = − 7.1 kcal/mol). Aglycone flavones such as acacetin, diosmetin, and apigenin also displayed favorable binding energies that showed potential contribution to the bioactivities (Fig. 3A and B).

**Table 1:** Docking results of the top predicted compounds identified by LC‑MS/MS against KEAP1 and iNOS compared with their respective positive controls^70,71^.

No.	Compounds identified from LC-MS/MS	Free Binding Energy (∆G) (kcal/mol)	
		KEAP1	iNOS
1	Luteolin *O*-glucuronide	−11 ± 0.0	−10.2 ± 0.1
2	Hispidulin 7-glucoside	−9.5 ± 0.1	−10.5 ± 0.0
3	Acacetin	−9.2 ± 0.0	−9.9 ± 0.0
4	Diosmetin	−9.2 ± 0.0	−9.8 ± 0.1
5	Apigenin	−9 ± 0.1	−8.8 ± 0.0
6	Apigenin 7-*O*-glucoside (Apigetrin)	−10 ± 0.0	−7.9 ± 0.0
7	Apigenin dimethyl ether (Apigenin 7,4’-dimethyl ether)	−8.7 ± 0.1	−9.2 ± 0.0
8	Salvigenin	−8.9 ± 0.1	−8.3 ± 0.0
9	Cirsimaritin	−8.8 ± 0.0	−7.7 ± 0.0
10	Velutin	−8.7 ± 0.0	−7.6 ± 0.1
11	4’-bromoflavone (Positive control)	−8.5 ± 0.2	-
12	N-(3-(aminomethyl)benzyl)acetamidine (Positive control)	-	−7.1 ± 0.1

#### Pharmaco-informatics: Screening with the rule of five

Ten analyzed metabolites are candidates for drug likeness based on Lipinski’s rule. Of these compounds, seven met the criteria fully or with only one minor violation, indicating favorable physicochemical properties for absorption (Supplementary Table S2). Compounds such as acacetin, diosmetin, and apigenin exhibited optimal molecular weights, hydrogen bonding profiles, and topological polar surface areas (TPSA), allowing their suitability for further pharmacokinetic exploration.

In contrast, glycosylated derivatives like luteolin *O*-glucuronide and hispidulin 7-glucoside failed due to excessive hydrogen bond donors and acceptors, as well as elevated TPSA values, which may hinder membrane permeability. These findings underscore the importance of balancing needs to balance polarity and molecular flexibility in the early stages of natural product-based drug development.

#### Pharmacokinetics and toxicity of major compounds tentatively identified from *Salvia* species

The pharmacokinetic and toxicity profiles of the major compounds tentatively identified from *Salvia* species indicate a favorable safety margin, accompanied by diverse absorption and metabolic characteristics. All ten compounds are in toxicity class 5, which means they have low acute toxicity (LD₅₀ values between 2500 and 5000 mg/kg). This makes them good candidates for therapeutic development. However, gastrointestinal (GI) absorption is variable among flavonoids. For example, aglycone flavonoids like apigenin and diosmetin are absorbed well, but glycosylated derivatives like luteolin *O*-glucuronide are not absorbed well, probably because they are more polar and heavier.

The blood-brain barrier (BBB) was only able to let some compounds through, like salvigenin and apigetrin. This suggests limited central nervous system penetration for most compounds. The P-glycoprotein (Pgp) substrate status and CYP450 inhibition profiles further differentiate these compounds: several flavonoids act as inhibitors of CYP3A4, CYP2C19, and CYP2D6, which may influence drug–drug interactions and metabolic clearance. These results highlight the necessity of incorporating ADMET screening early in natural product-based drug discovery to prioritize candidates with advantageous pharmacokinetic and safety profiles (Table Supplementary S3).

#### Target identification and analysis

The major bioactive compounds were obtained from *Salvia* species, including 231 targets of ten bioactive compounds, which were discovered through the Swiss Target Prediction. Potential therapeutic targets for chemoprevention and anti-inflammation were obtained from the GeneCards database, including 390 and 1066, respectively. We found the GeneChip GSE230608 focuses on the KEAP1–NRF2 signaling in HepG2 cells, showing how KEAP1 deficiency drives NRF2 activation that centers on oxidative stress. While GSE193336 GeneChip profiles human macrophages under basal and LPS-stimulated inflammatory conditions, capturing gene expression changes that underline innate immune activation. The GEO datasets GSE230608 and GSE193336 were both generated using RNA-seq, but under different experimental designs.

**Fig. 4:**
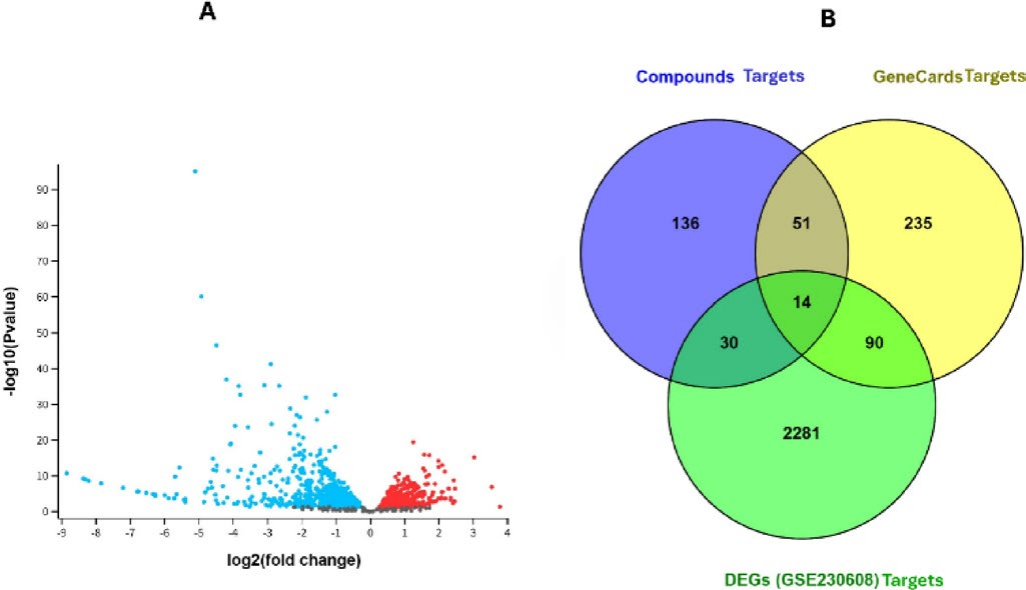
**(A)** Differentially expressed genes in KEAP1-NRF2-related chemoprevention. According to the standard of FDR < 0.05 & |log FC| > 1, red represents up-regulated genes and blue represents down-regulated genes. **(B)** A Venn diagram of the intersection of potential targets of GeneCards, potential targets of GOE2R (DEGs of GSE230608), and the intersection of major compounds of *Salvia* species differentially expressed genes.

In GSE230608, HepG2 cells were transduced with CRISPR guides targeting KEAP1 compared to control sgAAVS168, with multiple replicates collected for each condition to capture differential gene expression linked to NRF2-mediated lysosomal biogenesis. A total of 12,655 genes were identified, with 1382 up-regulated genes and 1243 down-regulated genes of DEGs. The volcano plot (Fig. 4A) of DEGs was shown. The Venny diagram intersection obtained a total of 104 primary relevant targets associated with KEAP1, which appeared at least twice in the GeneCards databases and DEGs of GSE230608 in the GEO database. These were intersected with 231 targets of ten major compounds of *Salvia* species.

Ultimately,14 potential therapeutic targets were identified (Fig. 4B). In contrast, GSE193336 profiled primary human macrophages from four independent blood donors, with four unstimulated controls and four LPS-stimulated samples, providing a balanced design to assess transcriptomic changes during inflammatory activation. Together, these datasets comprise carefully controlled sample sets that enable robust comparisons between experimental and control groups in both liver cell signaling and immune response contexts.

**Fig. 5:**
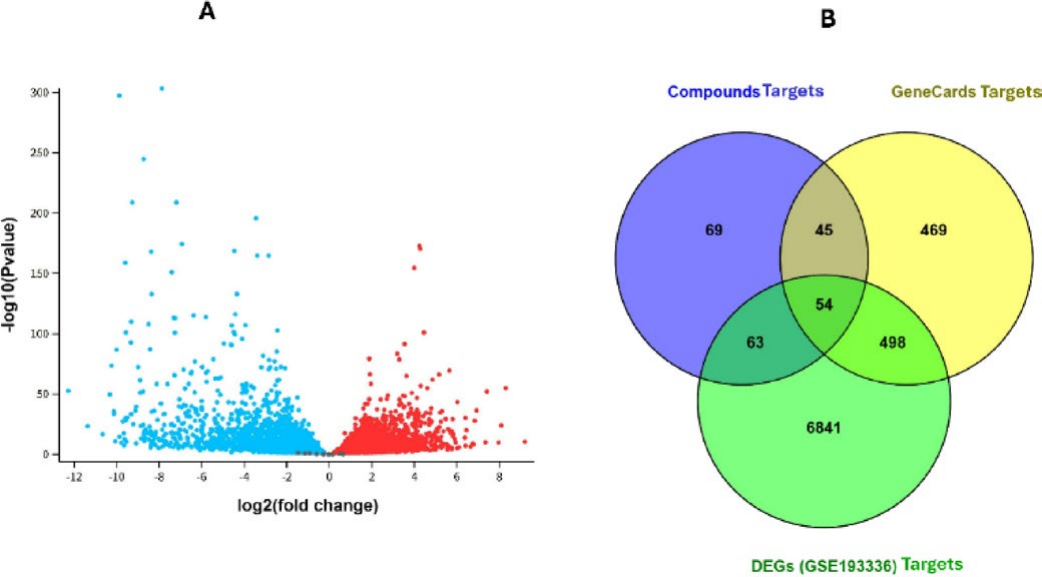
**(A)** Differentially expressed genes in iNOS related anti-inflammatory. According to the standard of FDR < 0.05 & |log FC| > 1, red represents up-regulated genes and blue represents down-regulated genes, **(B)** A Venn diagram of the intersection of potential targets of GeneCards, potential targets of GOE2R (DEGs of GSE193336), and the intersection of major compounds of *Salvia* species differentially expressed genes.

Totally, 17,975 genes were identified, with 4378 up-regulated genes and 4759 down-regulated genes of DEGs. The volcano plot (Fig. 5A) of DEGs was shown. The Venny diagram intersection obtained a total of 552 primary relevant targets associated with iNOS, which appeared at least twice in the GeneCards databases and DEGs of GSE193336 in the GEO database. These were intersected with 231 targets of ten major compounds of *Salvia* species. Ultimately, 54 potential therapeutic targets were identified (Fig. 5B). A protein-protein interaction network reveals the intricate web of connections between various proteins in a cell, crucial for understanding cellular functions and disease mechanisms^72^.

#### Identification of hub genes

**Fig. 6:**
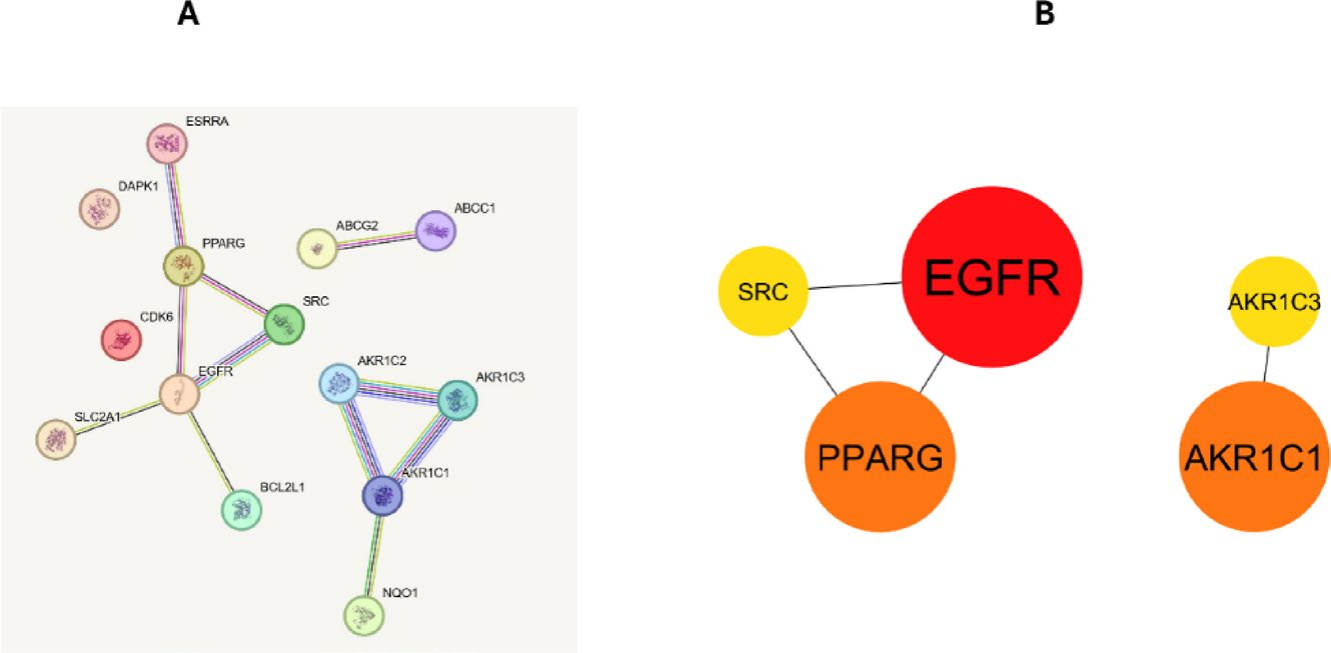
Protein–protein interaction (PPI) networks of major compounds from *Salvia* species associated with KEAP1-mediated chemoprevention. **(A)** PPI-constructed network showing the interactions of major *Salvia* compounds with KEAP1-related proteins. Nodes represent proteins, and edges represent predicted interactions, color-coded by interaction type. **(B)** Simplified PPI network highlighting the top five hub genes, forming two functional subclusters. They were identified by the Cytoscape plugin cytoHubba. Node colors reflect degree centrality ranking.

A PPI network with 14 intersecting targets was generated (Fig. 6A); the greater the influence on KEAP1-associated chemoprevention, the larger and darker the node. A total of 14 shared target genes were imported into the STRING database^73^ for the construction of the PPI network. In this study, the 0.7 confidence (high) was set as the default threshold^74^. To obtain a better understanding, the results were further analyzed by Cytoscape 3.10.3 software^74^, and a PPI network with 14 nodes and 11 edges was obtained. The hub target possessed a higher degree of (DOF) and was more likely to play a critical role in the network of *Salvia* species on KEAP1-associated chemoprevention, such as SRC proto-oncogene non-receptor tyrosine kinase (SRC), epidermal growth factor receptor (EGFR), peroxisome proliferator-activated receptor gamma (PPARG), aldo-keto reductase family 1 member C1 (AKR1C1), and aldo-keto reductase family 1 member C3 (AKR1C3).

The average DOF of the PPI network was 2, and the top five hub targets above the average DOF were shown in Fig. 6B. The hub genes did not converge into a single continuous network but instead formed two distinct subclusters in Cytoscape, with SRC–EGFR–PPARG constituting a signaling–transcriptional axis linked to oxidative stress and anti-inflammatory regulation and AKR1C1–AKR1C3 forming a metabolic detoxification module tied to Nrf2-mediated electrophilic stress neutralization. High-confidence STRING interactions highlight functional divergence in the KEAP1-related network, and keeping the fragmented structure preserves biological accuracy by showing how *Salvia*-responsive genes act through distinct parallel mechanisms^73^.

**Fig. 7:**
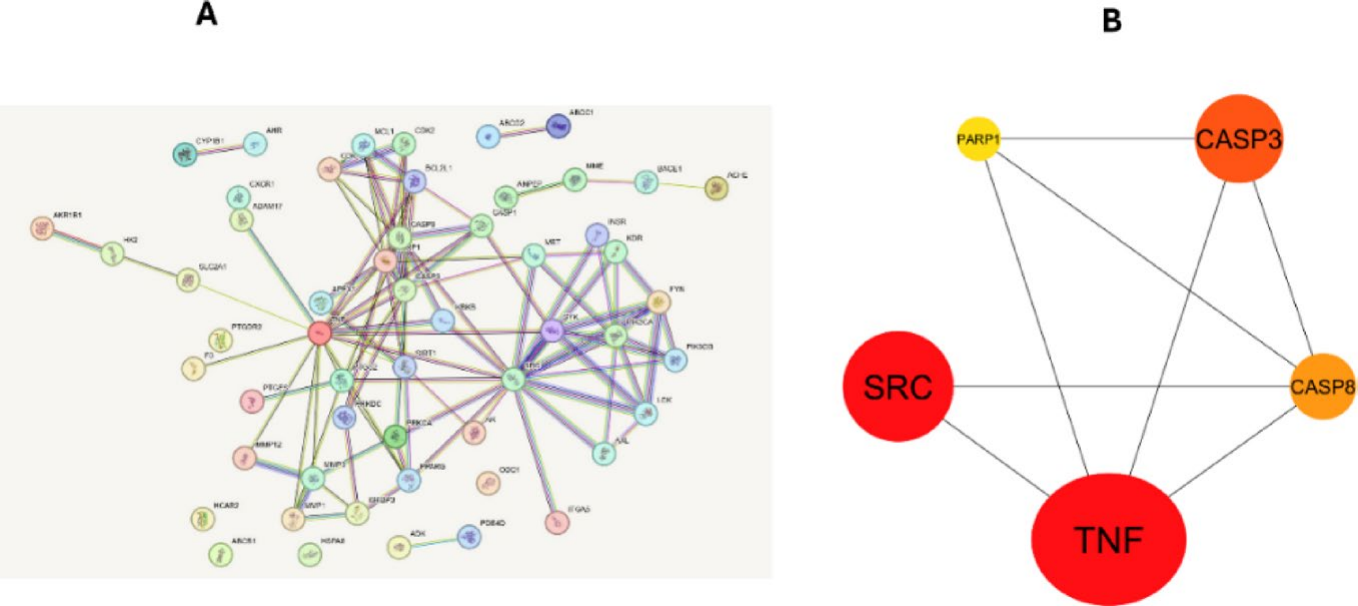
Protein–protein interaction (PPI) networks of major compounds from *Salvia* species associated with iNOS-mediated anti-inflammatory activity. **(A)** PPI-constructed network showing the interactions of major *Salvia* compounds with iNOS-related proteins. Nodes represent proteins, and edges represent predicted interactions, color-coded by interaction type; **(B)** Simplified PPI network highlighting the top five hub genes identified by the Cytoscape plugin cytoHubba. They were identified by the Cytoscape plugin cytoHubba. Node colors reflect degree centrality ranking.

A PPI network with 54 intersecting targets was generated (Fig. 7A); the greater the influence on iNOS-associated anti-inflammatory, the larger and darker the node. A total of 54 shared target genes were imported into the STRING database^73^ for the construction of the PPI network. In this study, the 0.7 confidence (high) was set as the default threshold. It was analyzed by Cytoscape 3.10.3 software^74^, and a PPI network with 54 nodes and 104 edges was obtained. The hub target possessed a higher DOF and was more likely to play a critical role in the network of *Salvia* species on iNOS-associated anti-inflammatories, such as tumor necrosis factor (TNF), a central cytokine driving inflammation and apoptosis; SRC proto-oncogene (SRC), a non-receptor tyrosine kinase involved in signal transduction; caspase 3 (CASP3) and caspase 8 (CASP8), both critical executioners of programmed cell death; and poly(ADP-ribose) polymerase 1 (PARP1), a DNA repair enzyme often cleaved during apoptosis. Their interactions suggest a coordinated role in inflammatory signaling and apoptotic regulation. The average DOF of PPI network was 5.158, and the top five hub targets above the average DOF were shown in Fig. 7B.

#### Gene ontology and pathway enrichment analysis

To clarify the molecular mechanisms through which active compounds enhance KEAP1-NRF2- associated chemoprevention, GO annotations and KEGG pathway analysis were conducted on a set of 14 targets associated with KEAP1-related chemoprevention. GO analysis recognized 57 biological processes (BP), which include cellular response to hydrogen peroxide, positive regulation of reactive oxygen species metabolic process, and response to xenobiotic stimulus that confirm their relevance to redox signaling and detoxification^75^; 13 cellular components (CC), which include membrane raft, extracellular exosome, and basolateral plasma membrane that suggest compartmentalized signaling and transport^76^; and 35 molecular functions (MF), such as aldo-keto reductase (NADPH) activity, ABC-type transporter activity, and oxidoreductase activity.

**Fig. 8:**
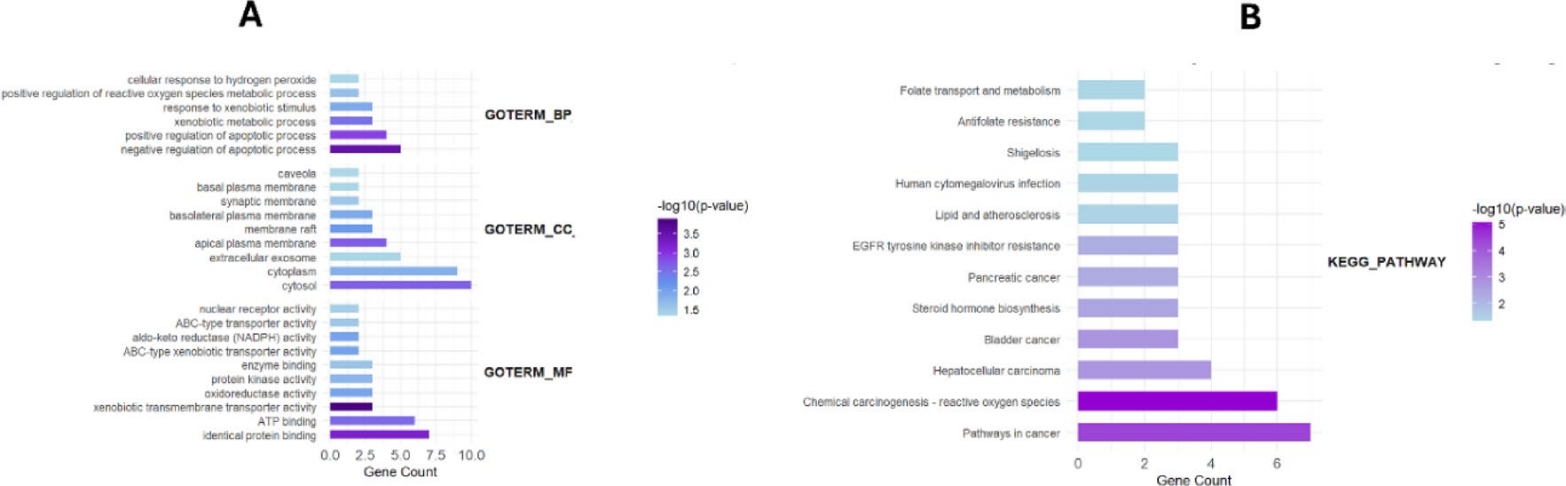
GO and KEGG analyses of major potential therapeutic targets for *Salvia* species on KEAP1-associated chemoprevention **(A)** The highly enriched entries for each GO category (BP, CC, and MF), **(B)** Results for KEGG analysis of highly intersecting targets. Analyses were performed using DAVID^28^, and pathways were adapted from KEGG (https://www.kegg.jp)^29,30^, with permission.

These are showing enzymatic roles in the metabolism of reactive compounds^77^. Applying the cutoff value *p* < 0.05, the top 10 GO annotations (BP, CC, and MF) were selected to draw bar plots (Fig. 8A). KEGG analysis predicted 20 pathways regarding KEAP1-associated chemoprevention, which include chemical carcinogenesis, reactive oxygen species, EGFR tyrosine kinase inhibitor resistance, lipid and atherosclerosis, and steroid hormone biosynthesis, further supporting the dataset’s connection to inflammation, cancer, and metabolic regulation^77^. The top KEGG-enriched pathways were categorized in Fig. 8B. While the enrichment analysis revealed ten highly significant GO terms and KEGG pathways that align with the anti-inflammatory role of inducible nitric oxide synthase (iNOS).

**Fig. 9:**
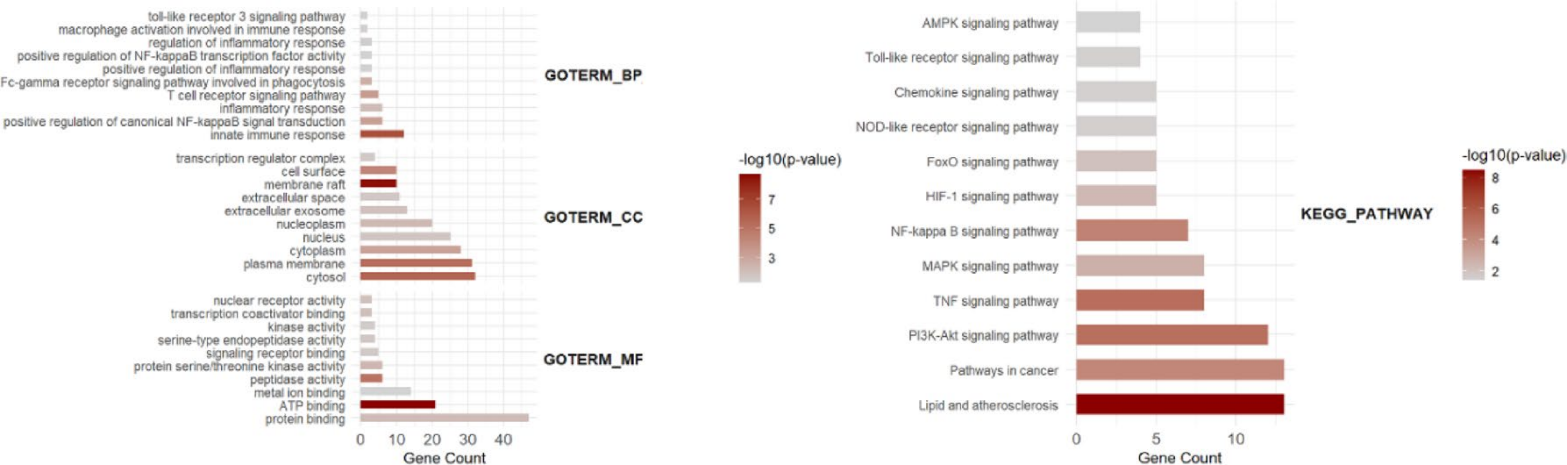
GO and KEGG analyses of major potential therapeutic targets for *Salvia* species on iNOS-associated anti-inflammatory **(A)** The highly enriched entries for each GO category (BP, CC, and MF). **(B)** Results for KEGG analysis of highly intersecting targets. Analyses were performed using DAVID^28^, and pathways were adapted from KEGG (https://www.kegg.jp)^29,30^, with permission.

GO annotations and KEGG pathway analysis were conducted on a set of 54 targets associated with iNOS-related anti-inflammation. GO analysis recognized 258 biological processes (BP), which include toll-like receptor 3 signaling, macrophage activation, and positive regulation of NF-κB transcription factor activity that enhance immunity and lead to iNOS induction^78^; 39 cellular components (CC), which include membrane raft, extracellular exosome, and basolateral plasma membrane that support signaling and transport^79^; and 105 molecular functions (MF), such as kinase activity, transcription coactivator binding, and serine-type endopeptidase activity suggest having regulation control over inflammatory cascades^80^. Applying the cutoff value *p* < 0.05, the top 10 GO annotations (BP, CC, and MF) were selected to draw barplots (Fig. 9A). KEGG analysis predicted 104 pathways regarding iNOS-related anti-inflammatory, which include Toll-like receptor signaling, NF-κB signaling, TNF signaling, and AMPK signaling, further supporting the dataset’s involvement in inflammation resolution and metabolic adaptation^81^. The top 10 KEGG-enriched pathways were categorized in Fig. 9B. The outcomes of these analyses provide insights into the functional roles of essential target genes and highlight significant pathways associated with the compounds under investigation.

#### Confirmation of hub target by molecular docking

**Fig. 10:**
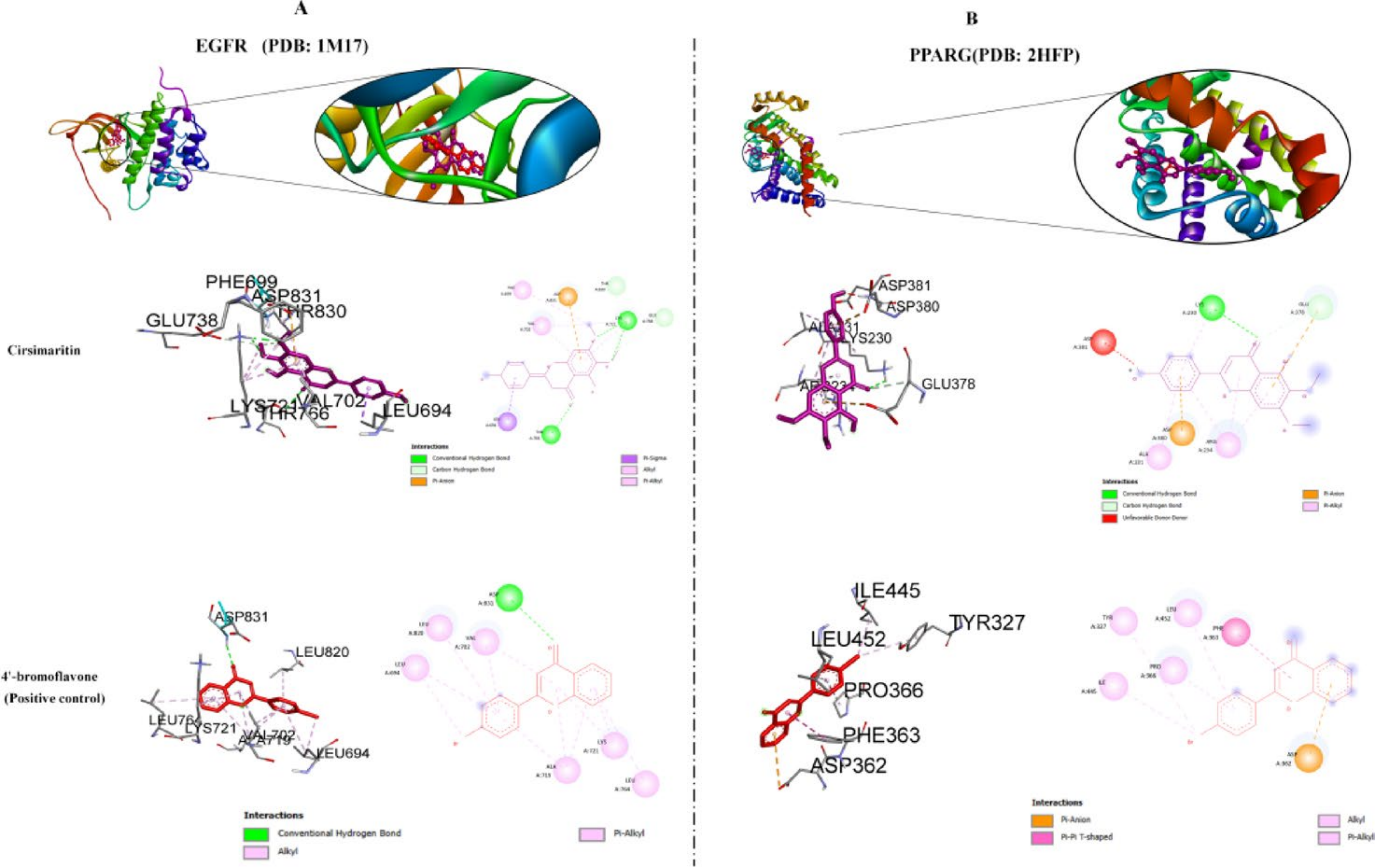
2D and 3D representations of the predicted binding modes of cirsimaritin and the positive control 4′-bromoflavone with EGFR and PPARG crystal structures. **(A)** EGFR (PDB ID: 1M17): Docking poses of cirsimartin (rose) compared to 4′-bromoflavone (red), showing ligand orientation within the binding pocket. The 2D interaction diagram highlights hydrogen bonds, hydrophobic contacts, and π interactions with key residues such as PHE699, GLU738, LEU694, and ASP831. **(B)** PPARG (PDB ID: 2HFP): Docking poses of cirsimaritin (rose) compared to 4′-bromoflavone (red), showing ligand orientation within the binding pocket. The 2D interaction diagram highlights hydrogen bonds, hydrophobic contacts, and π interactions with key residues, including GLU378 and ASP362.

**Fig. 11:**
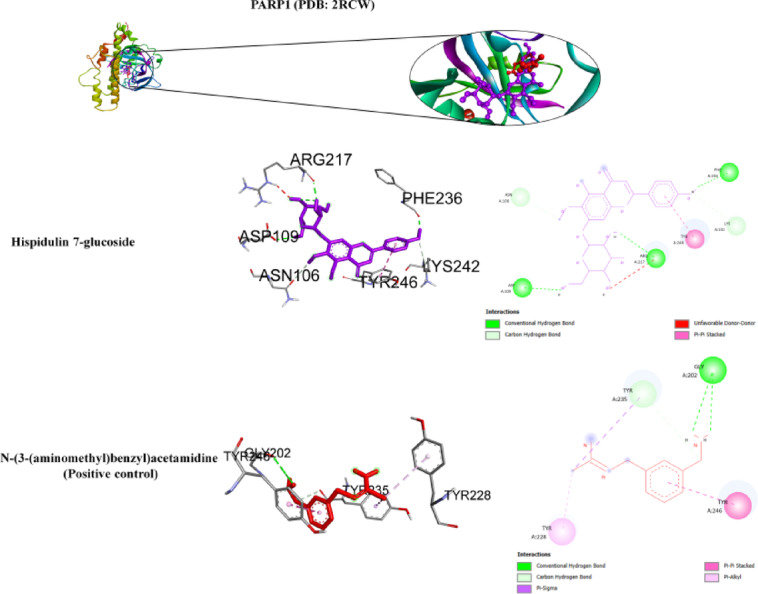
2D and 3D representations of the predicted binding modes of Hispidulin-7-glucoside (purple) and the positive control N-(3-(aminomethyl)benzyl) acetamidine (red) with PARP1 crystal structure (PDB ID: 2RCW). The docking visualization shows ligand orientation within the PARP1 binding pocket. Hispidulin 7-glucoside interacts with residues ARG217, ASP109, ASN106, PHE236, LYS242, and TYR246, while the positive control engages TYR228, and TYR235. The 2D interaction diagrams summarize the bonding categories schematically, color-coded for clarity.

**Fig. 12:**
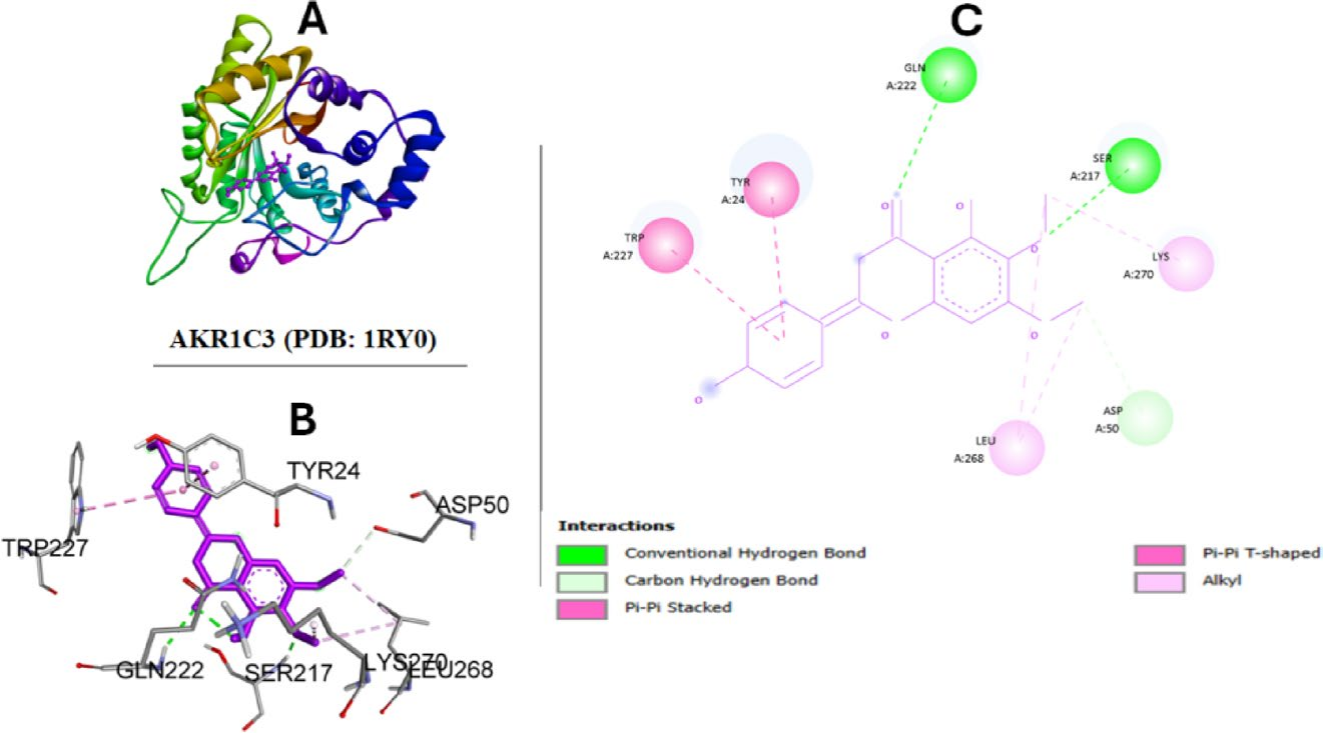
Binding interactions of cirsimaritin with AKR1C3 (PDB ID: 1RY0). **(A)** Ribbon representation of AKR1C3 showing cirsimaritin bound within the active site. **(B)** Three-dimensional interaction view of the binding pocket illustrating hydrogen-bond interactions with ASP50, SER217, and GLN222, along with π–π stacking interactions involving TRP227. **(C)** Two-dimensional interaction diagram depicting cirsimaritin and surrounding residues, highlighting conventional hydrogen bonds (green), carbon hydrogen bonds (light green), π–π stacking interactions (pink/magenta), and hydrophobic contacts (light purple).

**Fig. 13:**
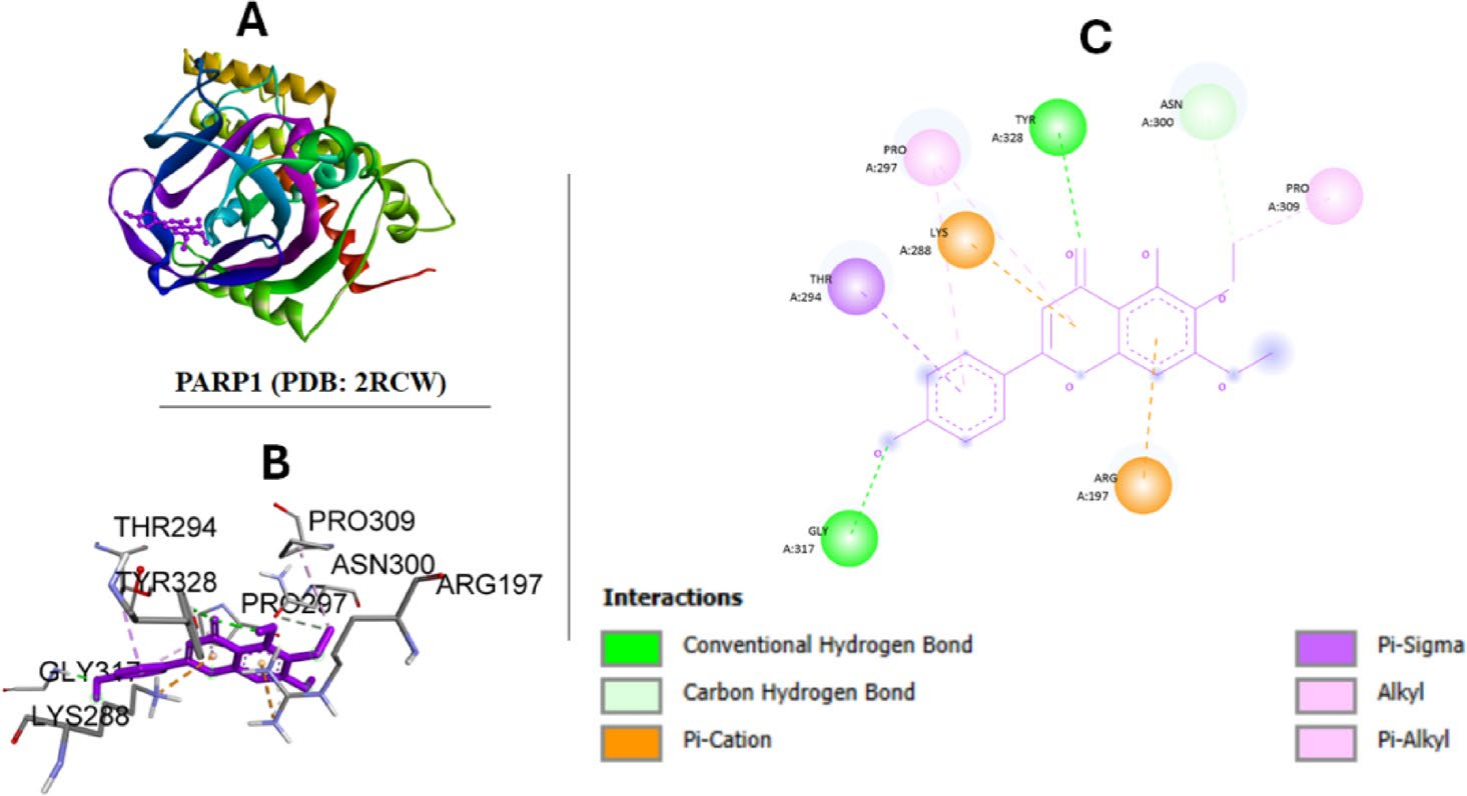
Binding interactions of cirsimaritin with PARP1 (PDB ID: 2RCW). **(A)** Ribbon representation of PARP1 showing cirsimaritin bound within the active site.** (B)** Three-dimensional interaction view of the binding pocket illustrating hydrogen-bond interactions with THR294 and ASN300, as well as hydrophobic and π-interactions involving LYS328, PRO309, and ARG197. **(C)** Two-dimensional interaction diagram of cirsimaritin and surrounding residues, highlighting conventional hydrogen bonds (green), carbon hydrogen bonds (light green), π–cation interactions (orange), π–sigma interactions (purple), alkyl interactions (light pink), and π–alkyl interactions (pink).

All poses comparison analyses exhibited RMSD values below the generally accepted threshold of 2.0 Å, indicating successful reproduction of the native binding conformations (Supplementary Table S5). To ensure the validity of the drug-target interactions, the molecular docking analysis focused on the five hub proteins as selected targets. In this study, the stability or strong inhibition of the ligand-receptor binding was assessed based on the binding energies between the ligand and protein. Binding energies lower than − 6.0 kcal/mol were considered indicative of a favorable binding potential, suggesting a stable ligand–protein complex and a higher likelihood of effective interaction^82^. Docking scores (binding affinities, ΔG) of major compounds *Salvia* species were calculated using AutoDock Vina and compared with the standard 4’-bromoflavone for KEAP1^6^ and N-(3-(aminomethyl)benzyl)acetamidine for iNOS^69^ were evaluated and represented in Table 2 & Table 3. Compounds predicted to have selective binding to targets associated with KEAP1–NRF2 chemoprevention are apigenin 7-*O*-glucoside (apigetrin), salvigenin, and cirsimaritin, which predicted favorable binding affinity toward key regulators including EGFR, PPARG, AKR1C1, AKR1C3, and NQO1, suggesting their potential in the activation of antioxidant and detoxification pathways. Cirsimaritin suggested favorable predicted binding affinities toward EGFR (− 9.8 kcal/mol), PPARG (− 8.4 kcal/mol), and AKR1C3 (− 10.9 kcal/mol) when compared with the standard 4’-bromoflavone EGFR (− 8.7 kcal/mol), PPARG (− 7.3 kcal/mol), and AKR1C3 (− 6.3 kcal/mol). These docking results suggest a potential interaction profile consistent with the modulation of NRF2-linked cytoprotective pathways (Fig. 10)^83,84^. Similarly, luteolin *O* -glucuronide, hispidulin 7-glucoside, apigenin 7-*O*-glucoside (apigetrin), and cirsimaritin demonstrated favorable predicted binding profiles against iNOS-related targets such as TNF, CASP3, CASP8, PARP1, and iNOS, supposing their capacity to suppress nitric oxide–driven inflammatory cascades^85^. Docking analysis suggested that hispidulin 7-glucoside may exhibit favorable predicted binding affinities toward iNOS, and PARP1 (− 10.5, and − 9.6 kcal/mol, respectively) in comparison to the standard N-(3-(aminomethyl)benzyl)acetamidine (−7.1 and − 6.7, respectively) (Fig. 11). While apigenin 7-*O*-glucoside (apigetrin), and cirsimaritin showed predicted binding activity across both antioxidant- and inflammation-related target sets, highlighting their potential as dual-action candidates. This dual engagement suggests that these compounds may contribute to the suppression of inflammatory processes while reinforcing chemopreventive defenses, thereby offering promising leads for integrated therapeutic strategies. Cirsimaritin binds favorably within the active sites of AKR1C3 and PARP1, forming multiple stabilizing hydrogen-bond, π- π stacking, and hydrophobic interactions with key residues in each binding pocket. The docked conformations illustrated in Figs. 12 and 13 were subsequently used as starting structures for molecular dynamics simulations to assess the stability of these interactions under dynamic conditions.

**Table 2:** Docking scores of the top predicted compounds from *Salvia* species against hub-gene targets involved in KEAP1–NRF2-related chemoprevention.

No.	Compound name	Binding energies (kcal/mol)				
		EGFR (PDB 1M17)	PPARG (PDB 2HFP)	AKR1C1 (PDB 3C3U)	SRC (PDB 1A09)	AKR1C3 (PDB 1RY0)
1	Luteolin *O*-glucuronide	−8.6 ± 0.0	−7.4 ± 0.1	−8.1 ± 0.1	−7.9 ± 0.0	−8.9 ± 0.0
2	Hispidulin 7-glucoside	−8.5 ± 0.0	−7.0 ± 0.1	−7.5 ± 0.0	−7.3 ± 0.1	−10.0 ± 0.0
3	Acacetin	−8.7 ± 0.0	−6.5 ± 0.0	−9.9 ± 0.1	−7.0 ± 0.1	−9.4 ± 0.1
4	Diosmetin	−8.9 ± 0.0	−8.0 ± 0.0	−8.6 ± 0.0	−6.5 ± 0.1	−9.2 ± 0.0
5	Apigenin	−8.2 ± 0.0	−6.2 ± 0.2	−7.3 ± 0.1	−6.4 ± 0.0	−9.0 ± 0.2
6	Apigenin 7-*O*-glucoside (Apigetrin)	−8.9 ± 0.0	−8.0 ± 0.0	−9.7 ± 0.0	−7.2 ± 0.0	−9.1 ± 0.0
7	Apigenin dimethyl ether (Apigenin 7,4’-dimethyl ether)	−8.5 ± 0.0	−7.1 ± 0.0	−9.1 ± 0.0	−6.5 ± 0.1	−8.3 ± 0.0
8	Salvigenin	−8.2 ± 0.0	−8.2 ± 0.0	−10.1 ± 0.0	−6.6 ± 0.0	−9.0 ± 0.0
9	Cirsimaritin	−9.8 ± 0.0	−7.3 ± 0.0	−8.4 ± 0.0	−7.9 ± 0.0	−10.9 ± 0.0
10	Velutin	−8.5 ± 0.0	−6.6 ± 0.0	−9.9 ± 0.0	−6.7 ± 0.1	−9.3 ± 0.1
11	4’-bromoflavone (Positive control)	−8.7 ± 0.2	−5.7 ± 0.0	−9.9 ± 0.1	−5.9 ± 0.0	−6.3 ± 0.1

**Table 3:** Docking scores of the top predicted compounds from *Salvia* species against iNOS-related hub targets involved in anti-inflammatory activity.

No.	Compound name	Binding energies (kcal/mol)				
		TNF (PDB 7KBA)	SRC (PDB 1A09)	CASP3 (PDB 1RHU)	CASP8 (PDB 1F9E)	PARP1 (PDB 2RCW)
1	Luteolin *O*-glucuronide	−8.2 ± 0.2	−7.9 ± 0.0	−7.8 ± 0.0	−8.1 ± 0.0	−7.3 ± 0.0
2	Hispidulin 7-glucoside	−7.2 ± 0.0	−7.3 ± 0.0	−7.2 ± 0.1	−8.2 ± 0.1	−9.6 ± 0.0
3	Acacetin	−7.9 ± 0.0	−7.0 ± 0.1	−7.1 ± 0.1	−8.1 ± 0.0	−7.9 ± 0.0
4	Diosmetin	−6.9 ± 0.1	−6.5 ± 0.1	−7.0 ± 0.0	−7.6 ± 0.0	−7.2 ± 0.1
5	Apigenin	−6.3 ± 0.2	−6.4 ± 0.1	−6.9 ± 0.1	−7.3 ± 0.1	−6.8 ± 0.0
6	Apigenin 7-*O*-glucoside (Apigetrin)	−10.0 ± 0.0	−7.2 ± 0.0	−7.0 ± 0.0	−8.0 ± 0.0	−8.8 ± 0.1
7	Apigenin dimethyl ether (Apigenin 7,4’-dimethyl ether)	−6.2 ± 0.1	−6.5 ± 0.1	−7.3 ± 0.1	−6.8 ± 0.2	−7.2 ± 0.1
8	Salvigenin	−7.9 ± 0.1	−6.6 ± 0.0	−6.9 ± 0.0	−7.7 ± 0.0	−8.4 ± 0.1
9	Cirsimaritin	−9.0 ± 0.0	−7.9 ± 0.0	−8.5 ± 0.0	−9.5 ± 0.0	−10.7 ± 0.0
10	Velutin	−8.5 ± 0.1	−6.7 ± 0.0	−7.2 ± 0.0	−7.9 ± 0.2	−7.1 ± 0.0
11	N-(3-(aminomethyl)benzyl)acetamidine (Positive control)	−7.6 ± 0.0	−5.3 ± 0.1	−5.7-±0.1	−6.7 ± 0.0	−6.7 ± 0.2

### RMSD analysis

**Fig. 14:**
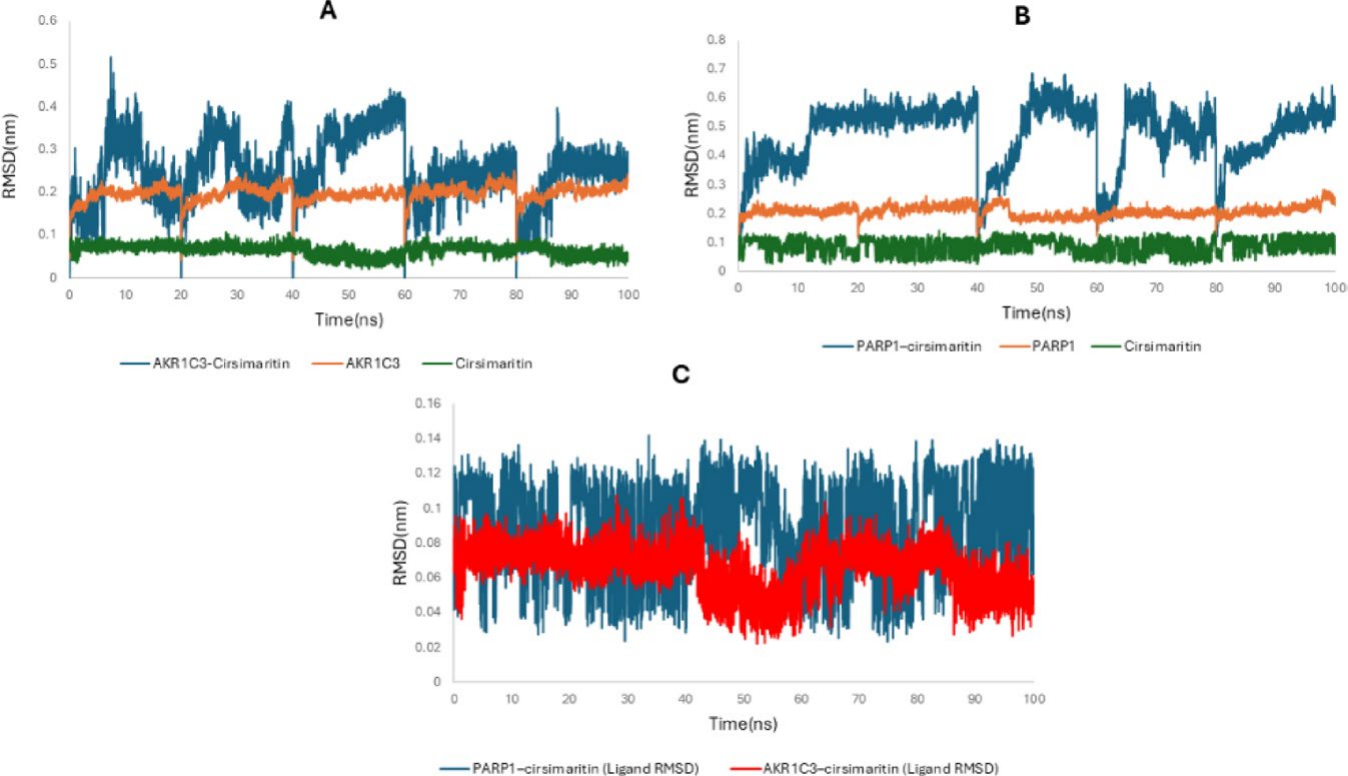
RMSD analysis of cirsimaritin binding to AKR1C3 and PARP1. **(A)** RMSD of AKR1C3–cirsimaritin complex compared to apo-AKR1C3 and free cirsimaritin. **(B)** RMSD of PARP1–cirsimaritin complex compared to apo-PARP1 and free cirsimaritin. **(C)** Ligand RMSD comparison revealing distinct stability profiles of cirsimaritin in AKR1C3 and PARP1.

**Fig. 15:**
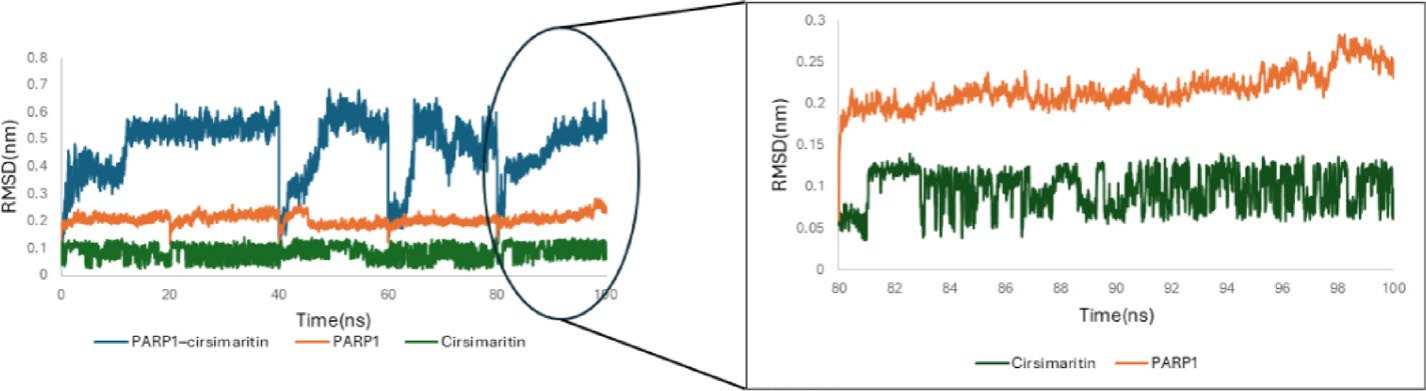
RMSD analysis of cirsimaritin binding to PARP1. **(A)** RMSD of the PARP1–cirsimaritin complex compared to apo-PARP1 and free cirsimaritin. **(B)** Enlarged view of the 80–100 ns segment, highlighting the most equilibrated phase of PARP1 and cirsimaritin dynamics.

MD simulation was conducted to evaluate the stability of compound cirsimaritin with both the AKR1C3 complex and the PARP1 complex. The AKR1C3–cirsimaritin complex displayed higher cirsimaritin mobility (2.22 Å ± 0.23), suggesting bound but flexible behavior of cirsimaritin within the pocket **(**Fig. 14C**).** The protein RMSD was relatively stable (1.33 Å ± 0.16), reflecting a well-preserved backbone. The overall complex RMSD (1.96 Å ± 0.18) supports the interpretation of a stable system with localized cirsimaritin fluctuations (Fig. 14A). In contrast, for the PARP1–cirsimaritin complex, the cirsimaritin RMSD across the entire trajectory was relatively high (2.51 Å ± 0.73), reflecting the dynamic nature of PARP1 and the cirsimaritin’s adaptability within the binding pocket^86^**(**Fig. 14B**)**. The protein backbone remained stable (1.52 Å ± 0.23), while the overall complex RMSD was moderate (2.47 Å ± 1.46). To account for PARP1’s intrinsic flexibility, RMSD values were also calculated using the equilibrated segment of the trajectory 80–100 ns **(**Fig. 15**)**. Enlarged view of the 80–100 ns segment, highlighting the most equilibrated phase of PARP1 and cirsimaritin dynamics. In this figure, the cirsimaritin RMSD was markedly reduced (0.99 Å ± 0.22), indicating tighter binding stability, whereas the protein RMSD remained moderate (1.605 Å ± 0.22). This segment-based analysis highlights that initial fluctuations give way to a more stable binding mode once the system equilibrates^87^. These findings suggest that cirsimaritin remains stably associated in both PARP1 and AKR1C3, with different flexibility profiles. AKR1C3–cirsimaritin complex shows a balance of stable protein conformation and moderate ligand adaptability, whereas PARP1–cirsimaritin demonstrates stabilized binding after equilibration. PARP1 possesses an intrinsically flexible catalytic pocket that undergoes allosteric rearrangements upon ligand binding, enabling sampling of multiple conformational states rather than a rigid binding mode^88,89^. This inherent pocket flexibility explains the increased ligand mobility observed during MD simulations, while still allowing stabilized binding after equilibration, a behavior commonly reported for PARP1–inhibitor complexes^90^.

### RMSF analysis

**Fig. 16:**
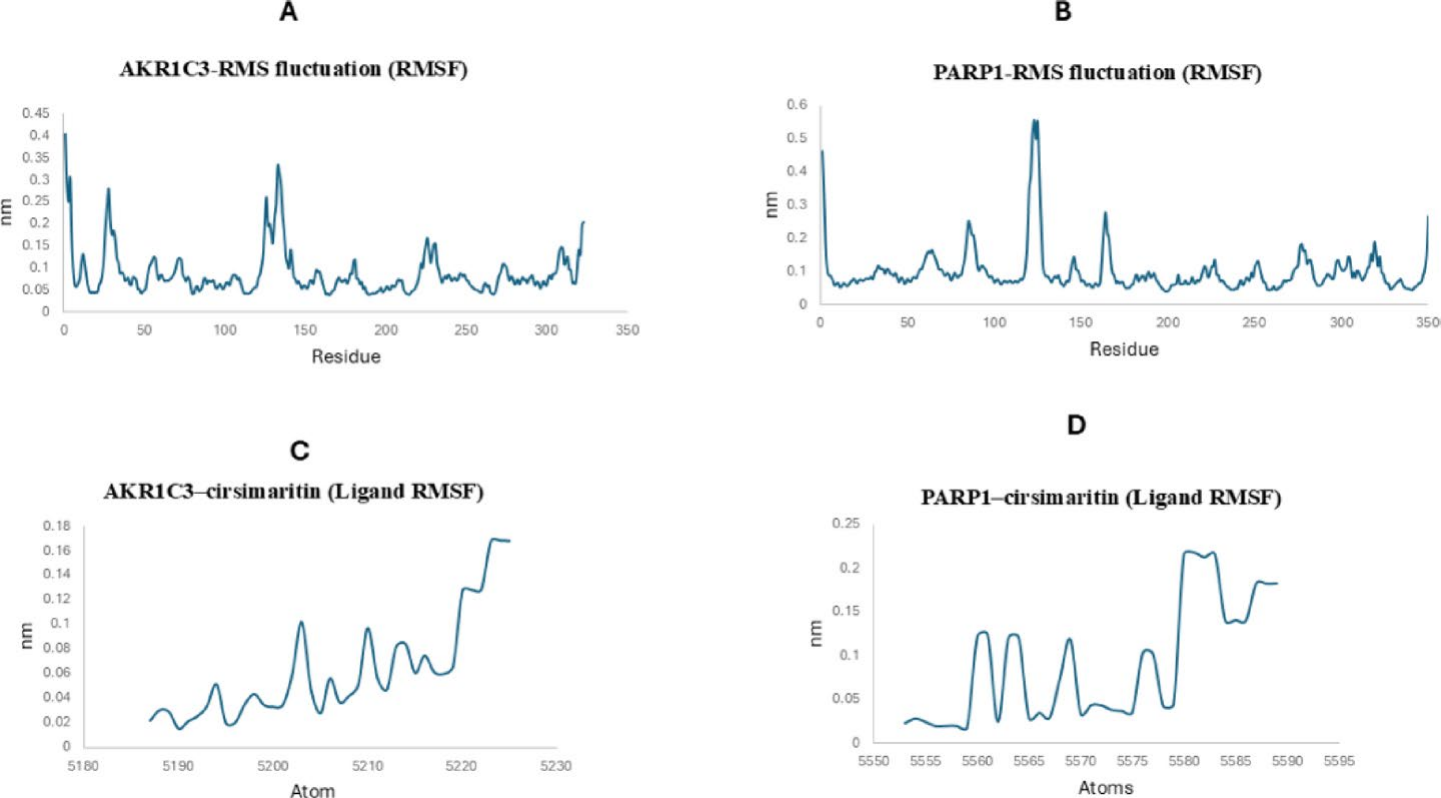
RMSF analysis of AKR1C3–cirsimaritin and PARP1–cirsimaritin complexes over 100 ns. **(A)** Protein (backbone) RMSF of AKR1C3. **(B)** Protein (backbone) RMSF of PARP1. **(C)** Ligand (cirsimaritin) RMSF in the AKR1C3 complex. **(D)** Ligand (cirsimaritin) RMSF in the PARP1 complex.

RMSF measures the flexibility and average positional change of each atom of biomolecules from its mean location over time and is routinely used in MD-based analysis^91^. Average RMSF for each heavy atom was calculated to analyze the flexibility of cirsimaritin in a complex with AKR1C3 and PARP1 **(**Fig. 16**)**. For the AKR1C3–cirsimaritin complex, the protein RMSF averaged 1.18 Å ± 0.65 **(**Fig. 16A**)**, with fluctuations primarily localized to loops and termini, consistent with expected structural flexibility. The ligand RMSF was low overall (0.48 Å for whole-ligand/single-residue and 0.63 ± 0.43 Å atom-wise; Fig. 16C), indicating that most ligand atoms remained stably positioned with only minor flexibility in specific groups. These results indicate that most ligand atoms remain stably positioned, with only minor flexibility in certain groups. For the PARP1–cirsimaritin complex, RMSF analysis across the full trajectory revealed protein fluctuations of 1.17 Å ± 0.67 (Fig. 16B), reflecting moderate backbone flexibility. The ligand RMSF values were 0.71 Å (whole ligand) and 0.91 ± 0.68 Å (atom-wise; Fig. 16D), suggesting overall stable binding with localized flexibility. These results confirm that cirsimaritin maintains stable binding after equilibration, with fluctuations largely confined to flexible side chains and solvent-exposed groups.

### Hydrogen bond analysis

**Fig. 17:**
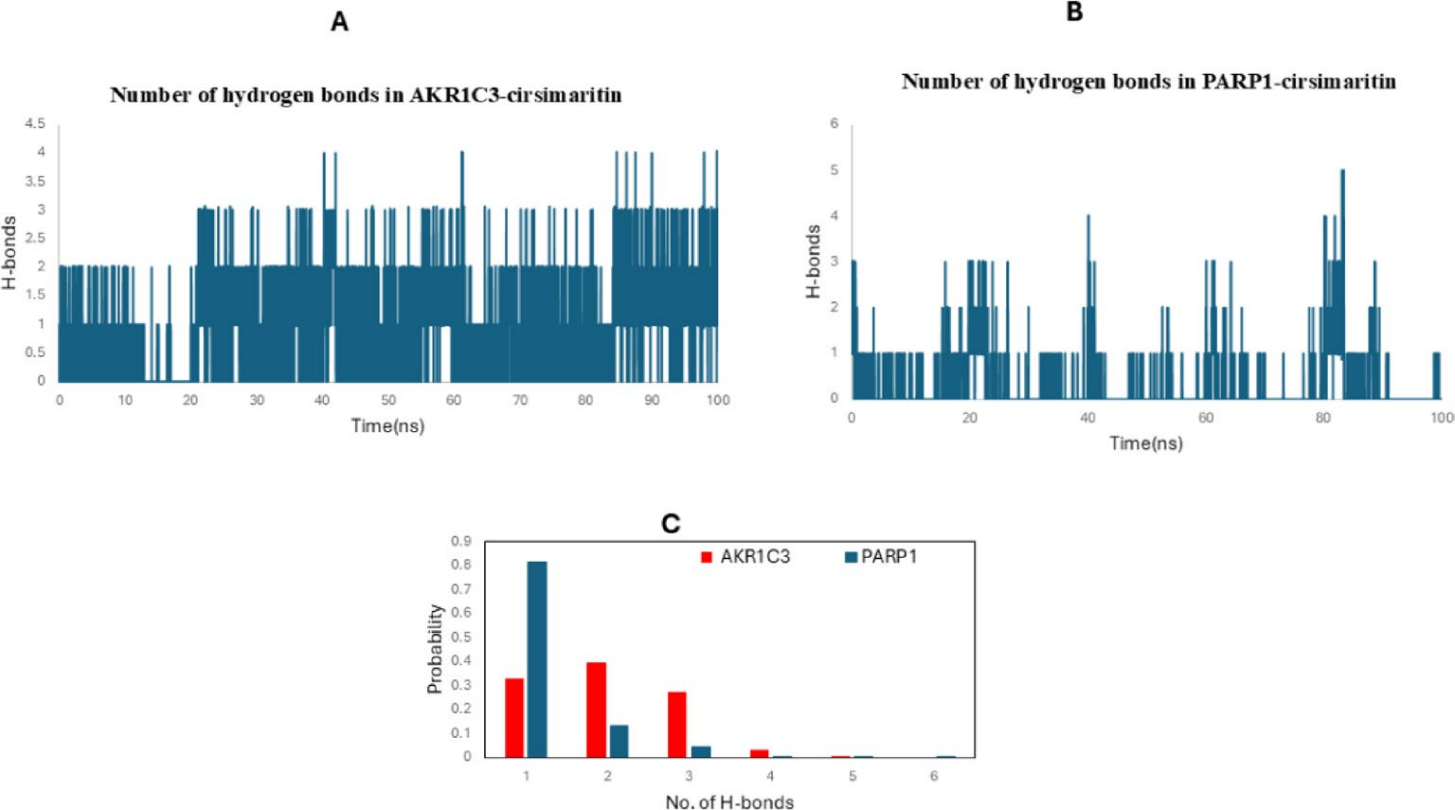
**(A–C)** Hydrogen bond analysis of cirsimaritin with AKR1C3 and PARP1. **(A)** Number of hydrogen bonds formed in the AKR1C3–cirsimaritin complex across 100 ns. **(B)** Number of hydrogen bonds formed in the PARP1–cirsimaritin complex across 100 ns. **(C)** Probability distribution of hydrogen bond counts, indicating more persistent hydrogen bonding in the AKR1C3 complex, whereas the PARP1 complex exhibits more fluctuating hydrogen bond formation, consistent with a dynamic binding mode.

Hydrogen bonds are an important factor of ligand stability and interaction strength during MD simulations^92^. For the AKR1C3–cirsimaritin complex, cirsimaritin formed an average of 1.20 ± 0.81 hydrogen bonds, in agreement with stable binding and moderate interaction strength. The Gaussian distribution analysis confirmed that hydrogen bond formation was frequent, with probabilities favoring one to two bonds across the equilibrated trajectory^93^. This suggest that cirsimaritin maintains persistent polar contacts with AKR1C3, increasing its binding stability. In contrast, the PARP1–cirsimaritin complex demonstrated fewer hydrogen bonds, with an average of 0.24 ± 0.59. This lower frequency reflects the dynamic nature of PARP1 and the reduced reliance on hydrogen bonding for cirsimaritin stabilization. Instead, cirsimaritin binding in PARP1 appears to be supported by hydrophobic and van der Waals interactions, compatible with the RMSD and RMSF analyses that point out PARP1’s conformational flexibility. This observed flexibility is consistent with the intrinsically flexible binding pocket of PARP1^9^. Summarily, these hydrogen bond results complement the RMSD and RMSF findings. AKR1C3–cirsimaritin demonstrates stronger polar interactions, while PARP1–cirsimaritin relies more on non-polar contacts within a dynamic binding environment. Figure 17 showed hydrogen bond analysis of cirsimaritin with AKR1C3 and PARP1.

### Hydrogen bond and contact occupancy analysis

The molecular dynamics trajectories exhibited distinct binding behaviors for both the AKR1C3–cirsimaritin and PARP1– cirsimaritin complexes. For the AKR1C3–cirsimaritin complex, cirsimaritin preserved an average of 2–3 hydrogen bonds during the stable phase (20–100 ns), with an overall hydrogen bond occupancy of 67.99% and contact occupancy of 75%. The average center-of-mass distance was 0.823 ± 0.085 nm, indicating a stable complex. Residue-level mapping showed conserved interactions with Gly22, Asp50, Tyr24, Ser217, Ser221, Gln222, Lys270, and Trp227. While both Leu268 and Tyr216 lost overtime as transient contacts. New interactions with Tyr55 and Phe306 developed. The binding site occupancy was approximately 68.8%, reflecting the stability and position adaptation. Although the trajectory-wide average hydrogen bond count was 1.20, the equilibrated phase (20–100 ns) exhibited recurrent transient formation of 2–3 hydrogen bonds, indicating enhanced interaction stability after equilibration. While for PARP1-cirsimaritin complex, cirsimaritin exhibited a more dynamic binding active site. The COM separation was 1.52 ± 0.78 nm, reflecting fluctuations. Hydrogen bond occupancy was lower (25.24%), and contact occupancy reached 75%, indicating sustained ligand association. Residue-level analysis showed contributions from Tyr328, Lys288, and Pro399, with the emergence of a hydrogen bond involving Thr294 (1.65% occupancy), consistent with a flexible and adaptive PARP1 binding pocket. The observed fluctuations in COM distance and reduced hydrogen bond occupancy for PARP1 are aligned with its naturally dynamic binding pocket^[94,95^ (Supplementary figure S5).

### The radius of gyration analysis

**Fig. 18:**
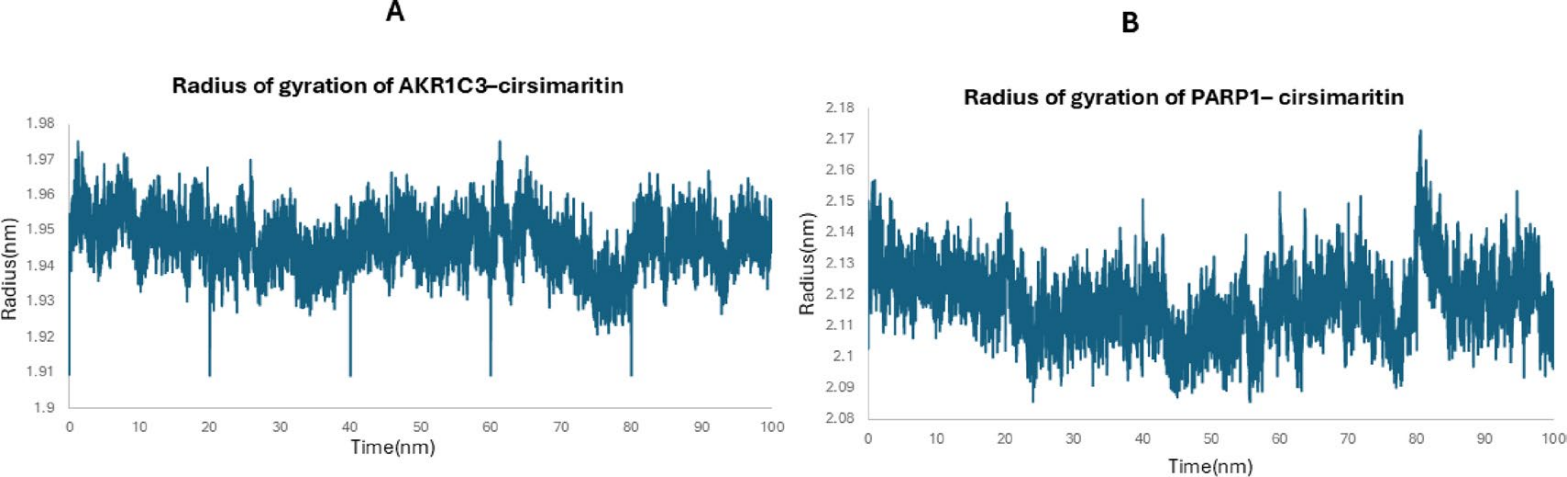
**(A–B)** Radius of gyration (Rg) analysis of cirsimaritin-bound complexes. **(A)** Rg of AKR1C3–cirsimaritin across 100 ns, showing moderate structural fluctuations. **(B)** Rg of PARP1–cirsimaritin, indicating greater compactness and reduced flexibility compared to AKR1C3.

The radius of gyration (Rg) was computed to evaluate the compactness and global structural stability of the protein–ligand complexes during MD simulations^96^. Rg is a frequently utilized descriptor that demonstrates the spatial distribution of atoms around the molecular center of mass and highlights conformational stability during simulations^97^. For the AKR1C3–cirsimaritin complex, the protein Rg averaged 1.946 ± 0.007 nm, remaining stable throughout the simulation with only minor fluctuations. This stability indicates that the protein maintained a compact and stable conformation. For the PARP1–cirsimaritin complex, the protein Rg values fell within the expected range of 1.7–2.1 nm ± 0.01, with only small deviations observed across the trajectory. These results confirm that PARP1 retained its compactness despite its dynamic nature, and cirsimaritin binding did not induce significant structural expansion or collapse. Overall, both complexes exhibited stable Rg profiles, with fluctuations confined to narrow ranges. This suggest that cirsimaritin binding does not destabilize the global protein fold in either target. Detailed Rg plots are provided in Fig. 18.

### The solvent accessible surface area analysis

**Fig. 19:**
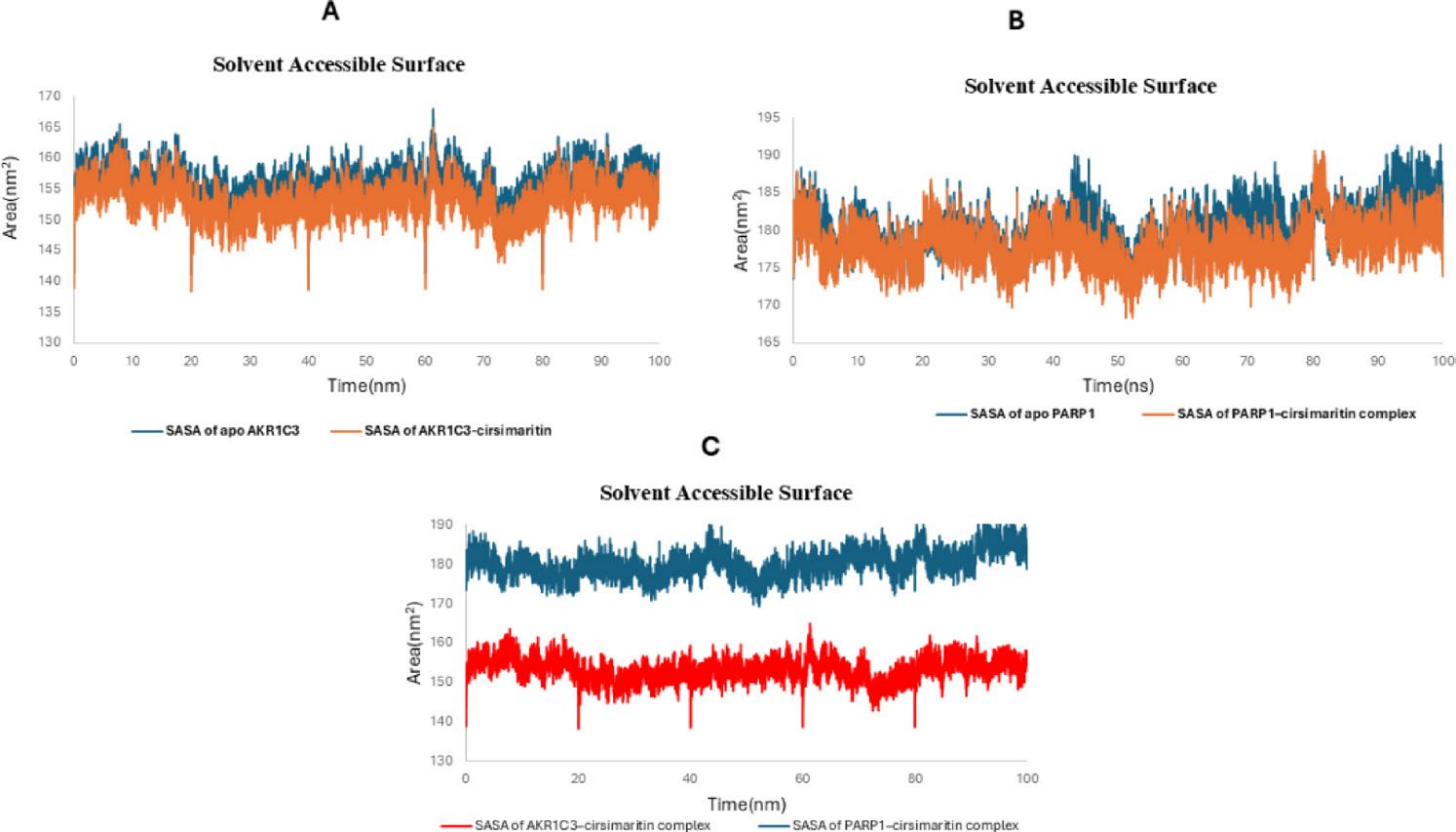
**(A–C)** Solvent accessible surface area (SASA) analysis of apo proteins and ligand complexes. **(A)** SASA of apo AKR1C3 compared with the AKR1C3–cirsimaritin complex, showing reduced solvent exposure upon ligand binding. **(B)** SASA of apo PARP1 compared with the PARP1–cirsimaritin complex, highlighting stabilization of the protein core. **(C)** Comparative SASA profiles of AKR1C3–cirsimaritin and PARP1–cirsimaritin complexes, indicating greater surface burial in AKR1C3, consistent with stronger ligand interaction.

The solvent accessible surface area (SASA) was calculated to evaluate the degree of solvent exposure and compactness of the protein–ligand complexes during MD simulations^98^. SASA is extensively applied parameter that indicates the extent to which residues and ligands are exposed to solvents thus offering insights into binding stability and conformational dynamics^9^. For the apo AKR1C3, the average SASA was 155.4 ± 2.70 nm², indicating a compact and structurally stable protein surface throughout the simulation. In comparison, the apo PARP1 exhibited a higher average SASA (178.36 ± 2.93 nm²), consistent with its inherently flexible structural nature. The SASA of free cirsimaritin averaged 5.40 ± 0.19 nm², reflecting its solvent-exposed state prior to binding. Upon complex formation with AKR1C3, cirsimaritin exhibited reduced SASA values, indicating deep burial within the binding pocket with limited solvent exposure. Similarly, when bound to PARP1, cirsimaritin displayed an average SASA of 5.38 ± 0.21 nm², consistent with stable burial in the active site and only minor fluctuations in solvent accessibility. Comparative analysis of apo and complex protein SASA profiles (Fig. 19) demonstrates that cirsimaritin binding does not induce significant expansion of the protein surface area, indicating preservation of global protein compactness upon ligand association. Detailed SASA profiles are presented in Fig. 19.

### MM-GBSA calculations

**Fig. 20:**
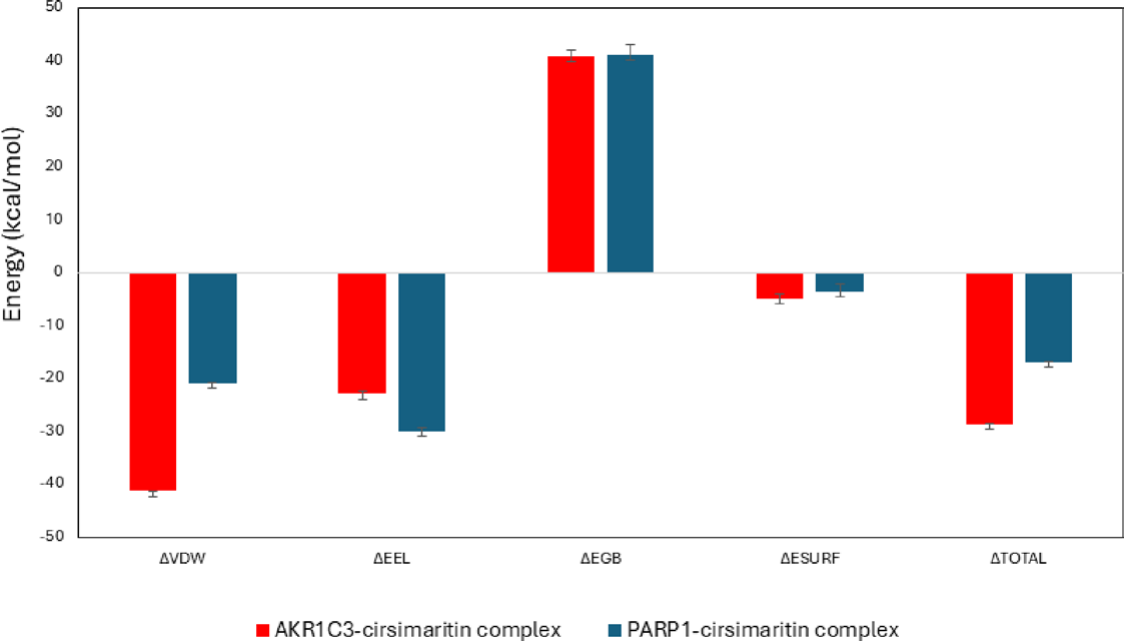
MM-GBSA binding free energy decomposition for cirsimaritin with AKR1C3 (red bars) and PARP1 (blue bars). Bars show contributions from van der Waals (ΔVDW), electrostatic (ΔEEL), polar solvation (ΔEGB), non-polar solvation (ΔESURF), and the overall binding free energy (ΔTOTAL). Error bars indicate the standard deviation between each run of the systems. In both complexes the large negative ΔVDW and ΔEEL favors binding, while positive ΔEGB opposes binding. Error bars represent the standard deviation of the MM-GBSA binding free energies.

MM-GBSA calculations indicated favorable binding of cirsimaritin to AKR1C3 and moderate binding to PARP1 over the 20–100 ns trajectories. For the AKR1C3-cirsimaritin complex, the binding free energy (ΔTOTAL = − 28.7 ± 0.53 kcal/mol) remained consistently negative, reflecting the stability of the cirsimaritin association throughout the simulation. The important stabilizing factor was ΔVDW (− 41 ± 0.43 kcal/mol), the AKR1C3 binding pocket is dominated by hydrophobic interactions, primarily involving residues such as Trp227, Tyr268, Leu268, and Tyr216, which contribute to stabilizing cirsimaritin within the active site. Electrostatic contributions (ΔEEL = − 22.95 ± 1.06 kcal/mol) also improved stability, while polar solvation (ΔEGB = + 40.78 ± 0.91 kcal/mol) partially counteracted these favorable interactions. In contrast, PARP1 exhibited weaker binding (ΔTOTAL = − 16.9 ± 0.79 kcal/mol) with greater variability across the trajectory. The binding stability under equilibrated conditions was also measured, whereas MM-GBSA analysis was done on the final equilibrated segment (80–100 ns). During this period, the binding free energy remained favorable (ΔTOTAL = − 22.63 ± 3.47 kcal/mol), indicating improved stabilization relative to the full-trajectory average. Although van der Waals and electrostatic forces contributed favorably, their magnitudes were lower compared to AKR1C3-cirsimaritin complex. The effect of ΔEGB was also increased, leading to reduced overall binding affinity. These results highlight the AKR1C3-cirsimaritin complex as the stronger binder, with hydrophobic interactions, while PARP1 showed more fluctuating and weaker stabilization. Figure 20 showed the MM-GBSA binding free energy decomposition of cirsimaritin with AKR1C3 and PARP1, illustrating the contributions from van der Waals (ΔVDW), electrostatic (ΔEEL), polar solvation (ΔEGB), nonpolar solvation (ΔESURF), and the overall binding free energy (ΔTOTAL). The comparative presentation highlights cirsimaritin’s dual effect, with stronger stabilization in AKR1C3 and weaker but measurable binding to PARP1. The numerical values underlying this Fig. are provided in Supplementary Table S6.

### Induction of chemopreventive marker NQO1

**Fig. 21:**
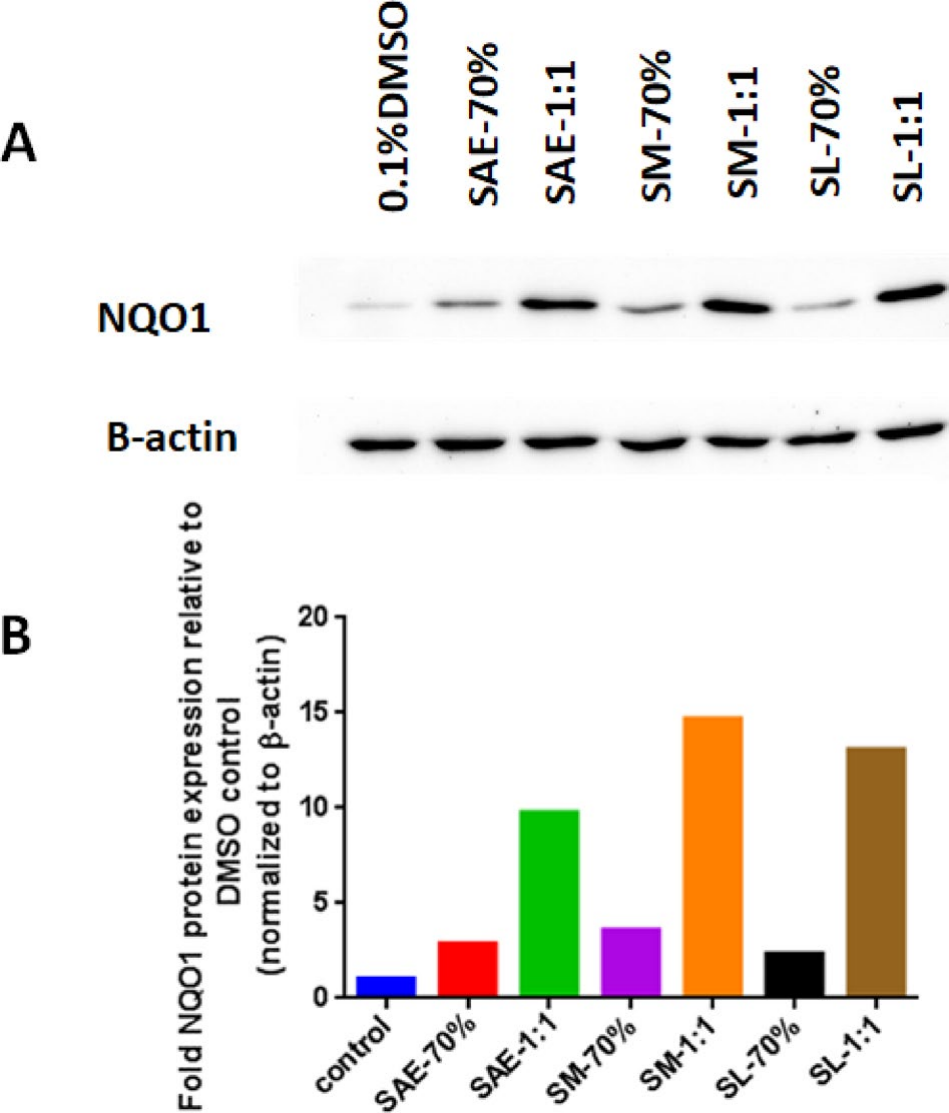
Western blot showing Upregulation of the chemopreventive marker NQO1 by vehicle (control, 0.1%DMSO, v/v) or 100 µg/mL of methylene chloride: methanol (1:1) or 70% methanol extracts of the studied *Salvia* species in Hepa1c1c7 cells **(A)**. Densitometric analysis of NQO1 protein bands was shown as fold change to control, normalized to corresponding β-actin protein bands **(B)**. Uncropped version of this blot is displayed in the supplementary information file as Supplementary Fig. S6.

The biological evaluation of six extracts from the three *Salvia* species was commenced by employing the murine hepatoma (Hepa1-c1c7) chemoprevention model to test for the induction of the chemopreventive marker NQO1. As displayed in Fig. 21A&B, Western blotting analysis of NQO1 was employed. As displayed in Fig. 21, methylene chloride: methanol (1:1) extracts of *S. aegyptiaca*, (SAE), and *S. lanigera* (SL) caused a moderate NQO1 protein induction. As shown, *S. multicaulis* (SM) showed the potency to induce the protein extression of the chemopreventive marker NQO1, recording the highest expression among other tested extracts Fig. 21A, B.

To the best of our knowledge, no previous reports exist about the chemopreventive NQO1 inducer activities of *Salvia multicailis.* The activity of NQO1 induction by SM 1:1 can be largely attributed to their chemical constituents of the UPLC-HRMS/MS where the resulting compounds shown in the *in silico* study to have high inhibitory potential to the KEAP1-Nrf2 complex which is known to cause induction of its down-stream protein, NQO1^11–13^.

### The inhibitory effect of *Salvia* species on NO production

The anti-inflammatory properties of three *Salvia* species extracts were evaluated using the RAW264.7 murine macrophage cell line model. The pre- screening of anti-inflammatory activity was performed on methylene chloride: methanol (1:1) and 70% methanol extracts of *Salvia* species as inhibitors of LPS-induced NO release in RAW 264.7 cells. In this rapid screening strategy of the *Salvia* species extracts, a single dose of 100 µg/mL is used. The 100 µg/mL dose is considered a selected high one for an *in vitro* study that permits feasible extrapolation into any future possible *in vivo* experiments on experimental animals. Regarding the single dose for prescreening, it is a common practice in the literature involving testing. For example, the National Cancer Institute of the NIH has long been using this screening strategy for testing medicinal plant extracts^99^.

**Fig. 22:**
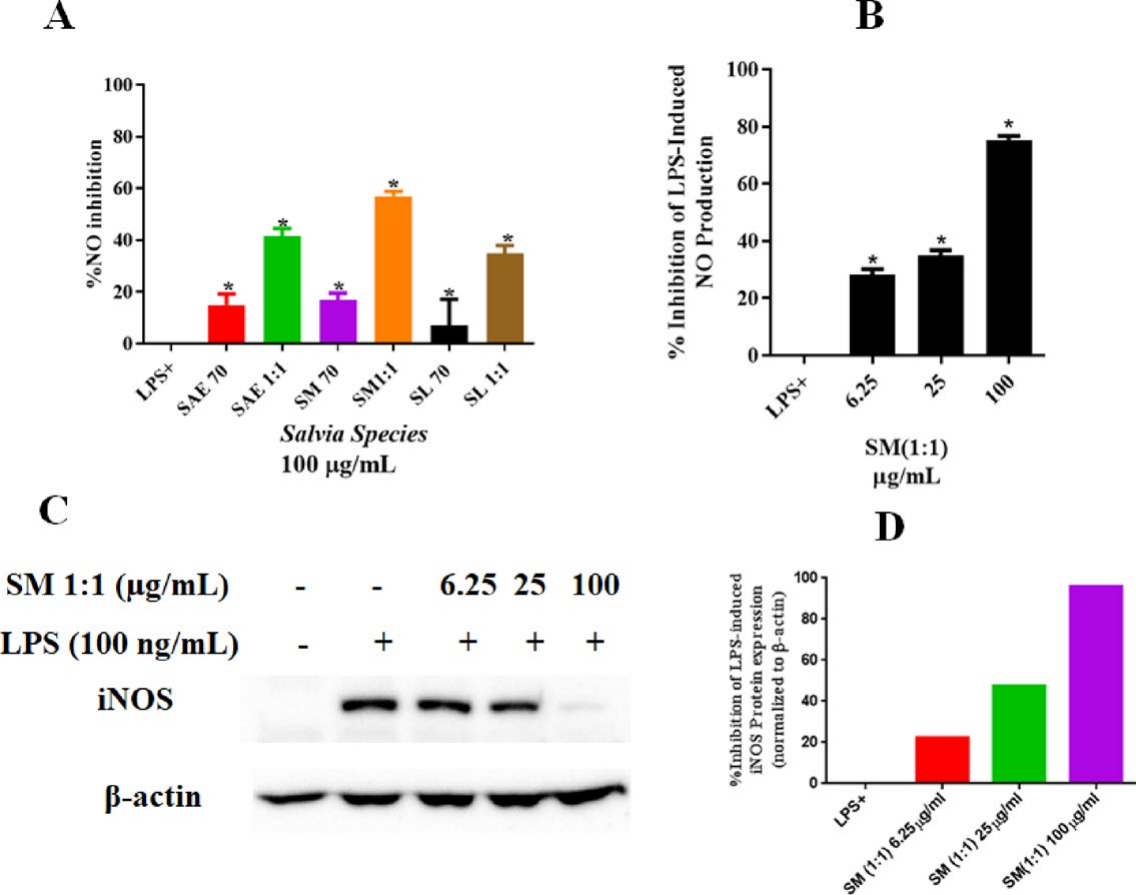
**(A)** Preliminary screening of methylene chloride: methanol (1:1) and 70% methanol extracts of the studies *Salvia* as inhibitors of LPS-induced NO release in RAW 264.7 cells (* denotes significantly different than LPS+ (*P* < 0.05, one way ANOVA, *n* = 8 replica), **(B**) Inhibitory effect of *S. multicaulis* 1:1 at different concentrations on Nitric Oxide (NO). Data are means ± SEM. *denotes significant difference compared to LPS^+^, *P* < 0.05, one-way ANOVA, *n* = 2 replica, performed on GraphPad Prism V8 (San Diego, USA). **(C)** Assessment of the anti-inflammatory potential by Western blot analysis of iNOS expression with or without the lipopolysaccharide activator (LPS) in RAW 264.7 macrophages for *S. multicaulis* 1:1 at different concentrations. LPS^+^:cells treated with LPS only (i.e., to represent zero NO %inhibition and zero iNOS %inhibition of protein expression). All other experimental groups represent cells that are treated with indicated concentrations of the extracts in the presence of LPS. (**D**) Densitometric analysis of iNOS bands normalized to their corresponding β-actin and represented as % inhibition of normalized LPS^+^ iNOS band. Original, uncropped, horizontally unflipped version of (C) is displayed in the supplementary information file as Supplementary Figure S7.

As displayed in Fig. 22A, the preliminary screening revealed the potency of methylene chloride: methanol (1:1) of *S. multicaulis* (SM 1:1), which inhibited LPS-induced NO release in RAW 264.7 cells, recording an inhibition of 56.9% ±0.6 compared to LPS -treated macrophages. Further concentration- dependent inhibition was performed, as displayed in Fig. 22B, and revealed a concentration-dependent effect.

Western blotting was then employed to assess the inhibition of iNOS protein expression by methylene chloride: methanol (1:1) of *S. multicaulis* in RAW264.7 cell lysates. As displayed in Fig. 22C, S. *multicaulis* (SM 1:1) inhibited the LPS-induced protein expression of iNOS, with maximum inhibition at 100 µg/mL. The experimental findings of the potential of SM1:1 to inhibit LPS inflammation can be partially explained by the *in silico* study that showed the direct inhibitory effects of its compound constituents on iNOS enzyme at the catalytic level. Additionally, the displayed activity shown by SM 1:1 to inhibit LPS-induced iNOS protein expression by western blotting can also be examined *in silico*, employing iNOS as a target^100^.

The selection of the prescreening and testing non-toxic doses of the extracts for both the chemoprevention and anti-inflammatory experiments depended on routine microscopic morphological assessment of cultured cells, treated or untreated. However, this comprises a limitation for the *in vitro* study where the parallel testing of cell viability could have a more quantitative measure of the non-toxicity of these extracts to cells.

## Conclusion

The secondary metabolome of three *Salvia* species cultivated in Egypt, *S. aegyptiaca*,* S. multicaulis*, and *S. lanigera*, was profiled using UPLC-HRMS/MS, in combination with FBMN through the GNPS platform. In total, 218 metabolites were dereplicated of various chemical classes such as phenolic acids (benzoic and cinnamic acid derivatives), flavonoid *O*-/*C***-** glycosides, lignans, terpenoids (i.e., monoterpene, diterpene, sesquiterpenoid, megastigmanes, and triterpenes), and other miscellaneous compounds. Full structure elucidation of these metabolites using other spectroscopic techniques, i.e., NMR post-isolation, should follow.

The current study has effectively highlighted the significance of *S. multicaulis* as a promising reservoir of chemopreventive and anti-inflammatory attributes. Cirsimaritin demonstrates a primary binding effect toward AKR1C3, represented by rigid pocket stabilizing, conserved hydrogen bonds, and favorable binding stability throughout the simulations. In contrast, its interaction with PARP1 represents adaptive binding dynamics and solvation-sensitive stabilization, supporting cirsimaritin’s role as a selective AKR1C3 ligand with secondary PARP1 modulation. These compounds were primarily detected in *S. multicaulis*, indicating this species as a potential natural source for multifunctional bioactive agents.

Finally, this study offers an in-depth overview of the phytochemical and pharmacological characteristics of the *Salvia* species, highlighting current knowledge gaps and suggesting directions for future research. This aims to support the broader therapeutic use of *Salvia* and its potential role in pharmaceutical development. These findings could contribute to the development of natural, plant-based interventions for the prevention of cancer and other inflammation-related diseases.

## Supplementary Information

Below is the link to the electronic supplementary material.

Supplementary Material 1

## Data Availability

All data generated or analyzed during this study are included in this published article [and its supplementary information file].

## Abbreviations

RMSD: Root Mean Square Deviation
Rg: Radius of Gyration
SASA: Solvent Accessible Surface Area
RMSF: Root Mean Square Fluctuation
COM: Center of Mass
MD: Molecular Dynamics
MM-GBSA: Molecular Mechanics- Generalized Born Surface Area
Gly22: Glycine at position 22
Leu268: Leucine at position 268
Tyr24: Tyrosine at position 24
Tyr55: Tyrosine at position 55
Tyr216: Tyrosine at position 216
Tyr328: Tyrosine at position 328
Phe306: Phenylalanine at position 306
Trp227: Tryptophan at position 227
Ser217: Serine at position 217
Ser221: Serine at position 221
Thr294: Threonine at position 294
Gln222: Glutamine at position 222
Lys270: Lysine at position 270
Lys288: Lysine at position 288
Arg197: Arginine at position 197
Asp50: Aspartic acid at position 50
Asp238: Aspartic acid at position 238
Pro399: Proline at position 399
