# Supplementary material for: Comprehensive metabolomic profiling of Egyptian Salvia species reveals promising leads for chemopreventive and anti-inflammatory drug development: In vitro and In silico study
Source: Sci Rep. 2026 Jun 9;16:17846. doi: 10.1038/s41598-026-55968-x (PMC13249958; doi:10.1038/s41598-026-55968-x)
Supplement: Supplementary file 1 — Supplementary Material 1 [file 41598_2026_55968_MOESM1_ESM.docx]

**Supplementary Data**

**Comprehensive Metabolomic Profiling of Egyptian *Salvia* Species Reveals Promising Leads for Chemopreventive and Anti-Inflammatory Drug Development: *In Vitro* and *In silico* Study**

Ahmed R. Hamed^a*^, Sherin K. Ali^a^, Sally A. Abdel-Halim^a^, Shaymaa M. Bata^a^,Nesrine M. Hegazi^b^, Tarik A. Mohamed^a^, Mohamed-Elamir F. Hegazy^a*^

^a^Chemistry of Medicinal Plants Department, National Research Centre, 33 El-Bohouth St., Dokki, Giza 12622, Egypt; nlragab2004@yahoo.com (A.R.H.); tarik.nrc83@yahoo.com (T.A.M.); sheryali57@yahoo.com (S.K.A).

^b^Phytochemistry and Plant Systematics Department, National Research Centre, 33 El-Bohouth St., Dokki, Giza 12622, Egypt; nm.hegazi@nrc.sci.eg (N.H.)

Correspondences; [nlragab2004@yahoo.com](mailto:nlragab2004@yahoo.com) (A.R.H.); [mohegazy@uni-mainz.de](mailto:mohegazy@uni-mainz.de) (M.-E.F.H.)

**Supplementary Table**

**Supplementary Table S1:** Annotated metabolites of selected *Salvia species* as revealed by UPLC-HRMS/MS analysis in both ionization modes.

*S. aegyptiaca* L., **( Sal-EG)** , *S. multicaulis* (**SM** ) and *S. lanigera* (**Sal-L**) +: present, ++: abundant, +++: major, -: absent

|  | **Compound Class** | **Compound name** | **Rt**  **(min)** | **Molecular**  **formula** | **Negative mode** | | **Positive mode** | | **Sal-EG** | | **SM** | | **Sal-L** | | **Reference** |
| --- | --- | --- | --- | --- | --- | --- | --- | --- | --- | --- | --- | --- | --- | --- | --- |
|  |  |  |  |  | **[M - H]^-^**  **(error in ppm)** | **MS^2^** | **[M + H]^+^**  **(error in ppm)** | **MS^2^** | **7:30** | **1:1** | **7:30** | **1:1** | **7:3** | **1:1** |  |
|  | **Phenolic acids** | | | | | | | | | | | | | | |
| 1 | Benzenoid | **Hydroxy**  **benzaldehyde** | 5.69 | C_7_H_6_O_2_ | 121.0293  (0.07) | 51.0233,  65.0391, 93.0327, 121.0293 |  |  | ++ | ++ | + | - | - | - | (Kuo, Lee, and Lai 2000) |
| 2 | Cinnamic acid derivative | **Ferulic acid** | 8.23 | C_10_H_10_O_4_ | 193.0504  (1.35) | 105.0343, 133.0292 |  |  | - | + | - | - | - | - | (Karioti et al. 2007) |
| 3 | Cinnamic acid derivative | ***O*- feruloyl quinic acid** | 8.60 | C_17_H_20_O_9_ | 367.1033  (0.6) | 93.0343, 111.0447, 134.0371, 173.0454 |  |  | - | - | + | - | - | - | (Regos, Urbanella, and Treutter 2009) |
| 4 | Cinnamic acid derivative | **Caffeic acid #** | 9.60 | C_9_H_8_O_4_ | 179.035  (-0.47) | 65.0029.  79.0546.  89.0389, 134.0371 |  |  | - | ++ | - | + | - | ++ | (J. Zhao et al. 2011)  (Tian-Xiu and Lian-Niang 1992) |
| 5 | Cinnamic acid derivative | **Rosmarinic acid #** | 9.86 | C_18_H_16_O_8_ | 359.0771  (0.4) | 359.0772 |  |  | - | - | - | + | - | - | (L.-M. Zhao, Liang, and Li 1996)  (J. Zhao et al. 2011) |
| 6 | Cinnamic acid derivative | **Hydroxy-allylbenzene-*O*-hexoside** | 12.70 | C_15_H_20_O_7_ | 311.1135  (0.15) | 122.0369, 133.0289,  148.0523 |  |  | + | - | - | - | - | - | (Gupta et al. 2007) |
| 7 | Cinnamic acid derivative | **Clinopodic acid B** | 13.93 | C_19_H_18_O_8_ | 373.0932  (-1.04) | 134.0370,  161.0242.  179.0348 |  |  | + | + | - | - | - | + | (Murata et al. 2009) |
| 8 | Cinnamic acid derivative | **Salviaflaside #** | 15.09 | C_24_H_26_O_13_ | 521.1296  (0.82) | 109.0288, 152.0114, 191.0342, 205.0505, |  |  | + | - | - | - | - | - | (Tezuka et al. 1998) |
| 9 | Cinnamic acid derivative | **Populoside** | 15.30 | C_22_H_24_O_10_ | 447.1297  (-1.06) | 135.0447, 161.0242, 179.0346 |  |  | - | + | - | - | - | - | (Si et al. 2011) |
| 10 | Cinnamic acid derivative | **Cistanoside D** | 15.65 | C_31_H_40_O_15_ | 651.2287  (1.13) | 113.0239, 160.0163,  175.0401, 193.0505 |  |  | + | + | - | - | - | - | (Şahin, Ezer, and Çalış 2004) |
| 11 | Cinnamic acid derivative | ***O*-Acetylmartyonside** | 15.97 | C_33_H_42_O_16_ | 693.2407  (2.17) | 175.0401,  193.0506 |  |  | - | + | - | - | - | - | (Yamamoto et al. 1993) |
| 12 | Cinnamic acid derivative | **Methyl rosmarinate #** | 16.28 | C_19_H_18_O_8_ | 373.0935  (-1.47) | 135.0450,  161.0241,  179.0350 |  |  | ++ | ++ | + | ++ | + | ++ | (KOHDA et al. 1989) |
|  | **Flavonoids** | | | | | | | | | | | | | | |
| 13 | Flavanone  *O*-glycosides | **Eriodictyo-*O*-hexoside** | 8.74 | C_21_H_22_O_11_ |  |  | 473.1051  [M + Na]^+^  (0.35) | 64.9769,  157.0454,  185.0406,  279.0097,  311.0528,  473.1051 | - | - | + | - | + | - | (Gujer, Magnolato, and Self 1986) |
| 14 | Chalcone glycosides | **Pentahydroxy**  **chalcone *O*-hexoside**  **Isomer 1** | 8.80 | C_21_H_22_O_11_ | 449.1083  (1.45) | 135.0447, 151.0035, 173.0454 |  |  | - | - | + | - | + | - | (Ashurst et al. 1967) |
| 15 | Chalcone glycosides | **Pentahydroxy**  **chalcone *O*-hexoside**  **Isomer 2** | 9.62 | C_21_H_22_O_11_ | 449.1087  (0.74) | 107.0133, 135.0449, 151.0038 |  |  | - | - | + | - | + | - | (Ashurst et al. 1967) |
| 16 | Flavone  *C*-glycosides | **Apigenin di-*C*- hexoside #** | 9.67 | C_27_H_30_O_15_ | 593.1506  (0.24) | 353.0663, 383.0767, 473.1080, 593.1510 |  |  | ++ | + | + | - | + | - | (Lu and Foo, 2000)  (Abdalla et al. 1983) |
| 17 | Flavanone  *O*-glycosides | **Eriodictyol -O-rutinoside** | 11.34 | C_27_H_32_O_15_ | 595.1663  (1.04) | 135.0445, 151.0033, 287.0551,  595.1662 |  |  | + | + | + | - | + | - | (Nakagawa et al. 2006) |
| 18 | Flavanone  *O*-glycosides | **Naringenin -*O*-hexoside** | 11.48 | C_21_H_22_O_10_ |  |  | 457.1103  [M +Na]^+^  (0.22) | 175.0021,  185.0414,  295.0589,  337.0530,  457.1098 | - | - | + | - | + | - | (Ibrahim, El-Senousy, and Hawas 2007) |
| 19 | Flavanone  *O*-glycosides | **Hesperetin-*O*-hexoside** | 11.74 | C_22_H_24_O_11_ | 463.1245  (0.33) | 107.0133,  134.0370,  151.0037, 301.0717 | 487.1209  [M +Na]^+^  (0.62) | 174.9999,  185.0409,  325.0698,  337.0525,  487.1205 | - | - | + | - | + | + | (Choi et al. 1990) |
| 20 | Flavone  *O*-glycosides | **Luteolin -*O*-glucuronide**  **Isomer 1** | 12.52 | C_21_H_18_O_12_ | 461.0725  (0.45) | 59.0134,  85.0289,  285.0406 |  |  | - | - | - | - | - | + | (Lu and Foo, 2000) |
| 21 | Flavone  *O*-glycosides | **Luteolin -*O*-****rutinoside** | 12.86 | C_27_H_30_O_15_ | 593.1504  (1.18) | 285.0402, 327.0502, 593.1508 |  |  | ++ | ++ | + | - | + | - | (Inoue et al. 2002) |
| 22 | Flavone  *O*-glycosides | **Luteolin- *O*-glucuronide#**  **Isomer 2** | 13.07 | C_21_H_18_O_12_ | 461.0729  (-1.82) | 59.0134, 85.0293,  286.0438,  300.0274 |  |  | - | - | - | - | + | - | (Lu and Foo, 2000) |
| 23 | Flavonol  *O*-glycosides | **Quercetin-*O***  **-hexoside #** | 13.28 | C_21_H_20_O_12_ | 463.0882  (0.29) | 151.0035, 255.0298,  271.0243  300.0275 |  |  | + | - | ++ | - | + | - | (Bisio et al. 1999) |
| 24 | Flavone  *O*-glycosides | **Luteolin *O*-glucuronide#**  **Isomer 3** | 13.32 | C_21_H_18_O_12_ | 461.0724  (0.59) | 271.0243, 285.0402,  300.0271 |  |  | - | - | - | - | - | + | (Abdalla et al. 1983) |
| 25 | Flavone  *O*-glycosides | **Apigenin-*O*-hexoside # Isomer 1** | 13.89 | C_21_H_20_O_10_ | 431.0981  (0.18) | 117.0340,  149.0246, 225.0556, 268.0375 |  |  | - | - | ++ | - | ++ | + | (Abdalla et al. 1983) |
| 26 | Flavone  *O*-glycosides | **Apigenin-*O*-hexoside # Isomer 2** | 14.14 | C_21_H_20_O_10_ | 431.0983  (0.03) | 151.0036, 240.0429, 269.0439, 431.0983 |  |  | + | + | ++ | ++ | ++ | ++ | (Flores-Bocanegra et al. 2017) |
| 27 | Flavone  *O*-glycosides | **Luteolin -methyl ether *O*-hexoside** | 14.34 | C_22_H_22_O_11_ | 461.1092  (1.26) | 255.0299, 283.0246, 446.0847 |  |  | - | - | + | + | + | + | (Alipieva et al. 2009) |
| 28 | Flavone  *O*-glycosides | **Luteolin -*O* -glucuronide #**  **Isomer 4** | 14.60 | C_21_H_18_O_12_ |  |  | 463.0859  (2.04) | 287.0543,  301.0706 | - | - | - | - | + | - | (Yinron g Lu and Foo 2000) |
| 29 | Flavonol  *O*-glycosides | **Kaempferol-*O*-hexoside** | 14.60 | C_21_H_20_O_11_ | 447.0930  (0.84) | 227.0349, 255.0298, 284.0324 |  |  | + | + | ++ | + | ++ | + | (Karioti et al. 2007) |
| 30 | Flavone  *O*-glycosides | **Apigenin *O*-glucuronide** | 14.74 | C_21_H_18_O_11_ | 445.0779  (-0.16) | ,227.0348, 255.0300,  269.0454, 284.0326, |  |  | + | - | - | - | + | - | (Abdalla et al. 1983) |
| 31 | Flavone  *O*-glycosides | **Hispidulin-*O*-** **hexoside #** | 14.75 | C_22_H_22_O_11_ | 461.1086  (0.69) | 283.0249, 297.0403 |  |  | - | - | ++ | + | ++ | + | (M. Wang et al. 1998) |
| 32 | Flavanol  *O*-glycosides | **Isorhamnetin -*O*-hexoside** | 14.92 | C_22_H_22_O_12_ | 477.1034  (1.6) | 243.0295, 271.0242,  299.0197, 314.0428 |  |  | - | - | ++ | + | ++ | ++ | (Karioti et al. 2003) |
| 33 | Flavone  *O*-glycosides | **Luteolin -methyl ether *O*-glucuronide**  **Isomer 1** | 14.95 | C_22_H_20_O_12_ | 475.0876  (1.1) | 59.0134, 284.0322, 299.0550, 314.0426 |  |  | - | - | - | - | + | - | (Murata, Miyase, and Yoshizaki 2010) |
| 34 | Flavone  *O*-glycosides | **Apigenin-*O*-hexoside** **#** **Isomer 3** | 15.26 | C_21_H_20_O_10_ | 431.0981  (0.35) | 109.0288, 268.0381, 285.0398,  431.0979 |  |  | - | - | + | - | + | - | (Abdalla et al. 1983) |
| 35 | Flavone  *O*-glycosides | **Luteolin-*O*-hexoside** | 15.56 | C_21_H_20_O_11_ | 447.0927  (1.3) | 135.0442, 151.0026, 285.0397 |  |  | + | - | ++ | ++ | + | + | (Yinron g Lu and Foo 2000) |
| 36 | Flavone  *O*-glycosides | **Luteolin -methyl ether *O*-glucuronide**  **Isomer 2** | 15.81 | C_22_H_20_O_12_ | 475.0883  (-1.43) | 137.0241, 284.0320, 299.0552 |  |  | - | - | - | - | + | - | (Murata, Miyase, and Yoshizaki 2010) |
| 37 | Chalcone | **Dihydroxy-dimethoxy-dimethyl dihydrochalcone** | 16.47 | C_19_H_22_O_5_ | 329.1395  (-0.28) | 191.0713,271.0971,281.0818,299.0926,313.1086,329.1393 |  |  | - | - | - | + | - | + | (Awang et al. 2009) |
| 38 | Flavonol  *O*-glycosides | **Quercetin-dimethyl ether-*O*-rutinoside #** | 16.92 | C_29_H_34_O_16_ | 637.1761  (2.12) | 268.0370,284.0642 |  |  | + | + | - | - | - | - | (J. Wang et al. 1997) |
| 39 | Flavone  *O*-glycosides | **Luteolin *O*-glucuronide#**  **Isomer 5** | 16.94 | C_21_H_18_O_12_ | 461.0725  (0) | 59.0134, 133.0292,286.0435 |  |  | - | - | ++ | - | + | + | (Yinron g Lu and Foo 2000) |
| 40 | Flavanone | **Hesperetin** | 17.23 | C_16_H_14_O_6_ | 301.0717  (0.22) | 65.0028, 108.0211, 136.0163, 151.0035, 164.0114 |  |  | ++ | ++ | + | - | + | - | (Aranganathan et al. 2009) |
| 41 | Flavanone | **Brosimacutin A** | 17.79 | C_20_H_22_O_6_ |  |  | 381.1302  [M + Na]^+^  (1.71) | 69.0686,  109.1016,  251.0664,  267.0652,  309.1084 | - | - | + | - | - | - | (Takashima and Ohsaki 2002) |
| 42 | Flavone | **Apigenin#** | 18.55 | C_15_H_10_O_5_ | 269.0459  (-1.23) | 65.0030, 107.0133, 117.0344, 149.0242 |  |  | + | ++ | ++ | ++ | ++ | ++ | (Adezet et al. 1986) |
| 43 | Flavone | **Diosmetin# isomer 1** | 18.86 | C_16_H_12_O_6_ | 299.0561  (0.07) | 107.0133, 151.0034, 256.0374, 284.0325 |  |  | + | ++ | + | + | + | + | (A Ulubelen, Miski, and Mabry 1981) |
| 44 | Flavanols | **Quercetin dimethyl ether** | 19.36 | C_17_H_14_O_7_ | 329.0665  (0.13) | 199.0395,  227.0339, 243.0303, 271.0246, 299.0194 |  |  | - | + | - | - | - | - | (Aranganathan et al. 2009) |
| 45 | Flavanols | **Chrysosplenol D** | 19.36 | C_18_H_16_O_8_ | 359.0770  (0.45) | 258.0171, 286.0113, 301.0346, 314.0058, 329.0299 |  |  | - | + | - | - | - | - | (Ling et al. 2010) |
| 46 | Flavone | **Cirsimaritin #** | 19.93 | C_17_H_14_O_6_ | 313.0726  (-2.25) | 117.0343, 163.0039,255.0302,283.0250 |  |  | + | ++ | +++ | ++++ | +++ | +++ | (W. Zheng and Wang 2001) |
| 47 | Flavone | **Cirsilineol #** | 19.97 | C_18_H_16_O_7_ | 343.0824  (-0.2) | 270.0168, 285.0403, 298.0116, 313.0350 |  |  | - | + | - | - | - | - | (Ulubelen  and Mabry 1981) |
| 48 | Flavan | **Pentamethoxyflavan** | 19.98 | C_20_H_24_O_6_ | 359.1497  (-0.55) | 329.1030,343.1178,359.1501 |  |  | - | - | - | - | + | - | (J.-J. Chen et al. 2007) |
| 49 | Flavone | **Dihydroxy-trimethoxyflavone** | 20.13 | C_18_H_16_O_7_ | 343.0824  (-0.04) | 242.0219,  270.0172, 298.0121, 313.0352 |  |  | - | + | - | - | - | - | (Herz and Kulanthaivel 1984) |
| 50 | Flavone | **Luteolin dimethyl ether #** | 20.23 | C_17_H_14_O_6_ | 313.0719  (-0.07) | 255.0295, 269.0454, 283.0238, 297.0395 | 315.0860  (0.99) | 271.0604,  299.0551,  315.0861 | + | ++ | - | - | - | - | (Pereda-Miranda and Delgado 1986) |
| 51 | Flavone | **Diosmetin# Isomer 2** | 20.69 | C_16_H_12_O_6_ | 299.0560  (-0.11) | 107.0145, 133.0289, 256.0385, 284.0330 |  |  | + | ++ | + | ++ | + | ++ | (GÜLAÇTI Topcu et al. 1995) |
| 52 | Flavone | **Tricin** | 20.89 | C_17_H_14_O_7_ | 329.0671  (-1.01) | 242.0220, 271.0247, 299.0194 |  |  | + | + | - | + | - | - | (Miyaichi, Segawa, and Tomimori 2006) |
| 53 | Flavone | **Acacetin # isomer 1** | 21.60 | C_16_H_12_O_5_ | 283.0615  (-0.89) | 63.0235, 107.0133, 151.0037, 240.0422, 268.0374 | 285.076  (0.27) | 55.0536,  133.0638,  242.0570,  270.0510 | - | + | - | - | - | - | (Pereda-Miranda and Delgado, 1986)  (Wollenweber et al. 1992) |
| 54 | Flavone | **Acacetin # isomer 2** | 22.06 | C_16_H_12_O_5_ | 283.0614  (-1.13) | 117.0343, 151.0036, 240.0424, 268.0379 | 285.0750  (2.5) | 124.0151,  167.0333,  242.0567,  285.0753 | - | + | ++ | +++ | ++ | ++ | (Miranda and Delgado, 1986; Topcu et al., 1996) |
| 55 | Flavone | **Velutin #** | 22.06 | C_17_H_14_O_6_ | 313.0718  (0.86) | 255.0298, 283.0247, 298.0480 |  |  | - | + | - | + | - | - | (Amaro-Luis, Herrera, and Luis 1998) |
| 56 | Flavone | **Salvigenin #** | 22.53 | C_18_H_16_O_6_ |  |  | 329.1014  (1.57) | 133.0643,  240.0770  268.0725,  296.0667 | - | + | - | ++ | - | ++ | (Yinrong Lu and Foo 2002) |
| 57 | Flavone | **Apigenin dimethyl ether#** | 24.55 | C_17_H_14_O_5_ |  |  | 299.0912  (0.67) | 256.0728, 284.0673, 299.0912, | - | - | - | + | - | + | (V U Ahmad et al. 2000) |
|  | **Lignans** | | | | | | | | | | | | | | |
| 58 | Lignan | **Hydroxylariciresinol *O*-hexoside Isomer 1** | 7.92 | C_26_H_34_O_12_ |  |  | 561.1937  [M +Na]^+^  (0.48) | 202.0588,  351.1040,  365.1191,  399.1406,  561.1935 | - | - | + | - | + | - | (Fiorentin et al. 2008) |
| 59 | Lignan | **Hydroxylariciresinol *O*-hexoside Isomer 2** | 8.13 | C_26_H_34_O_12_ |  |  | 561.1939  [M + Na]^+^  (0.79) | 351.1043,  399.1421,  561.1940 | ++ | - | - | - | - | + | (Fiorentin et al. 2008) |
| 60 | Lignan | **Pentahydroxy—methoxy oxy**  **neolignan -*O*-hexoside** | 9.06 | C_25_H_34_O_12_ |  |  | 549.1935  (3.31) | 189.0519,  352.1121,  387.1407,  549.1935 | - | + | - | - | - | - | (Kraus and Spiteller 1997) |
| 61 | Lignan | **Trihydroxy-dimethoxy-*O*-neolignan-*O* -hexoside** | 10.01 | C_26_H_36_O_12_ |  |  | 563.2090  [M +Na]^+^  (2.53) | 130.0389,  366.1252,  563.2090 | + | - | - | - | - | - | (Y. Yang, Chang, and Wu 2005) |
| 62 | Lignan | **Hydroxylariciresinol *O*-hexoside Isomer 3** | 10.02 | C_26_H_34_O_12_ |  |  | 561.1942  [M + Na]^+^  (1.21) | 131.0490,  140.0675,  163.0760,  203.0503,  366.1279,  561.1935 | + | - | - | - | - | - | (Meng, Wu, and Zhao 2010) |
| 63 | Lignan | **Armandiside** | 11.13 | C_27_H_36_O12 |  |  | 575.2105  [M + Na]^+^  (-0.83) | 259.0940,  337.0892,  381.1310,  545.1981,  575.2104 | ++ | ++ | + | - | ++ | + | (Bi et al. 2011) |
| 64 | Lignan | **Viscoloratin** | 11.13 | C_27_H_34_O_13_ |  |  | 589.1892  [M + Na]^+^  (-0.79) | 185.0566,  365.1209,  435.1223,  559.1748,  589.1899 | ++ | + | - | - | - | - | (NISIBE et al. 1990) |
| 65 | Lignan | **Fraxiresinol-*O*-**  **hexoside** | 11.13 | C_27_H_34_O_13_ | 565.1911  (3.73) | 165.0569, 312.0981, 327.1240, 339.1248 |  |  | - | - | + | - | + | - | (Piccinelli et al. 2004) |
| 66 | Lignan | **Symplocosin** | 12.15 | C_26_H_32_O_11_ | 519.1868  (2.26) | 136.0165, 151.0399, 357.1337 |  |  | + | + | + | - | + | + | (Luecha et al. 2009) |
| 67 | Lignan | **Acetoxypinoresinol *O*-hexoside** | 13.06 | C_28_H_34_O_13_ | 577.1916  (3.76) | 151.0397, 280.0733, 325.1078, 415.1387 |  |  | - | + | - | - | - | - | (Tsukamoto, Hisada, and NISHIBE 1985) |
| 68 | Lignan | **Secoisolariciresinol** | 13.82 | C_20_H_26_O_6_ | 361.1657  (0.17) | 122.0367, 147.0448,  165.0556, 179.0712 |  |  | + | + | - | - | - | - | (Lapteva, Tyukavkina, and Ryzhova 1971) |
| 69 | Lignan | **Syringaresinol** | 14.84 | C_22_H_26_O_8_ | 417.1542  (5.45) | 137.0236, 166.0270, 181.0501, 387.1071 |  |  | + | + | - | - | - | - | (Murata et al. 2010) |
| 70 | Lignan | **Saurufurin E** | 16.32 | C_20_H_22_O_6_ | 357.1346  (-0.94) | 271.0968,281.0820,299.0924,314.1152,329.1391,342.1106, |  |  | - | - | + | + | + | ++ | (W.-J. Tsai et al. 2014) |
| 71 | Lignan | **Obovaten** | 19.63 | C_20_H_20_O_5_ | 339.1232  (1.61) | 281.0825,298.1210,313.1447,326.1161 |  |  | - | - | ++ | ++ | + | + | (Lih Tsai et al., 1998) |
| 72 | Lignan | **Cinnamophilin** | 23.52 | C_20_H_24_O_5_ | 343.1554  (-0.61) | 285.1131,313.1082,327.1239,343.1559 |  |  | - | + | ++ | ++ | ++ | ++ | (T.-S. Wu et al. 1994) |
|  | **Terpenes** | | | | | | | | | | | | | | |
|  | **Apo-Carotenoid** | | | | | | | | | | | | | | |
| 73 | Megastigmanes | **Icariside B10** | 8.64 | C_19_H_32_O_8_ |  |  | 411.1987  {M + Na]^+^  (0.65) | 57.0686,  203.0507,  249.1458,  411.1987 | - | - | + | - | + | + | (Miyase and Ueno 1991) |
| 74 | Megastigmanes | **Canangaionoside**  **Isomer 1** | 11.04 | C_19_H_30_O_9_ |  |  | 425.1773  [M + Na]^+^  (2.26) | 61.0272, 203.0515,  425.1773 | - | - | + | - | + | ++ | (Matsunami et al. 2010) |
| 75 | Megastigmanes | **Canangaionoside**  **Isomer 2** | 11.53 | C_19_H_30_O_9_ |  |  | 425.1778  [M + Na]^+^  (1.08) | 425.1781 | - | - | + | + | ++ | + | (Matsunami et al. 2010) |
| 76 | Megastigmanes | **Icariside B6** | 13.06 | C_19_H_32_O_7_ |  |  | 395.2035  [M +Na]^+^  (1.45) | 81.0698,  203.0507 | - | - | + | - | + | - | (X. Wang et al. 2008) |
| 77 | Megastigmanes | **Alangionosides I** | 13.16 | C_24_H_42_O_11_ |  |  | 529.2615  [M +Na]^+^  (0.7) | 140.0678,  335.0936,  529.2608 | - | - | + | - | + | - | (OTSUKA et al. 1995) |
|  | **Monoterpenes** | | | | | | | | | | | | | | |
| 78 | [Iridoids derivative](https://coconut.naturalproducts.net/search?q=parent%3Airidoids-and-derivatives&page=1&type=filters) | **Swertiaside A** | 8.43 | C_23_H_28_O_12_ |  |  | 497.1625  (0.6) | 128.9523  361.0908,  497.1625 | - | - | + | - | + | - | (Kitajima et al. 2005) |
| 79 | [Iridoids derivative](https://coconut.naturalproducts.net/search?q=parent%3Airidoids-and-derivatives&page=1&type=filters) | **Lucidumoside D** | 11.13 | C_27_H_36_O_13_ |  |  | 591.2037  [M + Na]^+^  (2.00) | 232.0696,  351.1030,  365.1195,  591.2037 | - | - | + | - | + | - | (Z.-D. He et al. 2001) |
| 80 | Monoterpene | **Rosiridin** | 11.75 | C_16_H_28_O_7_ | 331.1764  (0.01) | 55.0185, 59.0129, 71.0139, 85.0288, 101.0234 |  |  | - | - | + | - | + | - | (Yoshikawa et al. 2008) |
| 81 | Monoterpene | **Betulabuside A** | 12.35 | C_16_H_28_O_7_ |  |  | 355.1721  [M + Na]^+^  (1.76) | 93.0696,  107.0840,  203.0506,  355.1721 | + | - | ++ | - | ++ | ++ | (Rumalla et al. 2010) |
| 82 | Monoterpene | **Isopiperitenone** | 12.51 | C_10_H_14_O |  |  | 151.1114  (2.36) | 58.0646,  77.0380,  91.0535 | - | - | - | - | - | + | (Kirimer et al. 1993) |
| 83 | Monoterpene | **Icariside C1**  **Isomer 1** | 16.98 | C_21_H_38_O_8_ |  |  | 441.2457  [M +Na]^+^  (0.46) | 203.0523,  261.1820,  441.2459 | - | - | + | - | + | - | (Koyama et al. 2010) |
| 84 | Monoterpene | ***P*-Menth-3-en-2-one** | 18.26 | C_10_H_16_O |  |  | 153.1272  (1.14) | 51.0224,  55.0537,  77.0381,  91.0534 | - | - | - | - | - | + | (Kurobayashi et al. 1991) |
| 85 | Monoterpene | **Icariside C1 Isomer2** | 18.36 | C_21_H_38_O_8_ |  |  | 441.2452  [M + Na]^+^  (1.89) | 100.1125,  203.0530,  441.2458 | - | - | + | - | - | + | (Fiorentino et al. 2006) |
|  | **Sesquiterpenoid** | | | | | | | | | | | | | | |
| 86 | Sesqui  terpenoid | **Hydroxy-dimefilm thylnona-2,8-dien-l,4-olide** | 9.20 | C_11_H_16_O_3_ |  |  | 197.1168  (1.99) | 55.0539,  67.0539,  77.0380,  91.0537,  105.0695 | - | ++ | - | + | - | - | (Marco et al. 1997) |
| 87 | Germacrane | **Neurolenin G** | 11.80 | C_21_H_28_O_8_ |  |  | 431.1681  [M + Na]^+^  (-1.42) | 157.0634,  167.0681,  175.0351,  204.0748,  257.0763, | - | + | - | - | - | - | (Passreiter Sandoval-Ramirez, and Wright 1999) |
| 88 | Sesqui  terpenoid | **Eurycolactone A** | 11.85 | C_20_H_24_O_7_ |  |  | 399.1418  [M +Na]^+^  (-1) | 81.0687,  95.0478,  125.0578,  136.0492,  204.0762,  267.1222 | - | + | - | - | - | - | (Ang, Hitotsuyanagi, and Takeya 2000) |
| 89 | Germacrane | **11b,13-dihydro-6a-hydroxytomentosin** | 15.04 | C_15_H_22_O_4_ |  |  | 289.1407  [M + Na]^+^  (0.95) | 67.0544,  91.0532,  123.0796,  131.0842,  155.0832,  249.0870 | - | + | - | - | - | - | (Cheng et al. 2011) |
| 90 | Sesqui  terpenoid | **Karinolide 1** | 15.15 | C_20_H_24_O_8_ | 391.1397  (0.31) | 59.0136, 83.0499,  137.0612,314.1162,318.1452,345.1319 |  |  | - | - | - | + | - | - | (Polonsky et al. 1982) |
| 91 | Eudesmane | **hydroxy1,4a-dimethyl-7-(prop-1-en-2-yl)-4,4a,5,6,7,8-hexahydronaphthalen-2(3H)-one** | 16.67 | C_15_H_22_O_2_ |  |  | 235.1687  (2.3) | 55.0537,  67.0537,  91.0533,  105.0701,  131.0854,  149.0968 | - | + | - | - | - | - | (J. Xu et al. 2012) |
| 92 | Germacrane | **Angeloyloxy-1-hydroxy-2-oxoxantha-3,11-dien-6,12-olide** | 16.88 | C_20_H_26_O_6_ |  |  | 385.1617  [M +Na]^+^  (1.86) | 241.0857,  342.1430,  357.1664.  385.1618 | - | - | - | - | + | ++ | (Cui et al. 1999) |
| 93 | Caryophyllane | **Buddledin C** | 17.33 | C_15_H_22_O |  |  | 219.1744  (-0.36) | 55.0537,  67.0536,  81.0695,  91.0542,  105.0695,  119.0848 | - | - | - | + | - | - | (Sadyrbekov et al. 2006) |
| 94 | Eudesmane | **Alloalantolactone** | 17.60 | C_15_H_20_O_2_ |  |  | 233.1541  (-1.35) | 55.0530,  67.0536,  79.0540,  105.0691,  143.0839 | - | + | - | - | - | - | (Bohlmann, Zdero, and Ahmed 1982) |
| 95 | Caryophyllane | **Kobusone** | 18.35 | C_14_H_22_O_2_ |  |  | 223.1691  (1.18) | 55.0539,  69.0696,  81.0695,  95.0847 | - | + | - | - | - | - | (HEYMANN et al. 1994) |
| 96 | Germacrane | **Lychnopholide**  **isomer 1** | 19.07 | C_20_H_22_O_6_ |  |  | 359.1485  (1.26) | 68.9966,  159.0806,  187.0754,  201.0903,  256.0723,  271.0953, | - | - | - | - | + | ++ | (Bohlmann, Singh, et al. 1982) |
| 97 | Eremophilane | **Virgaurenolide A** | 19.37 | C_20_H_22_O_5_ |  |  | 343.1537  (1.32) | 83.0486,  135.0798,  239.1058,  255.1364 | - | - | - | - | + | ++ | (Tori et al. 2006) |
| 98 | Eudesmane | **Acetoxy-ll-hydroxy-3b-(2-methylbutyryloxy)-eudesm6-en-8-one**  **Isomer 1** | 20.53 | C_20_H_32_O_5_ |  |  | 375.2136  [M + Na]^+^  (1.61) | 135.0775,  163.0757,  199.1090,  245.1162, | - | + | - | - | - | - | (Bohlman, Borthakur, et al. 1982) |
| 99 | Eudesmane | **Hydroxy-a-eudesmol-11-O-b-D-glucopyranoside** | 20.78 | C_21_H_36_O_7_ |  |  | 423.2351  [M + Na]^+^  (0.48) | 69.0706,  137.1327,  203.0526,  365.1939 | - | - | + | - | + | - | (H. Yang et al. 2012) |
| 100 | Eudesmane | **Acetoxy-ll-hydroxy-3b-(2-methylbutyryloxy)-eudesm6-en-8-one**  **Isomer 2** | 20.79 | C_20_H_32_O_5_ |  |  | 375.2139  [M +Na]^+^  (0.55) | 69.0708,  93.0704,  119.0845,  301.1754 | - | + | - | - | - | - | (Bohlman,Borthakur, et al. 1982) |
| 101 | Germacrane | **Desacetyl-**  **neurolenin G** | 21.00 | C_19_H_26_O_7_ |  |  | 389.1591  [M +Na]^+^  (-6.67) | 68.9967,  83.0487,  175.0749,  203.0698 | - | - | - | - | - | + | (Passreiter Sandoval-Ramirez, and Wright 1999) |
| 102 | Germacrane | **2,11-dimethyl-7-methylidene-6,12-dioxo-5,14-dioxatricyclo[9.2.1.0⁴,⁸]tetradeca-1(13),2-dien-9-yl 2-methylbutanoate** | 21.09 | C_20_H_24_O_6_ |  |  | 383.1465  [M +Na]^+^  (-0.55) | 267.0986,  282.1219,  295.0933, | - | - | ++ | + | - | - | (SchmedaHirschmann et al. 1986) |
| 103 | Guaiane | **Methacryloyloxy dehydrocostus lactone** | 22.06 | C_19_H_22_O_4_ |  |  | 315.1593  (-0.17) | 79.0534,  155.0847,  169.1004,  272.0670,  300.0627 | - | + | - | - | - | - | (Bohlman and Gupta 1981) |
| 104 | Humulane | **Tetramethyl8-bicyclo[8.1.0]undecene-2,9-diol** | 22.51 | C1_5_H_26_O_2_ |  |  | 261.1827  [M + Na]^+^  (-0.7) | 91.0517,  105.0697,  121.0634,  156.0941,  167.0865 | - | - | + | - | + | - | (F. Xu et al. 2004)v |
| 105 | Eudesmane | **11-Hydroxyjasionone** | 22.87 | C_15_H_24_O_2_ |  |  | 259.1667  [M + Na]^+^  (0.64) | 67.0532,  79.0541,  91.0541,  135.0782,  175.1114 | - | ++ | - | + | - | + | (Ahmed et al. 1988) |
| 106 | Germacrane | **Lychnopholide**  **isomer 2** | 22.98 | C_20_H_22_O_6_ |  |  | 359.1499  (-2.48) | 123.0435,  187.0745,  215.0699,  229.0851,  257.0802,  299.1271 | - | - | + | ++ | + | + | (Bohlmann et al. 1980) |
| 107 | Guaiane | **Dehydrooopodin** | 23.48 | C_20_H_24_O_4_ |  |  | 351.1570  [M + Na]^+^  (-0.48) | 55.0532,  69.0688,  105.0696,  121.1000,  145.1019 | - | ++ | + | - | - | ++ | (Suzuki et al. 2007) |
| 108 | Germacrane | **Euserotin** | 23.79 | C_21_H_26_O_6_ |  |  | 397.1620  [M +Na]^+^  (-1.96) | 85.0629,  143.0840,  215.0697,  283.1675 | - | + | - | - | - | - | (Bohlmann et al. 1985) |
| 109 | Sesqui  terpenoid | **Eurycolactone D** | 23.85 | C_18_H_22_O_5_ |  |  | 341.1375  [M +Na]^+^  (-4.81) | 173.0954,  201.0902,  229.0851,  328.1293 | - | - | + | + | + | ++ | (Ang et al. 2002) |
| 110 | Germacrane | **Ineupatorolide A** | 24.71 | C_20_H_30_O_6_ |  |  | 389.1957  [M + Na]^+^  (-5.07) | 213.0902,  227.1051,  241.0852,  255.1007,  287.1269,  297.1477 | - | - | - | ++ | - | + | (Maruyama et al. 1995) |
| 111 | Eremophilane | **Methylbutyryloxy)eremophil-3,7(11),8-trien-8,12-olid-15-oic acid methyl ester.** | 25.16 | C_21_H_26_O_6_ |  |  | 267.0983,  283.1290,  295.0933,  369.1662 | 397.1620  [M + Na]^+^  (0.36) | - | - | + | ++ | ++ | +++ | (Fei et al. 2007) |
| 112 | Guaiane | **Blumeaene K** | 25.17 | C2_0_H_32_O_7_ |  |  | 407.2060  [M +Na]^+^  (-5.22) | 59.0487,  229.0848,  255.1011,  301.1789 | - | - | + | - | - | + | (Shirota et al. 2011) |
| 113 | Eudesmane | **Dihydroxy-cadin-10(14)-ene** | 25.21 | C_15_H_26_O_2_ |  |  | 261.1824  [M +Na]^+^  (-0.18) | 67.0539,  91.0537,  133.1005,  175.0736 | - | - | ++ | - | + | ++ | (El-Askary, Meselhy, and Galal 2003) |
|  | **Diterpenoid** | | | | | | | | | | | | | | |
| 114 | Abeoabietane | **Teuvincenone A** | 18.45 | C_20_H_22_O_6_ | 357.1341  (0.27) | 73.0291,  213.1283,241.0872,257.1181,272.1051,286.1204,300.1361,329.1388 |  |  | - | - | + | ++ | - | - | (M. Xu et al. 2011) |
| 115 | Labdane | **Isocupressic acid** | 19.16 | C_20_H_32_O_3_ |  |  | 343.2242  [M +Na]^+^  (0.41) | 79.0542,  95.0839,  119.0844,  119.0844,  329.0653 | - | + | - | - | - | - | (Jian Wu and Zhao 2010) |
| 116 | Diterpenoid | **Sibiricinone C** | 19.42 | C_21_H_32_O_6_ |  |  | 381.2244  (7.38) | 95.0490,  107.0856,  123.0796,  145.1004,  381.2244 | - | ++ | - | - | - | + | (Boalino et al. 2004) |
| 117 | Abietane | **Rosmaquinone #** | 19.42 | C_20_H_24_O_5_ |  |  | 345.1693  (-0.15) | 83.0486, 215.1057, 243.1005, 257.1165 | - | - | - | - | + | + | (González et al. 1989) |
| 118 | Nagilactone | **Nagilactone I** | 19.47 | C_20_H_24_O_7_ | 375.1451  (-0.6) | 135.0812,137.0607,149.0603,257.1564,259.1702,283.0975,298.1208, 300.1363,315.1597, |  |  | - | - | - | - | + | ++ | (Ying et al. 1990) |
| 119 | Labdane | **3,18,19-trihydroxy-entlabda-8(17),13-dien-16,15-olide** | 20.30 | C_20_H_30_O_5_ |  |  | 351.2145  (-0.11) | 55.0542,  81.0694,  95.0857,  107.0838,  123.0807 | + | + | - | - | - | - | (L. Chen et al. 2006) |
| 120 | Colensane and Clerodane | **5,9-dihydroxy-7,8-dimethyl-7-[2-(5-oxo-2H-furan-4-yl)ethyl]- hexahydro-1Hbenzo[d]isobenzofuran-3-one #** | 20.69 | C_20_H_26_O_6_ | 361.1650  (1.8) | 95.0496,  161.0969,189.0919,231.1388,285.1486, 315.1595 |  |  | - | - | + | ++ | + | + | (Esquivel et al. 1989) |
| 121 | Pimarane and Isopimarane | **Negundoin G** | 21.04 | C_20_H_30_O_3_ |  |  | 341.2084  [M + Na]^+^  (2.5) | 169.1019,  243.1008,  268.1448,  283.1687 | - | + | - | - | - | - | (C.-J. Zheng et al. 2010) |
| 122 | Labdane | **Phlogantholide-A** | 21.25 | C_20_H_30_O_4_ |  |  | 357.2026  [M + Na]^+^  (3.16) | 55.0536,  162.0673,  197.0975,  298.0826,  326.0800 | - | + | - | - | - | - | (Barua et al. 1985) |
| 123 | - | **Gaultheric acid** | 21.75 | C1_9_H_24_O_4_ |  |  | 339.1590  [M + Na]^+^  (1.64) | 55.0173,  67.0532,  95.0845,  155.0842,  179.0866,  241.0853 | - | + | - | - | - | - | (Zhang et al. 1999) |
| 124 | Labdane | **Methoxyvelutine C isomer 1** | 22.37 | C_21_H_28_O_7_ |  |  | 393.1912  (-0.98) | 59.0489,  187.0749,  215.0704,  273.1117 | - | - | + | ++ | - | - | (Argyropoulou, Karioti, and Skaltsa 2009) |
| 125 | Labdane | **Limbatenolide E** | 22.97 | C_20_H_22_O_4_ |  |  | 327.1587  (1.31) | 55.0542,  69.0333,  169.1003,  229.1211 | - | + | - | - | - | - | (Farooq et al. 2007) |
| 126 | Labdane | **Methoxyvelutine C isomer 2** | 22.98 | C_21_H_28_O_7_ |  |  | 393.1901  (0.77) | 59.0487,  187.0744,  273.1107, | - | - | - | - | + | ++ | (Argyropoulou, Karioti, and Skaltsa 2009)v |
| 127 | Colensane and Clerodane | **Conyzolide** | 23.24 | C_20_H_24_O_6_ | 359.1519  )-5.33( | 135.0812,151.0765,165.0556,217.0872,243.1028,272.1056,283.0975,298.1207,313.1445,326.1159,341.1395 |  |  | - | - | + | + | + | ++ | (Shakirullah et al. 2011) |
| 128 | Colensane and Clerodane | **Caseamembrins K** | 23.34 | C_24_H_32_O_7_ |  |  | 455.2049  [M + Na]^+^  (-2.05) | 129.0519,  283.1691,  305.1503,  455.2049 | - | + | - | - | - | - | (Shen et al. 2005) |
| 129 | Abeoabietane | **Triptobenzene H** | 23.38 | C_21_H_28_O_4_ |  |  | 345.2059  (0.41) | 55.0538,  147.0802,  187.0751,  229.1218 | - | - | - | + | - | ++ | (K. Li et al. 1997) |
| 130 | Abeoabietane | **Candesalvone B methyl ester #** | 24.15 | C_20_H_26_O_6_ | 361.1654  (0.49) | 247.0978,273.1130,289.1440,301.1443,317.1757,343.1549 |  |  | - | - | ++ | - | + | - | (Nabih et al. 2024) |
| 131 | Labdane | **Limbatenolide E**  **isomer 1** | 24.30 | C_20_H_22_O_4_ |  |  | 327.1590  (-0.01) | 185.0954,  201.1270,  213.0907,  253.1219,  268.1452,  283.1688 | - | + | ++ | ++ | - | - | (Farooq et al. 2007) |
| 132 | Colensane and Clerodane | **Floribundic ester** | 24.29 | C_21_H_26_O_5_ |  |  | 381.1674  [M +Na]^+^  (0.21) | 69.0693,  93.0697,  305.1505,  381.1685 | - | + | - | - | - | - | (Su et al. 2008) |
| 133 | Abietane | **Rosmaquinone#**  **Isomer 1** | 24.41 | C_20_H_24_O_5_ |  |  | 345.1699  (-0.93) | 157.1010, 201.1272, 213.0906, 283.1689 | - | + | - | - | - | - | (González et al. 1989) |
| 134 | Labdane | **Limbatenolide E isomer 2** | 24.61 | C_20_H_22_O_4_ |  |  | 327.1587  (1.36) | 105.0695,  119.0849,  199.0746,  223.1107 | - | + | - | - | + | ++ | (Farooq et al. 2007) |
| 135 | Abeoabietane | **Taiwaniaquinone D** | 24.70 | C_20_H_24_O_4_ |  |  | 329.1746  (0.43) | 55.0538,  69.0695,  83.0487,  119.0850,  201.0906 | - | - | + | - | - | - | (Lin, Fang, and Cheng 1996) |
| 136 | Abeoabietane | **Candesalvone A #** | 24.86 | C_20_H_26_O_4_ |  |  | 331.1904  (-2.02) | 55.0540,  69.0694,  81.0696,  219.0640,  257.1168 | - | - | ++ | + | - | - | (Mendes et al. 1989) |
| 137 | Abietane | **Rosmaquinone#**  **Isomer 2** | 24.96 | C_20_H_24_O_5_ |  |  | 345.1690  (0.79) | 201.1269,  213.0906,  268.1454,  283.1686 | - | + | - | - | - | - | (González et al. 1989) |
| 138 | Abeoabietane | **Deoxycoleon U #** | 25.02 | C_20_H_26_O_4_ |  |  | 331.1897  (1.89) | 55.0536,  69.0693,  81.0696,  245.1163,  257.1165 | - | - | - | - | + | ++ | (Fraga et al. 2005) |
| 139 | Abietane | **Hydroxy-7-*0*- methyl rosmanol** | 25.17 | C_21_H_28_O_6_ | 375.1818  (-1.02) | 233.0821,248.1021,273.1133,289.1446,301.1447,343.1551 |  |  | - | - | + | +++ | ++ | - | (Takenaka et al. 1997) |
| 140 | Abeoabietane | ***O*-Methyl**  **candesalvone B** | 25.83 | C_21_H_28_O_6_ | 375.1810  (0.65) | 233.0815,247.0973,273.1131,289.1441,301.1440,343.1544 |  |  | - | - | + | - | + | - | (Janicsák et al. 2003) |
| 141 | Abietane | **Hydroxy-*O*-Methylrosmanol** | 26.13 | C_22_H_30_O_6_ | 389.1973  (-0.63) | 233.0819,247.0976,273.1133,289.1443,301.1445,329.1752,343.1549,389.1972 |  |  | - | - | - | - | + | ++ | (Takenaka et al. 1997) |
| 142 | Colensane and Clerodane | **Hydroxyclerodan-15-oic acid** | 26.34 | C_20_H_36_O_3_ |  |  | 347.2551  [M + Na]^+^  (1.82) | 81.0703,  95.0847,  107.0863,  347.2546 | - | - | - | + | - | ++ | (H. He et al. 2003) |
| 143 | Abietane | **Methyl**  **cryptotanshinone** | 26.39 | C_20_H_22_O_3_ |  |  | 311.1646  (-1.01) | 55.0539, 201.1271,  241.1584,  255.1374,  283.1688 | + | + | - | - | - | - | (Al Yousuf et al. 2002) |
| 144 | Norlabdane | **Methyl 3-oxo-18-hydroxylabda-8(17),13E-dien-15-oate** | 26.64 | C_21_H_32_O_4_ |  |  | 349.2352  (5.04) | 57.0685,  79.0535,  93.0684,  109.1018,  119.0841,  145.1008 | - | + | - | - | - | + | (Garbarino, Chamy, and Gambaro 1986) |
| 145 | Cassane | **Pulcherralpin** | 26.95 | C_30_H_40_O_7_ | 511.2699  (-2.03) | 259.1335,327.1232,343.1543,511.2696 |  |  | - | - | + | - | + | ++ | (Ragasa, Hofileña, and Rideout 2002) |
| 146 | Colensane and Clerodane | **Acetoxy-ent - cleroda-3, 13-diene- 17-oic acid-16,15-olide** | 27.05 | C_22_H_30_O_6_ |  |  | 413.1929  [M +Na]^+^  (1.09) | 83.0482,  95.0831,  107.0851,  121.1009,  177.1639 | - | - | - | + | - | ++ | (Zdero, Bohlmann, and King 1992) |
| 147 | Verticillane | **Ent-verticillanediol** | 27.20 | C_20_H_34_O_2_ |  |  | 329.1739  [M + Na]^+^  (-5.89) | 57.0691,,  69.0697,  187.1107,  219,1006 | - | - | + | ++ | + | ++ | (Nagashima et al. 2005) |
| 148 | Abietane | **Miltirone #** | 27.20 | C_19_H_22_O_2_ |  |  | 283.1697  (-1.59) | 157.1003,  185.0954,  201.1270,  253.1221 | - | + | - | - | - | - | (Fronza et al. 2011) |
| 149 | Abietane | **Cyrtophyllone B** | 27.30 | C_20_H_28_O_4_ | 331.1919  (-1.23) | 259.0982, 316.1679, 331.1916 |  |  | - | - | + | + | + | ++ | (Miron-Lopez et al. 2014) |
| 150 | Podocarpane | **Tetraene-24-nor-friedelane-29-oic acid methylester** | 28.82 | C_30_H_40_O_6_ | 495.2749  (-1.59) | 259.1340,343.1547,467.2794,495.2746 |  |  | - | - | + | ++ | + | + | (Ankli et al. 2000) |
| 151 | Labdane | **Leopersin G** | 29.12 | C_20_H_30_O_4_ |  |  | 357.2034  [M + Na]^+^  (0.67) | 57.0698,  75.0249,  95.0852,  281.1525 | - | + | - | - | - | - | (Wongsa, Kanokmedhakul, and Kanokmedhakul 2011) |
|  | **Triterpene** | | | | | | | | | | | | | | |
| 152 | Quassinoids | **Vilmorinine C** | 15.51 | C_20_H_24_O_8_ | 391.1407  (-2.71) | 137.0603,273.1498,299.0924,314.1157,329.1385,345.1345 |  |  | - | - | - | - | - | + | (Takeya et al. 1998) |
| 153 | Ursane and Taraxastane | **Myrianthic acid**  **isomer 1** | 20.99 | C_30_H_48_O_6_ | 503.3375  (0.87) | 409.3103, 453.3004, 485.3265, 503.3374 |  |  | + | + | - | - | - | - | (W. Li et al. 2009) |
| 154 | Ursane and Taraxastane | **Brahmic acid** | 21.65 | C_30_H_48_O_6_ | 503.3373  (1.85) | 161.0243, 193.0501, 485.3252, 503.3366 |  |  | + | ++ | - | - | - | - | (Randriamampionona et al. 2007) |
| 155 | Ursane and Taraxastane | **Myrianthic acid**  **Isomer 2** | 22.77 | C_30_H_48_O_6_ | 503.3369  (1.46) |  |  |  | + | + | - | - | - | - | (Sandjo et al. 2010) |
| 156 | Ursane and Taraxastane | **Asiatic acid** | 22.82 | C_30_H_48_O_5_ | 487.3425  (0.56) |  |  |  | + | + | - | - | - | - | (Viqar Uddin Ahmad, Bano, and Bano 1986) |
| 157 | Triterpene | **Epipomolic acid** | 23.74 | C_30_H_48_O_4_ | 471.3473  (1.5) | 57.0333, 137.0237, 441.3351, 455.3170 |  |  | - | + | - | - | - | - | (Kuroyanagi et al. 2001) |
| 158 | Ursane and Taraxastane | **Tormentic acid** | 24.85 | C_30_H_48_O_5_ | 487.3420  (1.58) | 58.0056,  423.3260,  469.3314, |  |  | ++ | ++ | + | ++ | + | ++ | (Banno et al. 2004) |
| 159 | Ursane and Taraxastane | **Dihydroxy-3- oxo-12-ursen-28-oic acid isomer 1** | 25.06 | C_30_H_46_O_5_ | 485.3259  (2.83) | 58.0052, 369.2777, 425.3412, 457.3310,  469.3311,470.3330 |  |  | - | + | - | - | - | - | (T.-H. Lee et al. 2001) |
| 160 | Oleanane | **Bodinin** | 25.32 | C_29_H_44_O_4_ | 455.3162  (1.27) | 83.0495, 351.2675, 381.2799, 393.3162,413.3314,455.3161 |  |  | - | + | - | - | - | - | (Zhu et al ., 1999) |
| 161 | Ursane and Taraxastane | **Dihydroxy-3- oxo-12-ursen-28-oic acid isomer 1** | 25.46 | C_30_H_46_O_5_ | 485.3259  (1.97) | 59.0123,  191.1446,  441.3350, 467.3148, |  |  | - | ++ | - | + | - | - | (T.-H. Lee et al. 2001) |
| 162 | Oleanane | **Echinocystic acid**  **Isomer 1** | 25.67 | C_30_H_48_O_4_ | 471.3471  (2.09) | 71.0495, 471.3474 |  |  | + | ++ | + | ++ | + | + | (Krasteva et al. 2007) |
| 163 | Oleanane | **Enoxolone** | 25.72 | C_30_H_46_O_4_ | 469.3312  (2.53) | 57.0340,  397.3094,  409.3109,  425.3414,  469.3309 |  |  | + | ++ | - | ++ | - | - | (Sanduja et al. 1984) |
| 164 | Oleanane | **Echinocystic acid**  **Isomer2** | 25.73 | C_30_H_48_O_4_ | 471.3468  (2.61) | 409.3444,  425.3425,  471.2273 | 473.3626  (-1.3) | 81.0697,  95.0850,  119.0850,  121.1010,  191.1786 | + | ++ | + | ++ | + | + | Krasteva  et al., 2007) |
| 165 | Triterpene | **Macrocarpin B** | 26.08 | C_28_H_34_O_7_ | 481.2230  (-0.43) | 289.1439,301.1440,343.1548,361.1649,481.2232 |  |  | - | - | - | + | - | + | (Chávez et al. 2000) |
| 166 | Quassinoids | **Quassin** | 26.19 | C_22_H_28_O_6_ |  |  | 411.1772  [M + Na]^+^  (0.73) | 255.0613,  283.1283,  310.1166,  383.1822 | - | - | - | - | - | + | (Grandolini et al. 1987) |
| 167 | Triterpene | **Echinocystic acid Isomer3** | 26.28 | C_30_H_48_O_4_ |  |  | 473.3626  (-1.3) | 81.0697,  95.0850,  119.0850,  121.1010,  191.1786 | - | + | - | + | - | - | (Tong et al. 2004) |
| 168 | Ursane and Taraxastane | **Oxo-ursolic acid**  **Isomer 1** | 26.33 | C_30_H_46_O_4_ | 469.3320  (0.72) | 59.0138, 71.0485, 365.2837, 393.3143, 409.3467, 425.3395,454.3390 |  |  | - | + | - | - | - | - | (Syamasundar, Mallavarapu, and Krishna 1991) |
| 169 | Triterpene | **Moronicacid** | 26.43 | C_30_H_46_O_3_ |  |  | 455.3511  (0.82) | 95.0854,  109.1005,  147.1154,  191.1791,  201.1627 | - | ++ | - | - | - | + | (Ahsan et al. 1995) |
| 170 | Ursane and Taraxastane | **Pomolic acid** | 26.48 | C_30_H_48_O_4_ | 471.3474  (1.33) | 407.3313, 453.3366, 471.3474 |  |  | + | +++ | ++ | ++ | ++ | ++ | (Kuang et al. 1989) |
| 171 | Ursane and Taraxastane | **Oxo-ursolic acid**  **Isomer 2** | 26.53 | C_30_H_46_O_4_ | 469.3318  (1.11) | 71.0503  407.3309, 425.3413, 453.3358, 469.3320 |  |  | + | ++ | + | ++ | + | + | (Syamasundar, Mallavarapu, and Krishna 1991) |
| 172 | Ursane and Taraxastane | **Punicanolic acid** | 26.89 | C_30_H_50_O_4_ | 473.3635  (0.42) | 369.3157,  413.3415,  455.3517 |  |  | + | ++ | - | + | - | + | (Xie et al. 2008) |
| 173 | Oleanane | **Oleanolic acid**  **Isomer 1** | 26.89 | C_30_H_48_O_3_ |  |  | 457.3676  (0.44) | 95.0852,  135.1166,  189.1635,  203.1787 | - | + | - | - | - | - | (Hernández et al. 1987) |
| 174 | Ursane and Taraxastane | **Ursanoic acid** | 26.99 | C_30_H_44_O_5_ | 483.3113  (0.57) | 151.0766,  421.3100,  465.3006,  483.3111 |  |  | - | + | - | - | - | + | (Traoré et al. 2008) |
| 175 | Friedelane | **6-oxo-dihydropristimerol-23-oic acid.** | 27.19 | C_30_H_40_O_7_ | 511.2700  (-1.6) | 259.1337,343.1549 |  |  | - | - | - | ++ | - | + | (Oramas-Royo et al. 2010) |
| 176 | Ursane and Taraxastane | **oxo-ursolic acid**  **Isomer 3** | 27.24 | C_30_H_46_O_4_ | 469.3316  (1.79) | 57.0341, 407.3315, 451.3206, 469.3319 |  |  | + | ++ | + | + | + | + | ((Syamasundar, Mallavarapu, and Krishna 1991) |
| 177 | Oleanane | **Echinocystic acid** | 27.24 | C_30_H_48_O_4_ | 471.3469  (0.61) | 407.3312,  471.3473 |  |  | + | ++ | ++ | +++ | ++ | ++ | (Jianming Wu et al. 2010) |
| 178 | Ursane and Taraxastane | **Asiatic acid** | 27.26 | C_30_H_48_O_5_ | 487.3423  (1.82) | 427.3581,  469.3295,  487.3425 |  |  | + | ++ | + | + | + | + | (D.-Y. Lee et al. 2010) |
| 179 | Triterpene | **Furanoganoderic acid** | 27.50 | C_30_H_38_O_7_ | 509.2538  (1.18) | 259.1334,275.1279, 343.1548 |  |  | - | - | - | + | - | - | (Nishitoba et al. 1989) |
| 180 | Oleanane | **Hederagenin#**  **Isomer 1** | 27.55 | C_30_H_48_O_4_ | 471.3467  (2.54) | 425,3329,471.3471 |  |  | + | ++ | + | +++ | + | ++ | (K.-T. Lee et al. 2000) |
| 181 | Oleanane | **Enoxolone** | 27.60 | C_30_H_46_O_4_ |  |  | 471.3466  (0,05) | 175.1478,  189.1634,  217.1581,  425.3411,  471.3471 | - | +++ | + | ++ | - | + | (Kir’yalov et al. 1970) |
| 182 | Oleanane | **Hederagenin #**  **Isomer 2** | 27.80 | C_30_H_48_O_4_ | 471.3469  (2.06) | 288.1361,  407.3311, 425.3423,  477.0055 |  |  | + | ++ | ++ | +++ | ++ | ++ | (K.-T. Lee et al. 2000) |
| 183 | Oleanane | **Hederagenin #**  **Isomer 3** | 27.86 | C_30_H_48_O_4_ | 471.3482  (-0.21) | 471.3480 |  |  | + | ++ | ++ | +++ | ++ | ++ | (K.-T. Lee et al. 2000) |
| 184 | Tirucallane and Euphane | **Trisnor-3-oxo-lanost-9(11)-en-24-oic acid**  **Isomer 1** | 27.86 | C_27_H_42_O_3_ |  |  | 415.3205  (0.14) | 95.0851,  107.0854,  121.1008,  135.1165,  147.1164 | - | - | - | ++ | + | + | (Wada and Tanaka 2000) |
| 185 | Tirucallane and Euphane | **Trisnor-3-oxo-lanost-9(11)-en-24-oic acid**  **Isomer 2** | 27.86 | C_27_H_42_O_3_ |  |  | 437.3032  [M + Na]^+^  (0.14) | 81.0694,  95.0855,  119.0848,  135.1163,  189.1639 | - | - | - | ++ | - | + | (Wada and Tanaka 2000) |
| 186 | Oleanane | **Hederagenin #**  **Isomer 4** | 28.01 | C_30_H_48_O_4_ | 471.3472  (2.03) | 221.0820,  471.3478, |  |  | + | ++ | ++ | +++ | + | ++ | (Krasteva et al. 2006) |
| 187 | Malabaricane | **Acetyljaspiferal E** | 28.01 | C_24_H_32_O_6_ |  |  | 439.2092  [M + Na]^+^  (0.19) | 107.0845,  255.0623,  283.1297,  295.0932,  310.1176,  411.2159 | - | - | - | - | + | ++ | (Aoki et al. 2007) |
| 188 | Ursane and Taraxastane | **Methyl euscaphate** | 28.06 | C_31_H_50_O_5_ | 501.3575  (1.60) | 381.3152,  423.3262, 469.3312 |  |  | - | + | - | - | - | - | (Delgado, Hernández, and Pereda-Miranda 1989) |
| 189 | Oleanane | **Maslinic acid #**  **isomer 1** | 28.26 | C_30_H_48_O_4_ |  |  | 473.3626  (-0.55) | 135.1165,  189.1634,  201.1630,  427.3568 | - | ++ | + | ++ | + | ++ | (Moghaddam et al. 2010) |
| 190 | Ursane and Taraxastane | **Micromeric acid** | 28.32 | C_30_H_46_O_3_ |  |  | 455.3509  (2.34) | 95.0850,  119.0850,  187.1474,  201.1630 | - | + | + | + | + | + | (Altinier et al. 2007) |
| 191 | Oleanane | **Maslinic acid #**  **isomer 2** | 28.41 | C_30_H_48_O_4_ | 471.3469  (2.07) | 471.3480 |  |  | + | ++ | ++ | +++ | ++ | ++ | ( Mooi et al. 2010) |
| 192 | Oleanane | **Githagenin** | 28.87 | C_30_H_46_O_4_ | 469.3316  (-0.78) | 393.3151, 437.3044, 469.3317 |  |  | + | ++ | + | + | - | - | (Wedekind and Krecke  1926) |
| 193 | Oleanane | **Hederagenin#**  **isomer 5** | 29.23 | C_30_H_48_O_4_ | 471.3470  (2.25) | 471.3462 |  |  | + | ++ | ++ | ++ | ++ | ++ | (K.-T. Lee etal. 2000) |
| 194 | Triterpene | **Globostellatic acid methyl ester** | 29.68 | C_33_H_46_O_5_ | 521.3244  (5.14) | 409.3448,  454.3398,  521.3244 |  |  | - | + | - | + | - | - | (Aoki et al. 2007) |
| 195 | Oleanane | **Oleanolic acid #**  **Isomer 2** | 29.84 | C_30_H_48_O_3_ | 455.3527  (0.88) | 455.3525, |  |  | + | ++ | +++ | ++++ | +++ | +++ | (Acebey-Castellon et al. 2011) |
| 196 | Ursane and Taraxastane | **ursa-9(11), 12-dien-3-one (marsformosanone)** | 30.00 | C_30_H_46_O |  |  | 423.3619  (0.25) | 69.0693,  81.0694,  95.0850,  109.1005,  121.1005,  147.1164 | - | - | - | - | - | + | (X.-H. Li, Feng, and Shi 2008) |
| 197 | Oleanane | **Oleanolic acid #**  **Isomer 3** | 30.14 | C_30_H_48_O_3_ | 455.3544  (-1.72) | 455.3551 |  |  | + | ++ | + | +++ | + | ++ | (Ayhan Ulubelen et al. 2000) |
| 198 | Oleanane | **Acetyl-b-boswellic acid** | 32.63 | C_32_H_50_O_4_ | 497.3623  (2.65) | 437.3411, 497.3629 |  |  | - | + | - | - | - | - | (Belsner et al. 2003) |
| 199 | Hopane and Moretane | **Mollugogenol A** | 33.80 | C_30_H_52_O_4_ | 475.3786  (1.75) | 57.0341,  397.3462,  415.3571,  457.3672, |  |  | - | + | - | - | - | - | (Hamburger et al. 1989) |
| 200 | Lupane | **Dihydrobetulinic acid** | 34.26 | C_30_H_50_O_3_ |  |  | 481.3652  [M + Na]^+^  (-0.14) | 57.0696,  89.0596,  463.3549, | - | + | - | - | - | **-** | (Ee et al. 2011) |
| 201 | Lupane | **Lupeone#**  **isomer 1** | 35.44 | C_30_H_48_O |  |  | 425.3771  (0.53) | 81.0694.  95.0852, 109.1007,  121.1008 | - | - | + | + | + | + | (Ayhan Ulubelen et al. 1998) |
| 202 | Lupane | **Lupeone #**  **isomer 2** | 39.35 | C_30_H_48_O |  |  | 425.3775  (1.02) | 81.0695,  95.0853,  109.1008,  123.1164,  135.1162 | - | -. | + | +++ | + | ++ | (Ayhan Ulubelen et al. 1998) |
|  | **Meroterpenoids** | | | | | | | | | | | | | | |
| 203 | Mero  terpenoid | **Dimethyl-6-(1-hydroxyethyl)chroman-4-one** | 23.59 | C_13_H_16_O_3_ |  |  | 221.1170  (0.26) | 57.0692,  91.0538,  105.0693,  137.0594 | + | + | ++ | ++ | - | + | (Bohlmann et al. 1981) |
| 204 | Mero  terpenoid | **α-tocospiros** | 36.70 | C_29_H_50_O_4_ |  |  | 463.3785  (-0.53) | 57.0696, 69.0695, 83.0852, 139.0750, 177.0904 | - | + | + | + | + | + | (Chiang and Kuo 2003) |
|  | **Miscellaneous** | | | | | | | | | | | | | | |
| 205 | Phenolics | **Hydroxy**  **acetophenone** | 7.33 | C_8_H_8_O_2_ | 135.0453  (-0.97) | 65.0026, 93.0333, 117.0333 |  |  | - | - | ++ | + | ++ | + | (Fraga et al. 2009) |
| 206 | Chromane | **(Dimethylallyl)- methoxy-methylchromone** | 22.16 | C1_6_H_18_O_3_ |  |  | 259.1330  (-0.25) | 145.0995,  158.1076,  185.0952,  229.0852 | - | + | - | - | - | - | (Bohlmann and Zdero 1982) |
|  | **Coumarins** | | | | | | | | | | | | | | |
| 207 | Coumarin | **Altissimacoumarin E** | 12.05 | C_21_H_28_O_8_ | 407.1705  (1.4) | 153.0549, 193.0860, 221.0817, 259.1327 |  |  | - | - | - | - | - | + | (Dao et al. 2012) |
| 208 | Coumarin | **Cycloisobrachycoumarinone epoxide** | 19.21 | C_20_H_22_O_5_ |  |  | 365.1357  [M + Na]^+^  (0.65) | 55.0536,  216.0765,  307.0938,  337.1403 | - | - | ++ | - | + | ++ | (Oketch-Rabah et al. 1997) |
| 209 | Coumarin | **3-{3-ethenyl-3,10-dimethyl-5-oxo-2h,4h-pyrano[3,2-c]chromen-2-yl}-2-methylprop-2-enoic acid** | 19.26 | C_20_H_20_O_5_ |  |  | 341.1381  (0.46) | 83.0488,  135.0800,  201.0905,  229.0854,  255.1367 | - | - | + | ++ | - | + | (Shukla et al. 1982) |
| 210 | Coumarin | **Schinilenol**  **Isomer 1** | 19.26 | C_20_H_24_O_5_ | 343.1551  (-0.28) | 97.0654,  285.1131,  300.1359,  315.1600,  327.1236,  343.1551 |  |  | - | - | + | + | ++ | + | (I.-S. Chen et al. 1995) |
| 211 | Coumarin | **3-{3-ethenyl-3,10-dimethyl-5-oxo-2h,4h-pyrano[3,2-c]chromen-2-yl}-2-methylprop-2-enoic acid** | 19.73 | C_20_H_20_O_5_ |  |  | 341.1378  (1.62) | 83.0485, 135.0798, 173.0955, 229.0854,  229.0854, 255.1368 | - | - | - | - | + | + | (Shukla et al. 1982) |
| 212 | Coumarin | **Cordatolide E** | 20.74 | C_20_H_24_O_6_ | 359.1503  (-0.69) | 247.0975,271.0978,272.1054,286.1210,287.1267,318.1789 |  |  | - | - | ++ | + | + | + | ( McKee et al. 1998) |
| 213 | Coumarin | **Schinilenol**  **Isomer 2** | 21.65 | C_20_H_24_O_5_ | 343.1552  (-0.23) | 231.1021,257.1173,285.1129,300.1358,315.1596,327.1237,343.1550 |  |  | - | - | - | + | - | - | (I.-S. Chen et al. 1995) |
| 215 | Coumarin | **Dimethoxy-2-oxochromen-8-yl)-3-methylbut-3-en-2-yl 3-methylbutanoate** | 21.81 | C_21_H_26_O_6_ |  |  | 397.1624  [M + Na]^+^  (-1.05) | 225.0877,  267.0986,  283.1293,  295.0935,  310.1167,  369.1668 | + | - | ++ | +++ | - | - | (KINOSHITA, WU, and HO 1996) |
| 216 | Coumarin | **Dimethyloxiran-2-yl)-3-methylpent-2-en-1-yl]oxy}-2H-chromen-2-one** | 22.77 | C_19_H_22_O_4_ |  |  | 315.1586  (1.44) | 93.0695,  105.0704,  157.0997,  189.0161,  223.0750 | - | + | - | - | - | - | (Wilzer et al. 1989) |
| 217 | Coumarin | **Suberosin** | 26.53 | C_15_H_16_O_3_ |  |  | 143.0853,  158.1075,  174.1036,  215.0685 | 245.1165  (2.59) | - | + | - | - | - | - | (Chang 1990) |
| 218 | Coumarin | **Gummosin** | 26.63 | C_24_H_30_O_4_ |  |  | 383.2213  **(**-0.23) | 95.0851,  105.0696,  119.0852,  163.0384 | - | + | - | - | - | - | (Iranshahi et al. 2010) |

**Supplementary Table S2**: Prediction of activity spectra for top-best docking result compounds from *Salvia* species (PASS analysis) according to Lipinski’s rule of five

| **No.** | **Compound name** | **Num. Rotatable Bonds** | **clog P (p < 5)** | **HBA** | **HBD** | **Molecular Weight g/mol** | **TPSA Å²** | **SAS** | **Passes Lipinski** |
| --- | --- | --- | --- | --- | --- | --- | --- | --- | --- |
| 1 | Luteolin *O*-glucuronide | 4 | -0.06 | 12 | 7 | 462.36 | 207.35 | 5.11 | No; 2 violations: NorO>10, NHorOH>5 |
| 2 | Hispidulin 7-glucoside | 5 | 0.37 | 11 | 6 | 462.4 | 179.28 | 5.31 | No; 2 violations: NorO>10, NHorOH>5 |
| 3 | Acacetin | 2 | 2.52 | 5 | 2 | 284.26 | 79.9 | 2.89 | Yes |
| 4 | Diosmetin | 2 | 2.19 | 6 | 3 | 300.26 | 100.13 | 3.03 | Yes |
| 5 | Apigenin | 1 | 2.11 | 5 | 3 | 270.24 | 90.9 | 2.96 | Yes |
| 6 | Apigenin 7-*O*-glucoside (Apigetrin) | 4 | 0.55 | 10 | 6 | 432.38 | 170.05 | 5.12 | Yes; 1 violation: NHorOH>5 |
| 7 | Apigenin dimethyl ether (Apigenin 7,4'-dimethyl ether) | 3 | 2.79 | 5 | 1 | 298.29 | 68.9 | 3.14 | Yes |
| 8 | Salvigenin | 4 | 2.88 | 6 | 1 | 328.32 | 78.13 | 3.37 | Yes |
| 9 | Cirsimaritin | 3 | 2.46 | 6 | 2 | 314.29 | 89.13 | 3.27 | Yes |
| 10 | Velutin | 3 | 2.6 | 6 | 2 | 314.29 | 89.13 | 3.12 | Yes |

**Supplementary Table S3**: Prediction of LD_50_, toxicity class and ADMET properties of top-best docking result compounds from *Salvia* species

| **No.** | **Compound name** | | **LD_50_ (mg/kg)** | **Toxicity class** | | **GI absorption** | **BBB permeant** | **Pgp substrate** | | **CYP1A2 inhibitor** | **CYP2C19 inhibitor** | | **CYP2C9 inhibitor** | | **CYP2D6 inhibitor** | | **CYP3A4 inhibitor** |
| --- | --- | --- | --- | --- | --- | --- | --- | --- | --- | --- | --- | --- | --- | --- | --- | --- | --- |
| **1** | Luteolin *O*-glucuronide | 5000 | | 5 | Low | | no | | yes | no | | no | no | no | | no | |
| **2** | Hispidulin 7-glucoside | 5000 | | 5 | Low | | no | | yes | no | | no | no | no | | yes | |
| **3** | Acacetin | 3919 | | 5 | high | | no | | no | yes | | no | yes | yes | | yes | |
| **4** | Diosmetin | 3919 | | 5 | High | | no | | no | yes | | no | yes | yes | | yes | |
| **5** | Apigenin | 2500 | | 5 | High | | no | | no | yes | | no | no | yes | | yes | |
| **6** | Apigenin 7-*O*-glucoside (Apigetrin) | 5000 | | 5 | Low | | no | | yes | no | | no | no | no | | no | |
| **7** | Apigenin dimethyl ether (Apigenin 7,4'-dimethyl ether) | 3919 | | 5 | high | | yes | | no | yes | | yes | yes | yes | | yes | |
| **8** | Salvigenin | 4000 | | 5 | high | | yes | | no | yes | | yes | yes | yes | | yes | |
| **9** | Cirsimaritin | 4000 | | 5 | High | | no | | no | yes | | no | yes | yes | | yes | |
| **10** | Velutin | 4000 | | 5 | high | | no | | no | yes | | no | yes | yes | | yes | |

**Supplementary Table S4.** Docking grid box coordinates and dimensions for target proteins

| **Protein** | **PDB ID** | **Grid center X** | **Grid center Y** | **Grid center Z** | **Grid size (Å³)** |
| --- | --- | --- | --- | --- | --- |
| EGFR | 1M17 | 27.714 | -0.108 | 56.936 | 68×64×82 |
| PPARG | 2HFP | 21.911 | -6.842 | 6.912 | 116×120×122 |
| AKR1C1 | 3C3U | 8.890 | -6.547 | 22.799 | 74×106×112 |
| SRC | 1A09 | 45.518 | 14.083 | 29.793 | 96×104×126 |
| AKR1C3 | 1RY0 | -3.816 | -18.073 | 11.825 | 100×122×126 |
| TNF | 7KBA | -9.460 | -0.334 | 13.033 | 104×82×126 |
| CASP3 | 1RHU | 76.258 | -16.456 | 103.532 | 84×70×86 |
| CASP8 | 1F9E | 42.709 | 75.593 | 13.961 | 126×94×102 |
| PARP1 | 2RCW | -22.049 | 66.308 | 32.173 | 112×84×104 |

**Supplementary Table S5.** Redocking validation results for the selected target proteins

| Target protein | PDB ID | Redocking RMSD (Å) |
| --- | --- | --- |
| EGFR | 1M17 | 1.3190 |
| PPARG | 2HFP | 1.8491 |
| AKR1C1 | 3C3U | 0.1750 |
| SRC | 1A09 | 1.4326 |
| AKR1C3 | 1RY0 | 1.8282 |
| CASP3 | 1RHU | 1.9440 |
| CASP8 | 1F9E | 1.8352 |
| PARP1 | 2RCW | 0.8874 |

**Supplementary Table S6:**  MM-PBSA binding free energy decomposition for cirsimaritin Bound to AKR1C3 and PARP1 Proteins

| Protein–Ligand System | ΔVDW (kcal/mol) | ΔEEL (kcal/mol) | ΔEGB (kcal/mol) | ΔESURF (kcal/mol) | ΔTOTAL (kcal/mol) |
| --- | --- | --- | --- | --- | --- |
| AKR1C3 | −41.30 ± 0.43 | −22.95 ± 1.06 | 40.78 ± 0.91 | −5.07 ± 0.03 | −28.73 ± 0.53 |
| PPARP1 | −20.92 ± 0.69 | −29.97 ± 1.83 | 41.12 ± 1.36 | −3.64 ± 0.09 | −16.88 ± 0.79 |

**Supplementary Figures**


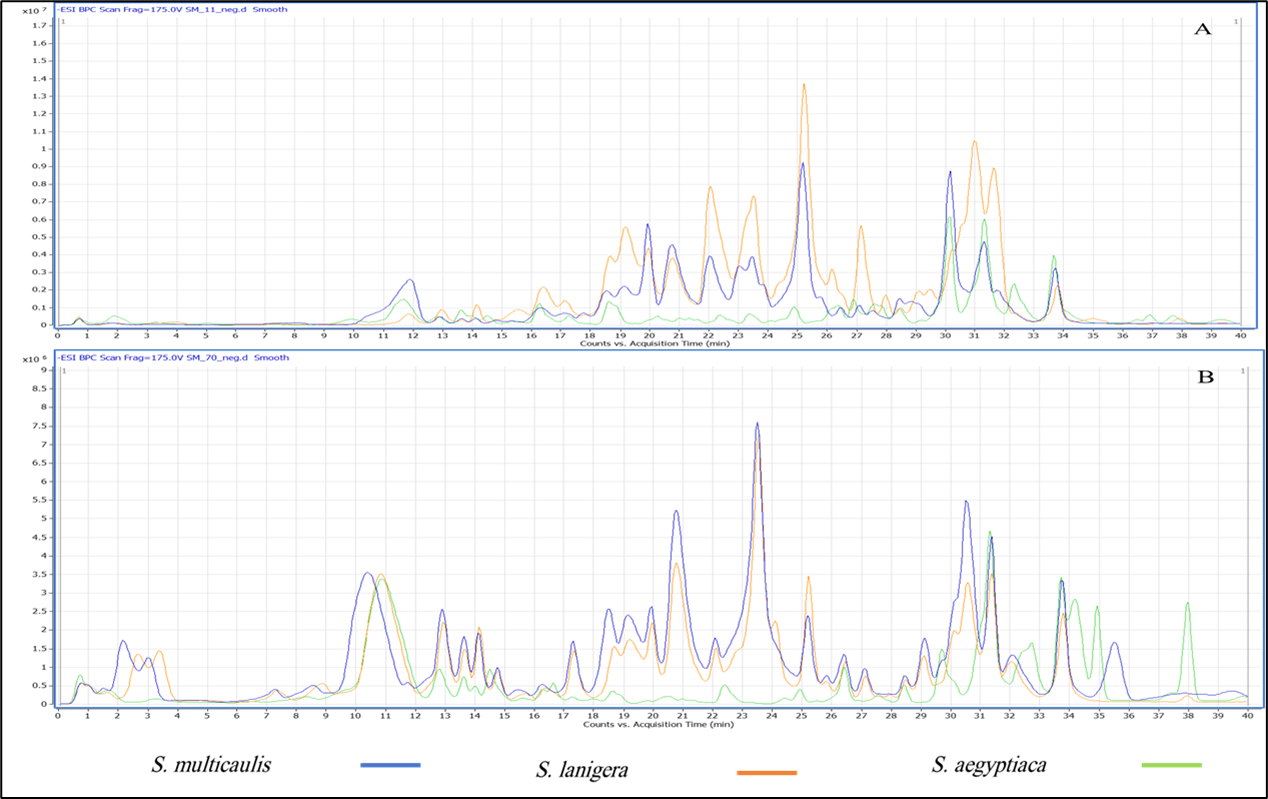


**Supplementary Figure S1:** Base peak chromatogram of the selected *Salvia* species extracted**: (A)** CH₂Cl₂/MeOH (1:1, v/v) and

**(B)** (MeOH/H₂O, 70:30, v/v) in negative ionization.


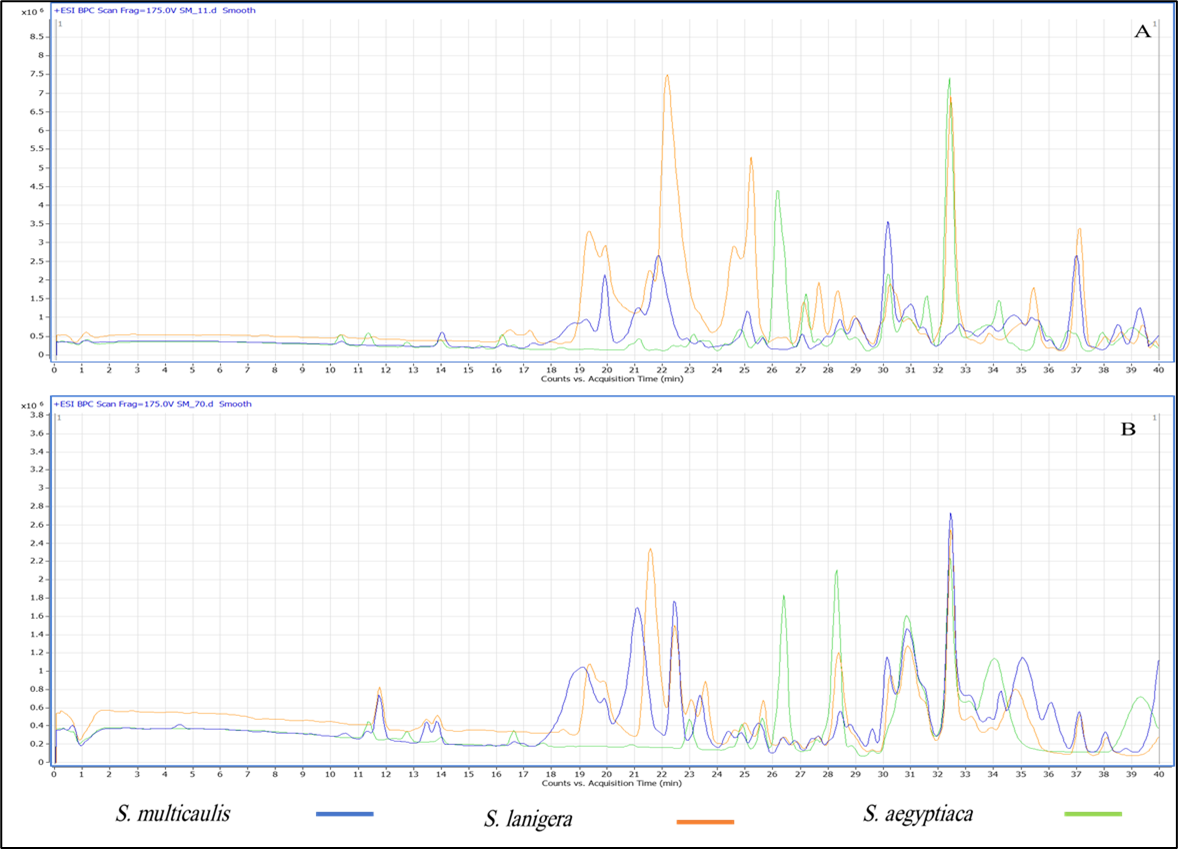


**Supplementary Figure S2:** Base peak chromatogram of the selected *Salvia* species extracted: (**A**) CH₂Cl₂/MeOH (1:1, v/v)

and (**B**) (MeOH/H₂O, 70:30, v/v) in positive ionization.

**
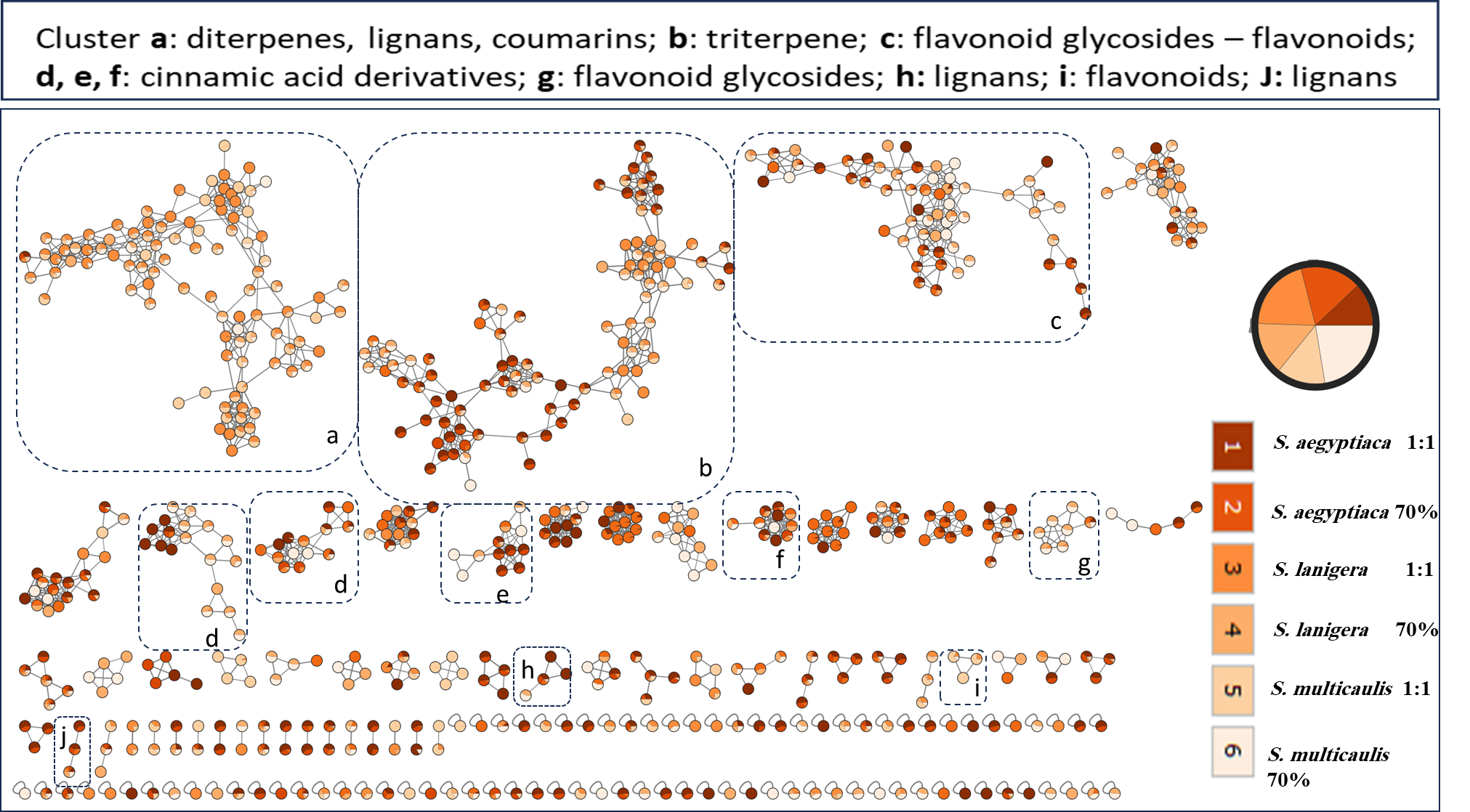
**

**Supplementary Figure S3**: Full FBMN constructed from the acquired UPLC-HRMS/MS data in the negative ionization mode of the leaves of selected *Salvia* species.


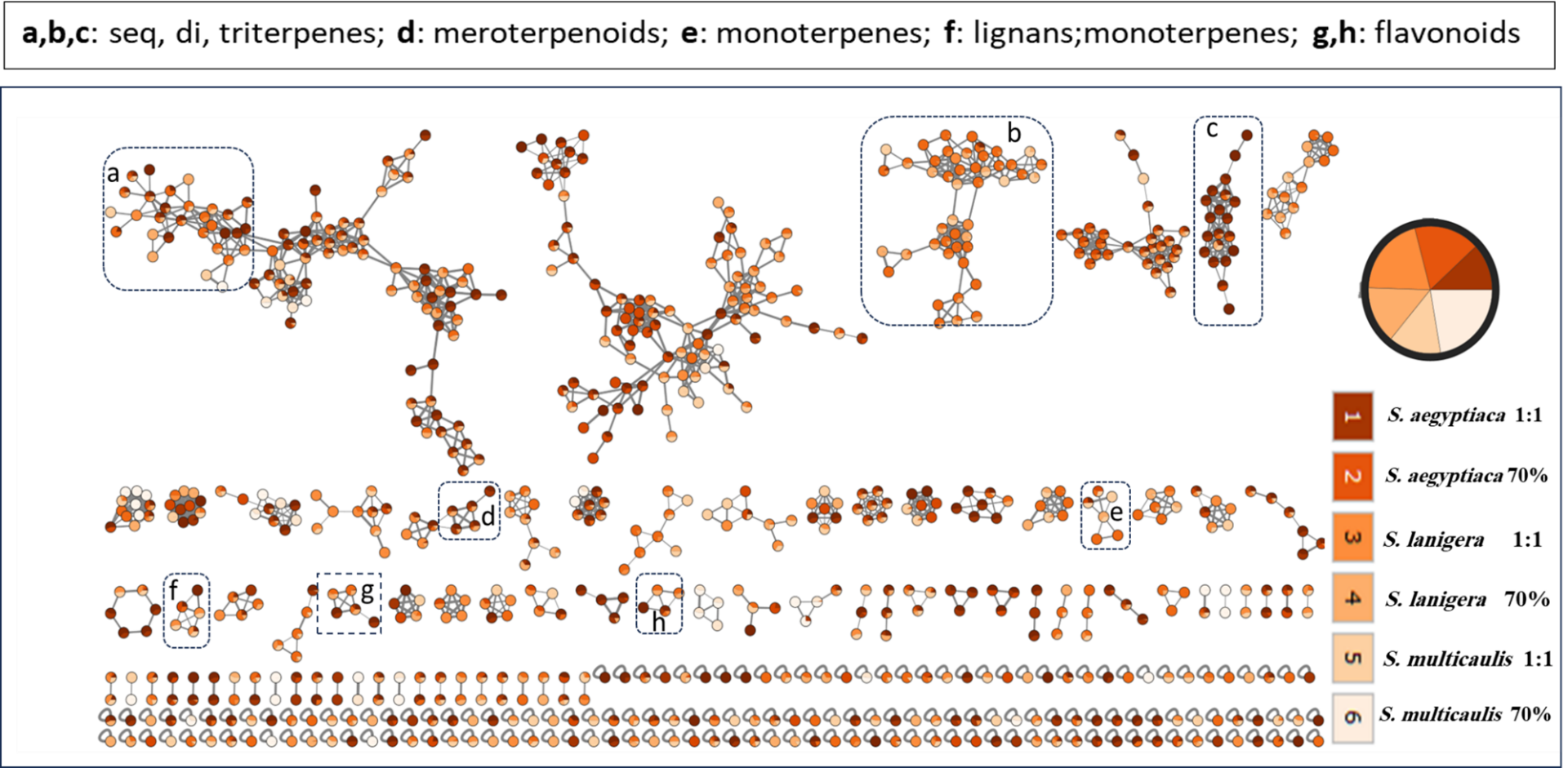


**Supplementary Figure S4:** Full FBMN constructed from the acquired UPLC-HRMS/MS data in the positive ionization mode of the leaves of selected *Salvia* species.


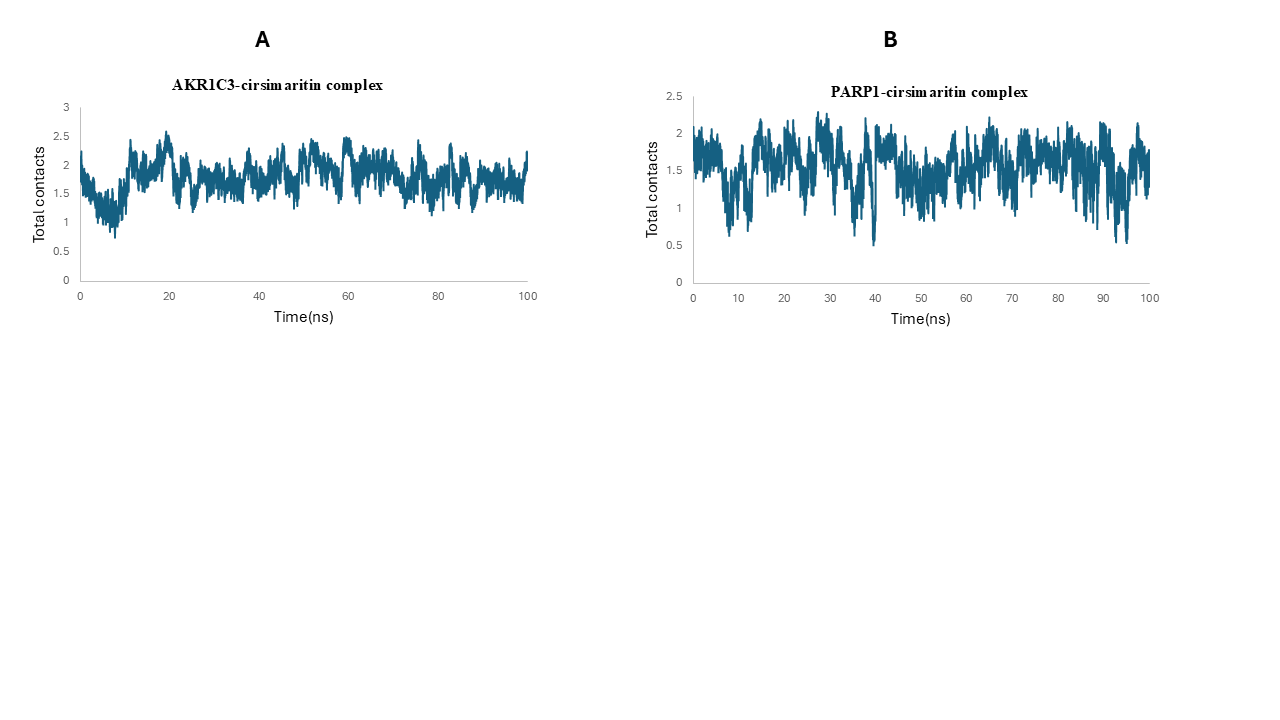


**Supplementary figure S5**. Total contact analysis for cirsimaritin complexes. (A) AKR1C3–cirsimaritin complex showing consistent contact profile.(B) PARP1–cirsimaritin complex exhibiting dynamic binding behavior, with fluctuating contacts. Line plots represent total contacts over 100 ns trajectories, highlighting differences in binding stability between AKR1C3 and PARP1 active sites.

**
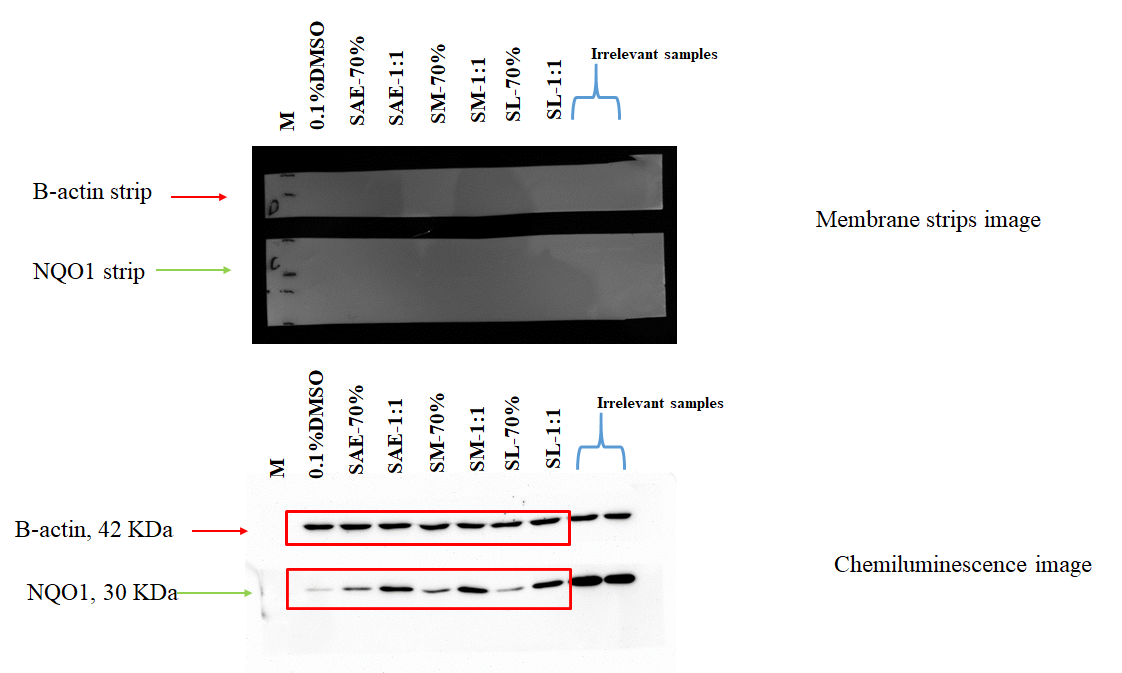
**

**Supplementary Figure S6:**Original uncropped western blot strips of NQO1 induction by different

*Salvia* extracts displayed in Fig.21 of the main manuscript. The upper panel is the light photograph of

membrane strips, whereas the bottom panel is the corresponding chemiluminescence image. Spliced

strips shown are belonging to the same whole blot. Hepa1c1c7 cells were cultured, treated and processed for Western blotting as mentioned at the Materials and Methods sections. Please note that the red boxed samples are the samples related to Fig. 21 of the current study, the unboxed 2 lanes are extra irrelevant samples run on the same gel, as labelled. We also displayed Fig.21 of the main manuscript below for direct illustration.

**
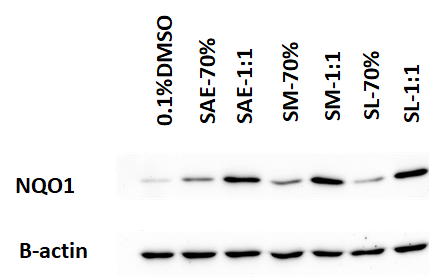
**

**Figure 21 of the main manuscript file**

**
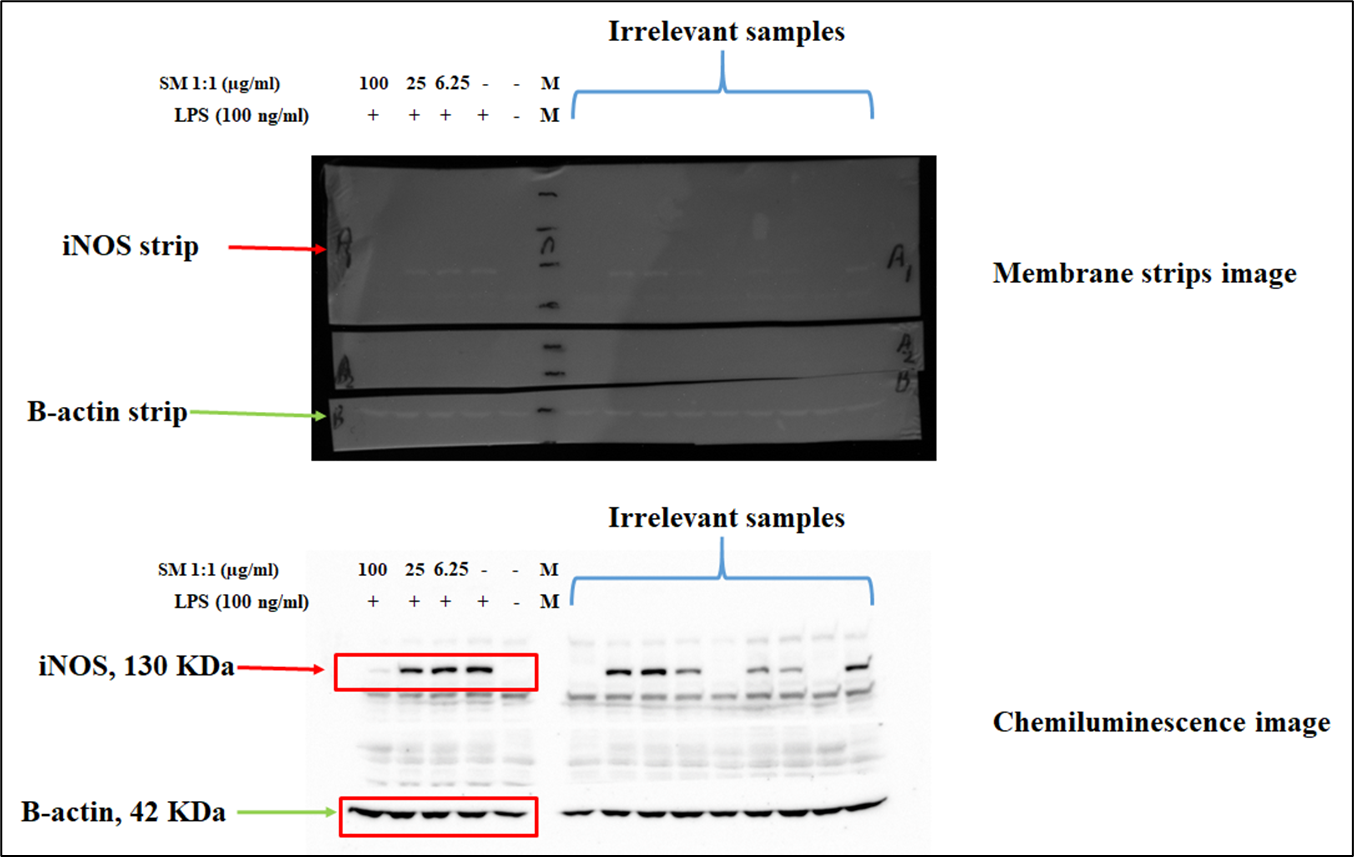
**

**Supplementary Figure S7:** Original, Uncropped and non-horizontally flipped photos of western blot strips of iNOS inhibition by SM 1:1 extract shown in Figure 22C of the manuscript. The upper panel is the light photograph of membrane strips taken before ECL imaging, whereas the bottom panel is the corresponding chemiluminescence image. Spliced strips shown are belonging to the same whole blot and the middle strip is unrelated to this current study but shown to reassemble the original whole blot as per the guidelines. RAW264.7 cells were cultured, treated and processed for Western blotting as mentioned at the Materials and Methods sections. Please note that the version displayed in the main manuscript is a horizontally flipped &cropped version of the original-unprocessed version of the strips so that the controls (LPS^-^, LPS^+^) followed by samples are shown left to right in the main manuscript and shown below as well. The horizontal flipping is a display option in the Visionworks LS software of the imager for final image display options. The molecular weight ladder bands (Lane **M**) are not normally visualized in the chemiluminescence version of the blot (bottom panel) as it does not react with chemiluminescence substrate (Not HRP-labelled), therefore this M lane appears band-free as shown, this is normal. The molecular weight marker was loaded on the middle of the gel as shown in the membrane strip image to separate between the study samples and another unrelated sample group run on the same gel, as labelled above.


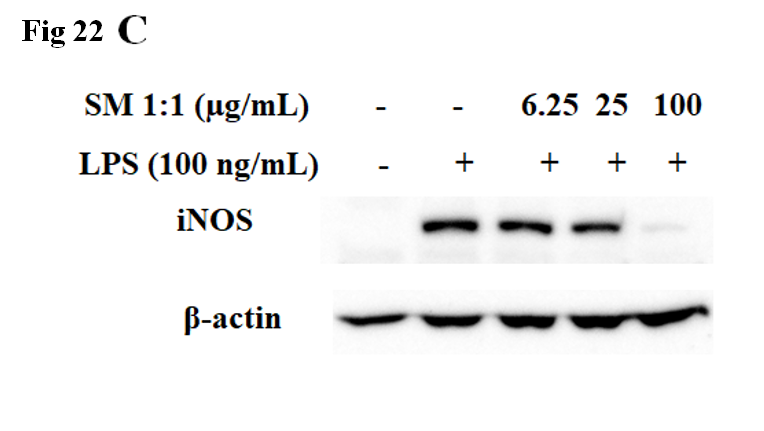


**Fig. 22 C (Horizontally-flipped and cropped from the original blot shown in the supplementary Figure S7 above.**

**References**

Abdalla, Mohamed F, Nabiel A M Saleh, Said Gabr, Ahmed M Abu-Eyta, and Hani El-Said. 1983. “Flavone Glycosides of Salvia Triloba.” *Phytochemistry* 22 (9): 2057–60.

Acebey-Castellon, Ivone Lucia, Laurence Voutquenne-Nazabadioko, Huong Doan Thi Mai, Nathalie Roseau, Naima Bouthagane, Dima Muhammad, Elisabeth Le Magrex Debar, Sophie C Gangloff, Marc Litaudon, and Thierry Sevenet. 2011. “Triterpenoid Saponins from Symplocos Lancifolia.” *Journal of Natural Products* 74 (2): 163–68.

Adezet, T, S Canigueral, J Iglesias, and D Manso. 1986. “Some Flavonoid Compounds of Salvia Candelabrum.” *Planta Medica* 52 (06): 525–26.

Ahmad, V U, M Zahid, M S Ali, S Ahmad, Z Ali, M Z Iqbal, and R B Tareen. 2000. “Buchariate: An Aromatic Ester from Salvia Bucharica.” *Scientia Pharmaceutica*. https://doi.org/10.3797/scipharm.aut-00-39.

Ahmad, Viqar Uddin, Shaheen Bano, and Nasreen Bano. 1986. “A Triterpene Acid from Nepeta Hindostana.” *Phytochemistry* 25 (6): 1487–88.

Ahmed, A A, J Jakupovic, F Eid, and A A Ali. 1988. “11-Hydroxyjasionone, a New Sesquiterpene Type from Jasonia Montana.” *Phytochemistry* 27 (12): 3875–77. https://doi.org/https://doi.org/10.1016/0031-9422(88)83035-8.

Ahsan, Monira, James A Armstrong, Alexander I Gray, and Peter G Waterman. 1995. “Terpenoids, Alkaloids and Coumarins from Boronia Inornata and Boronia Gracilipes.” *Phytochemistry* 38 (5): 1275–78. https://doi.org/https://doi.org/10.1016/0031-9422(94)00567-D.

Alipieva, Kalina I, Emanuela P Kostadinova, Ljuba N Evstatieva, Marina Stefova, and Vassya S Bankova. 2009. “An Iridoid and a Flavonoid from Sideritis Lanata L.” *Fitoterapia* 80 (1): 51–53.

Altinier, Gianmario, Silvio Sosa, Rita P Aquino, Teresa Mencherini, Roberto Della Loggia, and Aurelia Tubaro. 2007. “Characterization of Topical Antiinflammatory Compounds in Rosmarinus Officinalis L.” *Journal of Agricultural and Food Chemistry* 55 (5): 1718–23. https://doi.org/10.1021/jf062610+.

Amaro-Luis, J Manuel, J Ramón Herrera, and Javier G Luis. 1998. “Abietane Diterpenoids from Salvia Chinopeplica.” *Phytochemistry* 47 (5): 895–97.

Ang, Hooi Hoon, Yukio Hitotsuyanagi, Haruhiko Fukaya, and Koichi Takeya. 2002. “Quassinoids from Eurycoma Longifolia.” *Phytochemistry* 59 (8): 833–37. https://doi.org/https://doi.org/10.1016/S0031-9422(01)00480-0.

Ang, Hooi Hoon, Yukio Hitotsuyanagi, and Koichi Takeya. 2000. “Eurycolactones A–C, Novel Quassinoids from Eurycoma Longifolia.” *Tetrahedron Letters* 41 (35): 6849–53.

Ankli, Anita, Jörg Heilmann, Michael Heinrich, and Otto Sticher. 2000. “Cytotoxic Cardenolides and Antibacterial Terpenoids from Crossopetalum Gaumeri.” *Phytochemistry* 54 (5): 531–37.

Aoki, Shunji, Mami Sanagawa, Yasuo Watanabe, Andi Setiawan, Masayoshi Arai, and Motomasa Kobayashi. 2007. “Novel Isomarabarican Triterpenes, Exhibiting Selective Anti-Proliferative Activity against Vascular Endothelial Cells, from Marine Sponge Rhabdastrella Globostellata.” *Bioorganic & Medicinal Chemistry* 15 (14): 4818–28. https://doi.org/https://doi.org/10.1016/j.bmc.2007.04.070.

Aranganathan, S, J Panneer Selvam, N Sangeetha, and N Nalini. 2009. “Modulatory Efficacy of Hesperetin (Citrus Flavanone) on Xenobiotic-Metabolizing Enzymes during 1, 2-Dimethylhydrazine-Induced Colon Carcinogenesis.” *Chemico-Biological Interactions* 180 (2): 254–61.

Argyropoulou, Catherine, Anastasia Karioti, and Helen Skaltsa. 2009. “Labdane Diterpenes from Marrubium Thessalum.” *Phytochemistry* 70 (5): 635–40. https://doi.org/https://doi.org/10.1016/j.phytochem.2009.03.011.

Ashurst, P R, F Bohlmann, L Farkas, Y Gaoni, H Kling, R Mechoulam, G A Morrison, L Pallos, J Romo, and A Romo De Vivar. 1967. “Natürlich Vorkommende Auronglykoside.” *Fortschritte Der Chemie Organischer Naturstoffe/Progress in the Chemistry of Organic Natural Products/Progrès Dans La Chimie Des Substances Organiques Naturelles*, 150–74.

Awang, Khalijah, Zunoliza Abdullah, Mat Ropi Mukhtar, Marc Litaudon, Faridahanim Mohd. Jaafar, A Hamind A. Hadi, and Noel F Thomas. 2009. “Dunaliine A, a New Amino Diketone from Desmos Dunalii (Annonaceae).” *Natural Product Research* 23 (7): 652–58.

Banno, Norihiro, Toshihiro Akihisa, Harukuni Tokuda, Ken Yasukawa, Hiroshi Higashihara, Motohiko Ukiya, Kenji Watanabe, Yumiko Kimura, Jun-ichi Hasegawa, and Hoyoku Nishino. 2004. “Triterpene Acids from the Leaves of Perilla Frutescens and Their Anti-Inflammatory and Antitumor-Promoting Effects.” *Bioscience, Biotechnology, and Biochemistry* 68 (1): 85–90.

Barua, A K, M K Chowdhury, S Biswas, C Das Gupta, S K Banerjee, S K Saha, A Patra, and A K Mitra. 1985. “The Structure and Stereochemistry of Phlogantholide-A, a Diterpene from Phlogacanthus Thyrsiflorus.” *Phytochemistry* 24 (9): 2037–39.

Belsner, Klaus, Berthold Büchele, Udo Werz, Tatiana Syrovets, and Thomas Simmet. 2003. “Structural Analysis of Pentacyclic Triterpenes from the Gum Resin of Boswellia Serrata by NMR Spectroscopy.” *Magnetic Resonance in Chemistry* 41 (2): 115–22.

Bi, Xueyan, Wei Li, Tatsunori Sasaki, Qin Li, Naoko Mitsuhata, Yoshihisa Asada, Qingbo Zhang, and Kazuo Koike. 2011. “Secoiridoid Glucosides and Related Compounds from Syringa Reticulata and Their Antioxidant Activities.” *Bioorganic & Medicinal Chemistry Letters* 21 (21): 6426–29.

Bisio, Angela, Nadia Fontana, Giovanni Romussi, Giovanni Ciarallo, Nunziatina De Tommasi, Cosimo Pizza, and Angelo Mugnoli. 1999. “Clerodane Diterpenoids from Salvia Blepharophylla.” *Phytochemistry* 52 (8): 1535–40.

Boalino, Dionne M, Stewart McLean, William F Reynolds, and Winston F Tinto. 2004. “Labdane Diterpenes of Leonurus s Ibiricus.” *Journal of Natural Products* 67 (4): 714–17.

Bohlmann, Ferdinand, Naleen Borthakur, Harold Robinson, and Robert M King. 1982. “Eudesmane Derivatives from Epaltes Brasiliensis.” *Phytochemistry* 21 (7): 1795–97.

Bohlmann, Ferdinand, and Rajinder K Gupta. 1981. “Guaianolides from Centaurea Canariensis.” *Phytochemistry* 20 (12): 2773–75.

Bohlmann, Ferdinand, Pahup Singh, Christa Zdero, Annette Ruhe, Robert M King, and Harold Robinson. 1982. “Furanoheliangolides from Two Eremanthus Species and from Chresta Sphaerocephala.” *Phytochemistry* 21 (7): 1669–73.

Bohlmann, Ferdinand, and Christa Zdero. 1982. “Glaucolides and Other Constituents from South African Vernonia Species.” *Phytochemistry* 21 (9): 2263–67. https://doi.org/https://doi.org/10.1016/0031-9422(82)85189-3.

Bohlmann, Ferdinand, Christa Zdero, and Maniruddin Ahmed. 1982. “New Sesquiterpene Lactones, Geranyllinalol Derivatives and Other Constituents from Geigeria Species.” *Phytochemistry* 21 (7): 1679–91.

Bohlmann, Ferdinand, Christa Zdero, Robert M King, and Harold Robinson. 1985. “Further Germacranolides from Eupatorium Serotinum.” *Planta Medica* 51 (01): 76–77.

Bohlmann, Ferdinand, Christa Zdero, Joachim Pickard, Harold Robinson, and Robert M King. 1981. “New Types of Sesquiterpene Lactones and Other Constituents from Trichogonia Species.” *Phytochemistry* 20 (6): 1323–33.

Bohlmann, Ferdinand, Christa Zdero, Harold Robinson, and Robert M King. 1980. “Caryophyllene Derivatives and a Heliangolide from Lychnophora Species.” *Phytochemistry* 19 (11): 2381–85. https://doi.org/https://doi.org/10.1016/S0031-9422(00)91032-X.

Chabani, Sonia, Hamada Haba, Catherine Lavaud, Mohammed Benkhaled, and Dominique Harakat. 2013. “Flavonoid Glycosides and Triterpenoids from Atractylis Flava.” *Phytochemistry Letters* 6 (1): 9–13.

Chang, Shih-Hsien. 1990. “Flavonoids, Coumarins and Acridone Alkaloids from the Root Bark of Citrus Limonia.” *Phytochemistry* 29 (1): 351–53. https://doi.org/https://doi.org/10.1016/0031-9422(90)89075-K.

Chávez, Haydee, Gladis Rodrı́guez, Ana Estévez-Braun, Ángel G Ravelo, Rafael Estévez-Reyes, Antonio G González, Jose L Fdez-Puente, and Dolores Garcı́a-Grávalos. 2000. “Macrocarpins A–D, New Cytotoxic nor-Triterpenes from Maytenus Macrocarpa.” *Bioorganic & Medicinal Chemistry Letters* 10 (8): 759–62.

Chen, Ih-Sheng, Yuh-Chwen Lin, Ian-Lih Tsai, Che-Ming Teng, Feng-Nien Ko, Tsutomu Ishikawa, and Hisashi Ishii. 1995. “Coumarins and Anti-Platelet Aggregation Constituents from Zanthoxylum Schinifolium.” *Phytochemistry* 39 (5): 1091–97.

Chen, Jih-Jung, Hsinn-Hsing Lee, Cheng-Dean Shih, Chang-Hui Liao, Ih-Sheng Chen, and Tsung-Hsien Chou. 2007. “New Dihydrochalcones and Anti-Platelet Aggregation Constituents from the Leaves of Muntingia Calabura.” *Planta Medica* 73 (06): 572–77.

Chen, Li‐Xia, Feng Qiu, Hong Wei, Ge‐Xia Qu, and Xin‐Sheng Yao. 2006. “Nine New Ent‐Labdane Diterpenoids from the Aerial Parts of Andrographis Paniculata.” *Helvetica Chimica Acta* 89 (11): 2654–64.

Cheng, Xiangrong, Qi Zeng, Jie Ren, Jiangjiang Qin, Shoude Zhang, Yunheng Shen, Jiaxian Zhu, Fei Zhang, Ruijie Chang, and Yan Zhu. 2011. “Sesquiterpene Lactones from Inula Falconeri, a Plant Endemic to the Himalayas, as Potential Anti-Inflammatory Agents.” *European Journal of Medicinal Chemistry* 46 (11): 5408–15.

Chiang, Yi-Ming, and Yueh-Hsiung Kuo. 2003. “Two Novel α-Tocopheroids from the Aerial Roots of Ficus Microcarpa.” *Tetrahedron Letters* 44 (27): 5125–28.

Choi, Jae, Won Woo, Han Young, and Jong Park. 1990. “Phytochemical Study on Prunus Davidiana.” *Archives of Pharmacal Research* 13 (4).

Cui, Baoliang, You Hui Lee, Heebyung Chai, John C Tucker, Craig R Fairchild, Carmen Raventos-Suarez, Byron Long, Kate E Lane, Ana T Menendez, and Christopher W W Beecher. 1999. “Cytotoxic Sesquiterpenoids from Ratibida c Olumnifera.” *Journal of Natural Products* 62 (11): 1545–50.

Dao, Trong-Tuan, Tien-Lam Tran, Jayeon Kim, Phi-Hung Nguyen, Eun-Hee Lee, Junsoo Park, Ik-Soon Jang, and Won-Keun Oh. 2012. “Terpenylated Coumarins as SIRT1 Activators Isolated from Ailanthus Altissima.” *Journal of Natural Products* 75 (7): 1332–38.

Delgado, Guillermo, Jullo Hernández, and Rogelio Pereda-Miranda. 1989. “Triterpenoid Acids from Cunila Lythrifolia.” *Phytochemistry* 28 (5): 1483–85.

Dharmaratne, H Ranjith W, J R D Mayuri Sajeevani, Gishanthi P K Marasinghe, and EMHG Shantha Ekanayake. 1998. “Distribution of Pyranocoumarins in Calophyllum Cordato-Oblongum.” *Phytochemistry* 49 (4): 995–98.

Ee, Gwendoline Cheng Lian, Siau Hui Mah, Mawardi Rahmani, Yun Hin Taufiq-Yap, Soek Sin Teh, and Yang Mooi Lim. 2011. “A New Furanoxanthone from the Stem Bark of Calophyllum Inophyllum.” *Journal of Asian Natural Products Research* 13 (10): 956–60.

El-Askary, Hesham I, Meselhy R Meselhy, and Ahmed M Galal. 2003. “Sesquiterpenes from Cymbopogon Proximus.” *Molecules* 8 (9): 670–77.

Esquivel, Baldomero, Luis Manuel Hernández, Jorge Cárdenas, T P Ramamoorthy, and Lydia Rodríguez-Hahn. 1989. “Further Ent-Clerodane Diterpenoids from Salvia Melissodora.” *Phytochemistry* 28 (2): 561–66.

Farooq, Umar, Afsar Khan, Viqar Uddin Ahmad, Saleha Suleman Khan, Farzana Kousar, and and Saima Arshad. 2007. “Two New Rare-Class Tetracyclic Diterpenoids from *Otostegia Limbata*.” *Chemical and Pharmaceutical Bulletin* 55 (3): 471–73. https://doi.org/10.1248/cpb.55.471.

Fei, Dong-Qing, Shi-Gang Li, Chun-Mei Liu, Gang Wu, and Kun Gao. 2007. “Eremophilane-Type Sesquiterpene Derivatives from the Roots of Ligularia Lapathifolia.” *Journal of Natural Products* 70 (2): 241–45. https://doi.org/10.1021/np060304k.

Fiorentino, Antonio, Marina DellaGreca, Brigida D’Abrosca, Annunziata Golino, Severina Pacifico, Angelina Izzo, and Pietro Monaco. 2006. “Unusual Sesquiterpene Glucosides from Amaranthus Retroflexus.” *Tetrahedron* 62 (38): 8952–58.

Fiorentino, Antonio, Andreina Ricci, Brigida D’Abrosca, Severina Pacifico, Annunziata Golino, Marianna Letizia, Simona Piccolella, and Pietro Monaco. 2008. “Potential Food Additives from Carex Distachya Roots: Identification and in Vitro Antioxidant Properties.” *Journal of Agricultural and Food Chemistry* 56 (17): 8218–25.

Flores-Bocanegra, Laura, Martin González-Andrade, Robert Bye, Edelmira Linares, and Rachel Mata. 2017. “α-Glucosidase Inhibitors from Salvia Circinata.” *Journal of Natural Products* 80 (5): 1584–93.

Fraga, Braulio M, Carmen E Díaz, Ana Guadaño, and Azucena González-Coloma. 2005. “Diterpenes from Salvia Diterpenes from Salvia Broussonetii Transformed Roots and Their Insecticidal Activity Transformed Roots and Their Insecticidal Activity.” *Journal of Agricultural and Food Chemistry* 53 (13): 5200–5206.

Fraga, Braulio M, Melchor G Hernández, Concepción Fernández, and José M H Santana. 2009. “A Chemotaxonomic Study of Nine Canarian Sideritis Species.” *Phytochemistry* 70 (8): 1038–48.

Fronza, M, R Murillo, S Ślusarczyk, M Adams, M Hamburger, B Heinzmann, S Laufer, and I Merfort. 2011. “In Vitro Cytotoxic Activity of Abietane Diterpenes from Peltodon Longipes as Well as Salvia Miltiorrhiza and Salvia Sahendica.” *Bioorganic & Medicinal Chemistry* 19 (16): 4876–81. https://doi.org/https://doi.org/10.1016/j.bmc.2011.06.067.

Garbarino, Juan A, María C Chamy, and Vicente Gambaro. 1986. “Labdane Diterpenoids from Nolana Rostrata.” *Phytochemistry* 25 (12): 2833–36.

González, Antonio G, Lucia S Andrés, Juan R Herrera, Javier G Luis, and Angel G Ravelo. 1989. “Abietane Diterpenes with Antibiotic Activity from the Flowers of Salviacanariensis. Reaction of Galdosol with Diazomethane.” *Canadian Journal of Chemistry* 67 (2): 208–12. https://doi.org/10.1139/v89-035.

Grandolini, G, C G Casinovi, P Barbetti, and G Fardella. 1987. “A New Neoquassin Derivative from Quassia Amara.” *Phytochemistry* 26 (11): 3085–87. https://doi.org/https://doi.org/10.1016/S0031-9422(00)84604-X.

Gujer, René, Daniéle Magnolato, and Ron Self. 1986. “Glucosylated Flavonoids and Other Phenolic Compounds from Sorghum.” *Phytochemistry* 25 (6): 1431–36.

Gupta, Prasoon, Dinesh Kumar Yadav, Kiran Babu Siripurapu, Guatam Palit, and Rakesh Maurya. 2007. “Constituents of Ocimum Sanctum with Antistress Activity.” *Journal of Natural Products* 70 (9): 1410–16.

Hamburger, Matthias, Guy Dudan, A G Ramachandran Nair, R Jayaprakasam, and Kurt Hostettmann. 1989. “An Antifungal Triterpenoid from Mollugo Pentaphylla.” *Phytochemistry* 28 (6): 1767–68.

He, Hong‐Ping, Yue‐Mao Shen, Guo‐Ying Zuo, Xiao‐Sheng Yang, and Xiao‐Jiang Hao. 2003. “Dinorditerpene, Diterpenes, Alkaloids, and Coumarins from Clausena Dunniana.” *Helvetica Chimica Acta* 86 (9): 3187–93.

He, Zhen-Dan, Paul Pui-Hay But, Tak-Wah Dominic Chan, Hui DONG, Hong-Xi XU, Ching-Po LAU, and Han-Dong Sun. 2001. “Antioxidative Glucosides from the Fruits of Ligustrum Lucidum.” *Chemical and Pharmaceutical Bulletin* 49 (6): 780–84.

Hernández, Mireya, Baldomero Esquivel, Jorge Cárdenas, Lydia Rodríguez-Hahn, and T P Ramamoorthy. 1987. “Diterpenoid Abietane Quinones Isolated from Salvia Regla.” *Phytochemistry* 26 (12): 3297–99. https://doi.org/https://doi.org/10.1016/S0031-9422(00)82491-7.

Herz, Werner, and Palaniappan Kulanthaivel. 1984. “Ent-Pimaranes, Ent-Kauranes, Heliangolides and Other Constituents of Three Helianthus Species.” *Phytochemistry* 23 (7): 1453–59.

HEYMANN, Henry, Yasuhiro TEZUKA, Tohru KIKUCHI, and Sutardjo SUPRIYATNA. 1994. “Constituents of Sindora Sumatrana MIQ. II. Five New Sesquiterpenoids from the Dried Pods.” *Chemical and Pharmaceutical Bulletin* 42 (4): 941–46.

Ibrahim, Lamyaa F, Waled M El-Senousy, and Usama W Hawas. 2007. “NMR Spectral Analysis of Flavonoids from Chrysanthemum Coronarium.” *Chemistry of Natural Compounds* 43: 659–62.

Inoue, Toshio, Yukio Sugimoto, Hideki Masuda, and Chiaki Kamei. 2002. “Antiallergic Effect of Flavonoid Glycosides Obtained from Mentha Piperita L.” *Biological and Pharmaceutical Bulletin* 25 (2): 256–59.

Iranshahi, Mehrdad, Milena Masullo, Ali Asili, Ali Hamedzadeh, Bentolhoda Jahanbin, Michela Festa, Anna Capasso, and Sonia Piacente. 2010. “Sesquiterpene Coumarins from Ferula Gumosa.” *Journal of Natural Products* 73 (11): 1958–62. https://doi.org/10.1021/np100487j.

Janicsák, Gábor, Judit Hohmann, István Zupkó, Peter Forgo, Dóra Rédei, György Falkay, and Imre Máthé. 2003. “Diterpenes from the Aerial Parts of Salvia Candelabrum and Their Protective Effects against Lipid Peroxidation.” *Planta Medica* 69 (12): 1156–59.

Karioti, Anastasia, Anastasia Protopappa, Nikolaos Megoulas, and Helen Skaltsa. 2007. “Identification of Tyrosinase Inhibitors from Marrubium Velutinum and Marrubium Cylleneum.” *Bioorganic & Medicinal Chemistry* 15 (7): 2708–14.

Karioti, Anastasia, Helen Skaltsa, Jörg Heilmann, and Otto Sticher. 2003. “Acylated Flavonoid and Phenylethanoid Glycosides from Marrubium Velutinum.” *Phytochemistry* 64 (2): 655–60.

KINOSHITA, Takeshi, Jin-Bin WU, and Feng-Chi HO. 1996. “Prenylcoumarins from Murraya Paniculata Var. Omphalocarpa (Rutaceae): The Absolute Configuration of Sibiricin, Mexoticin and Omphamurin.” *Chemical and Pharmaceutical Bulletin* 44 (6): 1208–11.

Kir’yalov, N P, I A Murav’ev, E Fo Stepanova, and V F Bogatkina. 1970. “Triterpene Compounds of the Herbage of Glycyrrhiza Glabra.” *Chemistry of Natural Compounds* 6 (6): 787.

Kirimer, NEŞE, TEMEL Özek, K H C Baser, and M Harmandar. 1993. “The Essential Oil of Micromeria Fruticosa (L.) Druce Subsp. Serpyllifolia (Bieb.) PH Davis.” *Journal of Essential Oil Research* 5 (2): 199–200.

Kitajima, Mariko, Norie Fujii, Fumie Yoshino, Hiroshi Sudo, Kazuki Saito, Norio Aimi, and Hiromitsu Takayama. 2005. “Camptothecins and Two New Monoterpene Glucosides from Ophiorrhiza Liukiuensis.” *Chemical and Pharmaceutical Bulletin* 53 (10): 1355–58.

KOHDA, Hiroshi, Osamu TAKEDA, Seiji TANAKA, Kazuo YAMASAKI, Atsushi YAMASHITA, Tomonori KUROKAWA, and Sadahiko ISHIBASHI. 1989. “Isolation of Inhibitors of Adenylate Cyclase from Dan-Shen, the Root of Salvia Miltiorrhiza.” *Chemical and Pharmaceutical Bulletin* 37 (5): 1287–90.

Koyama, Yuka, Katsuyoshi Matsunami, Hideaki Otsuka, Takakazu Shinzato, and Yoshio Takeda. 2010. “Microtropiosides A–F: Ent-Labdane Diterpenoid Glucosides from the Leaves of Microtropis Japonica (Celastraceae).” *Phytochemistry* 71 (5–6): 675–81.

Krasteva, Ilina, Stefan Nikolov, Maki Kaloga, and Gisela Mayer. 2006. “Triterpenoid Saponins from Astragalus Corniculatus,” Zeitschrift für Naturforschung B, 61 (9): 1166–69. https://doi.org/doi:10.1515/znb-2006-0919.

———. 2007. “A New Saponin Lactone from Astragalus Corniculatus.” *Natural Product Research* 21 (10): 941–45.

Kraus, Christine, and Gerhard Spiteller. 1997. “Comparison of Phenolic Compounds from Galls and Shoots of Picea Glauca.” *Phytochemistry* 44 (1): 59–67.

Kuang, Hai-Xue, Ryoji Kasai, Kazuhiro Ohtani, Zhong-Shen LIU, Chun-Sheng YUAN, and Osamu TANAKA. 1989. “Chemical Constituents of Pericarps of Rosa Davurica Pall., a Traditional Chinese Medicine.” *Chemical and Pharmaceutical Bulletin* 37 (8): 2232–33.

Kuo, Yueh‐Hsiung, Shu‐Mei Lee, and Jeng‐Shiow Lai. 2000. “Constituents of the Whole Herb of Clinoponium Laxiflorum.” *Journal of the Chinese Chemical Society* 47 (1): 241–46.

Kurobayashi, Yoshiko, Hidemasa Sakakibara, Tetsuya Yanai, Izumi Yajima, and Kazuo Hayashi. 1991. “Volatile Flavor Compounds of Myoga (Zingiber Miogd).” *Agricultural and Biological Chemistry* 55 (6): 1655–57.

Kuroyanagi, Masanori, Takahiro Seki, Tatsuo Hayashi, Yoshio Nagashima, Nobuo Kawahara, Setsuko Sekita, and Motoyoshi Satake. 2001. “Anti-Androgenic Triterpenoids from the Brazilian Medicinal Plant, Cordia Multispicata.” *Chemical and Pharmaceutical Bulletin* 49 (8): 954–57.

Lapteva, K I, N Ya Tyukavkina, and L I Ryzhova. 1971. “Lignan Compounds from the Wood of Larix Dahurica and L. Sibirica.” *Chemistry of Natural Compounds* 7 (6): 802–3.

Lee, Dae-Young, Lakoon Jung, Ji-Hae Park, Ki-Hyun Yoo, In-Sik Chung, and Nam-In Baek. 2010. “Cytotoxic Triterpenoids from Cornus Kousa Fruits.” *Chemistry of Natural Compounds* 46: 142–45.

Lee, Kyung-Tae, Il-Cheol Sohn, Hee-Juhn Park, Dong-Wook Kim, Geun-Ok Jung, and Kun-Young Park. 2000. “Essential Moiety for Antimutagenic and Cytotoxic Activity of Hederagenin Monodesmosides and Bisdesmosides Isolated from the Stem Bark of Kalopanax Pictus.” *Planta Medica* 66 (04): 329–32.

Lee, Tzong-Huei, Shoei-Sheng Lee, Yuh-Chi Kuo, and Chang-Hung Chou. 2001. “Monoterpene Glycosides and Triterpene Acids from Eriobotrya d Eflexa.” *Journal of Natural Products* 64 (7): 865–69.

Li, Kunhua, Hongquan Duan, Kazuyoshi Kawazoe, and Yoshihisa Takaishi. 1997. “Terpenoids from Tripterygium Wilfordii.” *Phytochemistry* 45 (4): 791–96. https://doi.org/https://doi.org/10.1016/S0031-9422(97)00048-4.

Li, Wei, Hongwei Fu, Hong Bai, Tatsunori Sasaki, Hiroyoshi Kato, and Kazuo Koike. 2009. “Triterpenoid Saponins from Rubus Ellipticus Var. Obcordatus.” *Journal of Natural Products* 72 (10): 1755–60.

Li, Xue-Hu, Jia-Tao Feng, and Yan-Ping Shi. 2008. “Triterpenoids from Saussurea Ussuriensis.” *Canadian Journal of Chemistry* 86 (4): 281–84. https://doi.org/10.1139/v08-018.

Lin, Wang-Hong, Jim-Min Fang, and Yu-Shia Cheng. 1996. “Diterpenes and Related Cycloadducts from Taiwania Cryptomerioides.” *Phytochemistry* 42 (6): 1657–63.

Ling, Tie-Jun, Wei-Wei Ling, Yuan-Jun Chen, Xiao-Chun Wan, Tao Xia, Xian-Feng Du, and Zheng-Zhu Zhang. 2010. “Antiseptic Activity and Phenolic Constituents of the Aerial Parts of Vitex Negundo Var. Cannabifolia.” *Molecules* 15 (11): 8469–77.

Lu, Yinron g, and L Yeap Foo. 2000. “Flavonoid and Phenolic Glycosides from Salvia Officinalis.” *Phytochemistry* 55 (3): 263–67.

Lu, Yinrong, and L Yeap Foo. 2002. “Polyphenolics of Salvia—a Review.” *Phytochemistry* 59 (2): 117–40.

Luecha, Prathan, Kaoru Umehara, Toshio Miyase, and Hiroshi Noguchi. 2009. “Antiestrogenic Constituents of the Thai Medicinal Plants Capparis Flavicans and Vitex Glabrata.” *Journal of Natural Products* 72 (11): 1954–59.

Marco, J Alberto, Juan F Sanz-Cervera, Vicente Garcia-Lliso, Miguel Guara, and Joan Vallès-Xirau. 1997. “Sesquiterpene Lactones from Artemisia Inculta.” *Phytochemistry* 45 (4): 751–54.

Maruyama, Masao, Kumi Watanabe, Tadahiro Kawakami, Masao Maeda, Mitsuhiro Kato, Shigeo Nozoe, and Tomihisa Ohta. 1995. “Ineupatorolides from Carpesium Glossophyllum.” *Planta Medica* 61 (04): 388–89.

Matsunami, Katsuyoshi, Jiro Nagashima, Sachiko Sugimoto, Hideaki Otsuka, Yoshio Takeda, Duangporn Lhieochaiphant, and Sorasak Lhieochaiphant. 2010. “Megastigmane Glucosides and an Unusual Monoterpene from the Leaves of Cananga Odorata Var. Odorata, and Absolute Structures of Megastigmane Glucosides Isolated from C. Odorata Var. Odorata and Breynia Officinalis.” *Journal of Natural Medicines* 64: 460–67.

McKee, Tawnya C, Conni D Covington, Richard W Fuller, Heidi R Bokesch, Sherry Young, John H Cardellina, Marian R Kadushin, D Doel Soejarto, Peter F Stevens, and Gordon M Cragg. 1998. “Pyranocoumarins from Tropical Species of the Genus Calophyllum: A Chemotaxonomic Study of Extracts in the National Cancer Institute Collection.” *Journal of Natural Products* 61 (10): 1252–56.

Mendes, Eduarda, José L Marco, Benjamin Rodríguez, María L Jimeno, Ana M Lobo, and Sundaresan Prabhakar. 1989. “Diterpenoids from Salvia Candelabrum.” *Phytochemistry* 28 (6): 1685–90.

Meng, Dahai, Jian Wu, and Weimin Zhao. 2010. “Glycosides from Breynia Fruticosa and Breynia Rostrata.” *Phytochemistry* 71 (2–3): 325–31.

Miron-Lopez, Gumersindo, Isabel L Bazzocchi, Ignacio A Jimenez-Diaz, Laila M Moujir, Ramiro Quijano-Quiñones, Leovigildo Quijano, and Gonzalo J Mena-Rejon. 2014. “Cytotoxic Diterpenes from Roots of Crossopetalum Gaumeri, a Celastraceae Species from Yucatan Peninsula.” *Bioorganic & Medicinal Chemistry Letters* 24 (9): 2105–9.

Miyaichi, Yukinori, Akiko Segawa, and Tsuyoshi Tomimori. 2006. “Studies on Nepalese Crude Drugs. XXIX. Chemical Constituents of Dronapuspi, the Whole Herb of Leucas Cephalotes S PRENG.” *Chemical and Pharmaceutical Bulletin* 54 (10): 1370–79.

Miyase, Toshio, and Akira Ueno. 1991. “Ionone and Bibenzyl Glycosides from Epimedium Grandiflorum Var. Thunbergianum.” *Phytochemistry* 30 (5): 1727–28.

Moghaddam, Firouz Matloubi, Mahdi Moridi Farimani, Marjan Seirafi, Salman Taheri, Hamid Reza Khavasi, Jandirk Sendker, Peter Proksch, Victor Wray, and RuAngelie Edrada. 2010. “Sesterterpenoids and Other Constituents of Salvia Sahendica.” *Journal of Natural Products* 73 (9): 1601–5.

Mooi, Lim Yang, Norhanom Abdul Wahab, Nordin Haji Lajis, and Abdul Manaf Ali. 2010. “Chemopreventive Properties of Phytosterols and Maslinic Acid Extracted from Coleus Tuberosus in Inhibiting the Expression of EBV Early-Antigen in Raji Cells.” *Chemistry & Biodiversity* 7 (5): 1267–75. https://doi.org/https://doi.org/10.1002/cbdv.200900193.

Murata, Toshihiro, Toshio Miyase, and Fumihiko Yoshizaki. 2010. “Cyclic Spermidine Alkaloids and Flavone Glycosides from Meehania Fargesii.” *Chemical and Pharmaceutical Bulletin* 58 (5): 696–702.

Murata, Toshihiro, Kenroh Sasaki, Kumiko Sato, Fumihiko Yoshizaki, Haruna Yamada, Hiromichi Mutoh, Kaoru Umehara, Toshio Miyase, Tsutomu Warashina, and Hiroaki Aoshima. 2009. “Matrix Metalloproteinase-2 Inhibitors from Clinopodium Chinense Var. Parviflorum.” *Journal of Natural Products* 72 (8): 1379–84.

Murata, Toshihiro, Mai Watahiki, Yu Tanaka, Toshio Miyase, and Fumihiko Yoshizaki. 2010. “Hyaluronidase Inhibitors from Takuran, Lycopus Lucidus.” *Chemical and Pharmaceutical Bulletin* 58 (3): 394–97.

Nabih, Heba K, Rümeysa Yücer, Nuha Mahmoud, Mona Dawood, Mohamed Elbadawi, Nasim Shahhamzehei, Mohamed A M Atia, Ahmed AbdelSadik, Taha A Hussien, and Mahmoud A A Ibrahim. 2024. “The Cytotoxic Activities of the Major Diterpene Extracted from Salvia Multicaulis (Bardakosh) Are Mediated by the Regulation of Heat-Shock Response and Fatty Acid Metabolism Pathways in Human Leukemia Cells.” *Phytomedicine* 135: 156023.

Nagashima, Fumihiro, Katsuhiro Kishi, Yuko Hamada, Shigeru Takaoka, and Yoshinori Asakawa. 2005. “Ent-Verticillane-Type Diterpenoids from the Japanese Liverwort Jackiella Javanica.” *Phytochemistry* 66 (14): 1662–70.

Nakagawa, Hiroyuki, Yoshihisa Takaishi, Naonobu Tanaka, Koichiro Tsuchiya, Hirofumi Shibata, and Tomihiko Higuti. 2006. “Chemical Constituents from the Peels of Citrus s Udachi.” *Journal of Natural Products* 69 (8): 1177–79.

Nishitoba, Tsuyoshi, Sanae Goto, Hiroji Sato, and Sadao Sakamura. 1989. “Bitter Triterpenoids from the Fungus Ganoderma Applanatum.” *Phytochemistry* 28 (1): 193–97.

NISIBE, Sansei, Hidehiro Kinoshita, Hidekatsu Takeda, and Goroh OKANO. 1990. “Phenolic Compounds from Stem Bark of Acanthopanax Senticosus and Their Pharmacological Effect in Chronic Swimming Stressed Rats.” *Chemical and Pharmaceutical Bulletin* 38 (6): 1763–65.

Oketch-Rabah, H A, E Lemmich, S F Dossaji, Thor G Theander, Carl E Olsen, Claus Cornett, Arsalan Kharazmi, and S Brøgger Christensen. 1997. “Two New Antiprotozoal 5-Methylcoumarins from Vernonia Brachycalyx.” *Journal of Natural Products* 60 (5): 458–61.

Oramas-Royo, Sandra M, Haydee Chávez, Patricia Martin-Rodriguez, Leandro Fernández-Pérez, Angel G Ravelo, and Ana Estévez-Braun. 2010. “Cytotoxic Triterpenoids from Maytenus Retusa.” *Journal of Natural Products* 73 (12): 2029–34.

OTSUKA, Hideaki, Masami YAO, Kenji KAMADA, and Yoshio TAKEDA. 1995. “Alangionosides G-M : Glycosides of Megastigmane Derivatives from the Leaves of Alangium Premnifolium.” *CHEMICAL & PHARMACEUTICAL BULLETIN* 43 (5): 754–59. https://doi.org/10.1248/cpb.43.754.

Passreiter, Claus M, Jesus Sandoval-Ramirez, and Colin W Wright. 1999. “Sesquiterpene Lactones from Neurolaena o Axacana.” *Journal of Natural Products* 62 (8): 1093–95.

Pereda-Miranda, Rogelio, and Guillermo Delgado. 1986. “Flavonoids from Salvia Nicolsoniana.” *Journal of Natural Products* 49 (6): 1160–61.

Piccinelli, Anna Lisa, Swizly Arana, Armando Caceres, Roberta d’Emmanuele di Villa Bianca, Raffaella Sorrentino, and Luca Rastrelli. 2004. “New Lignans from the Roots of Valeriana p Rionophylla with Antioxidative and Vasorelaxant Activities.” *Journal of Natural Products* 67 (7): 1135–40.

Polonsky, Judith, Jacqueline Gallas, Jeannette Varenne, Thierry Prangé, Claudine Pascard, Henri Jacquemin, and Christian Moretti. 1982. “Isolation and Structure (x-Ray Analysis) of Karinolide, a New Quassinoid from Simaba Multiflora.” *Tetrahedron Letters* 23 (8): 869–72.

Ragasa, Consolacion Y, Joy G Hofileña, and John A Rideout. 2002. “New Furanoid Diterpenes from Caesalpinia p Ulcherrima.” *Journal of Natural Products* 65 (8): 1107–10.

Randriamampionona, Denis, Billo Diallo, Francisco Rakotoniriana, Christian Rabemanantsoa, Kiban Cheuk, Anne-Marie Corbisier, Jacques Mahillon, Suzanne Ratsimamanga, and Mondher El Jaziri. 2007. “Comparative Analysis of Active Constituents in Centella Asiatica Samples from Madagascar: Application for Ex Situ Conservation and Clonal Propagation.” *Fitoterapia* 78 (7–8): 482–89.

Regos, Ionela, Andrea Urbanella, and Dieter Treutter. 2009. “Identification and Quantification of Phenolic Compounds from the Forage Legume Sainfoin (Onobrychis Viciifolia).” *Journal of Agricultural and Food Chemistry* 57 (13): 5843–52.

Rumalla, Chidananda S, Zulfiqar Ali, Aruna D Weerasooriya, Troy J Smillie, and Ikhlas A Khan. 2010. “A New Triterpene Glycoside from Centella Erecta.” *Fitoterapia* 81 (7): 751–54.

Sadyrbekov, D T, G A Atazhanova, A T Kulyyasov, V A Raldugin, Yu V Gatilov, M M Shakirov, T T Edil’baeva, K M Turdybekov, and S M Adekenov. 2006. “Buddledin C from Pulicaria Prostrata and Selective Synthesis of Its Epoxy Derivative.” *Chemistry of Natural Compounds* 42: 41–45.

Şahin, F Pınar, Nurten Ezer, and İhsan Çalış. 2004. “Three Acylated Flavone Glycosides from Sideritis Ozturkii Aytac & Aksoy.” *Phytochemistry* 65 (14): 2095–99.

Sandjo, Louis Pergaud, Abdou Tchoukoua, Hippolyte Nga Ntede, Mehdi Yemloul, Enrico Perspicace, Felix Keumedjio, François Couty, Gilbert Kirsch, and Bonaventure Tchaleu Ngadjui. 2010. “New Nortriterpenoid and Ceramides from Stems and Leaves of Cultivated Triumfetta Cordifolia A Rich (Tiliaceae).” *Journal of the American Oil Chemists’ Society* 87: 1167–77.

Sanduja, R, G E Martin, A J Weinheimer, M Alam, M B Hossain, and Dick van der Helm. 1984. “Secondary Metabolites of the Coelenterate Echinopora Lamellosa.” *Journal of Heterocyclic Chemistry* 21 (3): 845–48.

Schmeda-Hirschmann, G, R Boeker, J Jakupovic, and F Bohlmann. 1986. “A Myrtenylfuroheliangolide from Calea Rupicola.” *Phytochemistry* 25 (7): 1753–54.

Shakirullah, Mohammad, Hanif Ahmad, Muhammad Raza Shah, Imtiaz Ahmad, Muhammad Ishaq, Nematullah Khan, Amir Badshah, and Inamullah Khan. 2011. “Antimicrobial Activities of Conyzolide and Conyzoflavone from Conyza Canadensis.” *Journal of Enzyme Inhibition and Medicinal Chemistry* 26 (4): 468–71.

Shen, Ya-Ching, Cheng Lin Lee, Ashraf Taha Khalil, Yuan-Bin Cheng, Ching-Te Chien, and Yao-Haur Kuo. 2005. “New Clerodane Diterpenoids from Casearia Membranacea.” *Helvetica Chimica Acta* 88 (1): 68–77. https://doi.org/https://doi.org/10.1002/hlca.200490297.

Shirota, Osamu, Jennifer M Oribello, Setsuko Sekita, and Motoyoshi Satake. 2011. “Sesquiterpenes from Blumea Balsamifera.” *Journal of Natural Products* 74 (3): 470–76. https://doi.org/10.1021/np100646n.

Shukla, Vishnu S, Subhash C Dutta, Robindra N Baruah, Ram P Sharma, Gopalakrishna Thyagarajan, Werner Herz, Narendra Kumar, Kinzo Watanabe, and John F Blount. 1982. “New 5-Methylcoumarins from Ethulia Conyzoides.” *Phytochemistry* 21 (7): 1725–31.

Si, Chuan-Ling, Shu-Ming Li, Zhong Liu, Jin-Kyu Kim, and Young-Soo Bae. 2011. “Antioxidant Phenolic Glycosides from the Bark of Populus Ussuriensis Kom.” *Natural Product Research* 25 (15): 1396–1401.

Su, Chung-Ren, Yuh-Fung Chen, Meei-Jen Liou, Huei-Yann Tsai, Wen-Shin Chang, and Tian-Shung Wu. 2008. “Anti-Inflammatory Activities of Furanoditerpenoids and Other Constituents from Fibraurea Tinctoria.” *Bioorganic & Medicinal Chemistry* 16 (21): 9603–9.

Suzuki, Kyoko, Mamoru Okasaka, Yoshiki Kashiwada, Yoshihisa Takaishi, Gisho Honda, Michiho Ito, Yoshio Takeda, et al. 2007. “Sesquiterpene Lactones from the Roots of Ferula Varia and Their Cytotoxic Activity.” *Journal of Natural Products* 70 (12): 1915–18. https://doi.org/10.1021/np0703996.

Syamasundar, K V, Gopal R Mallavarapu, and E Murali Krishna. 1991. “Triterpenoids of the Resin of Bursera Delpechiana.” *Phytochemistry* 30 (1): 362–63.

Takashima, Junko, and Ayumi Ohsaki. 2002. “Brosimacutins A− I, Nine New Flavonoids from Brosimum a Cutifolium.” *Journal of Natural Products* 65 (12): 1843–47.

Takenaka, Makoto, Toshiro Watanabe, Kazuo Sugahara, Yasuo Harada, Shigeo Yoshida, and Fumio Sugawara. 1997. “New Antimicrobial Substances against Streptomyces Scabies from Rosemary (Rosmarinus Officinalis L.).” *Bioscience, Biotechnology, and Biochemistry* 61 (9): 1440–44.

Takeya, Koichi, Hideyuki Kobata, Akira Ozeki, Hiroshi Morita, and Hideji Itokawa. 1998. “Quassinoids from Ailanthus Vilmoriniana.” *Phytochemistry* 48 (3): 565–68.

Tezuka, Yasuhiro, Rena Kasimu, Jian Xin Li, Purusotam BAsNET, Ken TANAKA, Tsuneo NAMBA, and Shigetoshi KADOTA. 1998. “Constituents of Roots of Salvia Deserta SCHANG.(Xinjiang-Danshen).” *Chemical and Pharmaceutical Bulletin* 46 (1): 107–12.

Tian-Xiu, Qian, and Li Lian-Niang. 1992. “Isosalvianolic Acid C, a Depside Possessing a Dibenzooxepin Skeleton.” *Phytochemistry* 31 (3): 1068–70.

Tong, Xuhui, Shigang Lin, Makoto Fujii, and De-Xing Hou. 2004. “Molecular Mechanisms of Echinocystic Acid-Induced Apoptosis in HepG2 Cells.” *Biochemical and Biophysical Research Communications* 321 (3): 539–46. https://doi.org/https://doi.org/10.1016/j.bbrc.2004.07.004.

Topcu, GÜLAÇTI, Nur Tan, A Ulubelen, D Sun, and W H Watson. 1995. “Terpenoids and Flavonoids from the Aerial Parts of Salvia Candidissima.” *Phytochemistry* 40 (2): 501–4.

Topcu, Gülaçti, Ayhan Ulubelen, Timothy C-M Tam, and Chun Tao-Che. 1996. “Sesterterpenes and Other Constituents of Salvia Yosgadensis.” *Phytochemistry* 42 (4): 1089–92.

Tori, Motoo, Kaori Honda, Hiromi Nakamizo, Yasuko Okamoto, Misato Sakaoku, Shigeru Takaoka, Xun Gong, Yuemao Shen, Chiaki Kuroda, and Ryo Hanai. 2006. “Chemical Constituents of Ligularia Virgaurea and Its Diversity in Southwestern Sichuan of China.” *Tetrahedron* 62 (20): 4988–95.

Traoré, Maminata, Jerzy W Jaroszewski, Carl Erik Olsen, Jean Bosco Ouédraogo, Guissou I Pierre, Odile G Nacoulma, T Robert Guiguemdé, and S Brøgger Christensen. 2008. “A New Oxygenated Ursane Derivative from Canthium Multiflorum.” *Planta Medica* 74 (05): 560–62.

Tsai, Ian-Lih, Chih-Feng Hsieh, and Chang-Yih Duh. 1998. “Additional Cytotoxic Neolignans from Persea Obovatifolia.” *Phytochemistry* 48 (8): 1371–75.

Tsai, Wei-Jern, Chien-Chang Shen, Tung-Hu Tsai, and Lie-Chwen Lin. 2014. “Lignans from the Aerial Parts of Saururus Chinensis: Isolation, Structural Characterization, and Their Effects on Platelet Aggregation.” *Journal of Natural Products* 77 (1): 125–31.

Tsichritzis, F, and J Jakupovic. 1990. “Diterpenes and Other Constituents FromRelhania Species.” *Phytochemistry* 29 (10): 3173–87.

Tsukamoto, Hiroki, Sueo Hisada, and SANSEI NISHIBE. 1985. “Lignans from Bark of the Olea Plants. II.” *Chemical and Pharmaceutical Bulletin* 33 (3): 1232–41.

Ulubelen, A, M Miski, and T J Mabry. 1981. “Further Flavones and Triterpenes and the New 6-Hydroxyluteolin 5-β-D-Glucoside from Salvia Tomentosa.” *Journal of Natural Products* 44 (5): 586–87.

Ulubelen, Ayhan, Sevil Öksüz, Ufuk Kolak, Hüsniye Birman, and Wolfgang Voelter. 2000. “Cardioactive Terpenoids and a New Rearranged Diterpene from Salvia Syriaca.” *Planta Medica* 66 (07): 627–29.

Ulubelen, Ayhan, Nur Tan, Ufuk Sönmez, and Gülaçti Topcu. 1998. “Diterpenoids and Triterpenoids from Salvia Multicaulis.” *Phytochemistry* 47 (5): 899–901. https://doi.org/https://doi.org/10.1016/S0031-9422(97)00540-2.

Wada, Shun-ich, and Reiko Tanaka. 2000. “Four New Trisnorlanostene-Type Triterpenoids from the Stem Bark of Pinus Luchuensis.” *Journal of Natural Products* 63 (8): 1055–57. https://doi.org/10.1021/np000155k.

Wang, Jia, Hui Yang, Zhong-Wen Lin, and Han-Dong Sun. 1997. “Flavonoids from Bidens Pilosa Var. Radiata.” *Phytochemistry* 46 (7): 1275–78.

Wang, Mingfu, Jiangang Li, Meera Rangarajan, Yu Shao, Edmond J LaVoie, Tzou-Chi Huang, and Chi-Tang Ho. 1998. “Antioxidative Phenolic Compounds from Sage (Salvia Officinalis).” *Journal of Agricultural and Food Chemistry* 46 (12): 4869–73.

Wang, Xian‐You, Dong Wang, Xiao‐Xia Ma, Ying‐Jun Zhang, and Chong‐Ren Yang. 2008. “Two New Dammarane‐type Bisdesmosides from the Fruit Pedicels of Panax Notoginseng.” *Helvetica Chimica Acta* 91 (1): 60–66.

Wilzer, Karla A, Frank R Fronczek, Lowell E Urbatsch, and Nikolaus H Fischer. 1989. “Coumarins from Aster Praealtus.” *Phytochemistry* 28 (6): 1729–35. https://doi.org/https://doi.org/10.1016/S0031-9422(00)97834-8.

Wollenweber, Eckhard, Marion Dörr, Abdolhossein Rustaiyan, James N Roitman, and Earl H Graven. 1992. “Exudate Flavonoids of Some Salvia and a Trichostema Species.” *Zeitschrift Für Naturforschung C* 47 (9–10): 782–84.

Wongsa, Nikhom, Somdej Kanokmedhakul, and Kwanjai Kanokmedhakul. 2011. “Cananginones A–I, Linear Acetogenins from the Stem Bark of Cananga Latifolia.” *Phytochemistry* 72 (14): 1859–64. https://doi.org/https://doi.org/10.1016/j.phytochem.2011.05.013.

Wu, Jian, and Wei-Min Zhao. 2010. “New Sesquiterpene and Triterpene from the Fruits of Cryptomeria Fortunei.” *Journal of Asian Natural Products Research* 12 (5): 382–87.

Wu, Jianming, Jian Li, Zhiyong Zhu, Jiang Li, Guojun Huang, Yao Tang, and Xiaoping Gao. 2010. “Protective Effects of Echinocystic Acid Isolated from Gleditsia Sinensis Lam. against Acute Myocardial Ischemia.” *Fitoterapia* 81 (1): 8–10.

Wu, Tian-Shung, Yann-Lii Leu, Yu-Yi Chan, Sheu-Meei Yu, Che-Ming Teng, and Jeng-De Su. 1994. “Lignans and an Aromatic Acid from Cinnamomum Philippinense.” *Phytochemistry* 36 (3): 785–88.

Xie, Yuanyuan, Toshio Morikawa, Kiyofumi Ninomiya, Katsuya Imura, Osamu Muraoka, Dan Yuan, and Masayuki Yoshikawa. 2008. “Medicinal Flowers. XXIII. New Taraxastane-Type Triterpene, Punicanolic Acid, with Tumor Necrosis Factor-α Inhibitory Activity from the Flowers of Punica Granatum.” *Chemical and Pharmaceutical Bulletin* 56 (11): 1628–31.

Xu, Fengming, Toshio Morikawa, Hisashi Matsuda, Kiyofumi Ninomiya, and Masayuki Yoshikawa. 2004. “Structures of New Sesquiterpenes and Hepatoprotective Constituents from the Egyptian Herbal Medicine Cyperus Longus.” *Journal of Natural Products* 67 (4): 569–76. https://doi.org/10.1021/np030368k.

Xu, Junju, Changjiu Ji, Yumei Zhang, Jia Su, Yan Li, and Ninghua Tan. 2012. “Inhibitory Activity of Eudesmane Sesquiterpenes from Alpinia Oxyphylla on Production of Nitric Oxide.” *Bioorganic & Medicinal Chemistry Letters* 22 (4): 1660–63.

Xu, Mingfeng, Lianqing Shen, Kuiwu Wang, and Qizhen Du. 2011. “Two New Abietane Diterpenoids from the Stems of Clerodendrum Kaichianum PS Hsu.” *Helvetica Chimica Acta* 94 (3): 539–44.

Yamamoto, Atsushi, Shigehiko Nitta, Toshio Miyase, Akira Ueno, and Wu Li-Jun. 1993. “Phenylethanoid and Lignan-Iridoid Complex Glycosides from Roots of Buddleja Davidii.” *Phytochemistry* 32 (2): 421–25. https://doi.org/https://doi.org/10.1016/S0031-9422(00)95007-6.

Yang, Heejung, Hyun-Jong Cho, So Hee Sim, Young Keun Chung, Dae-Duk Kim, Sang Hyun Sung, Jinwoong Kim, and Young Choong Kim. 2012. “Cytotoxic Terpenoids from Juglans Sinensis Leaves and Twigs.” *Bioorganic & Medicinal Chemistry Letters* 22 (5): 2079–83.

Yang, Yu‐Liang, Fang‐Rong Chang, and Yang‐Chang Wu. 2005. “Squadinorlignoside: A Novel 7, 9′‐Dinorlignan from the Stems of Annona Squamosa.” *Helvetica Chimica Acta* 88 (10): 2731–37.

Ying, Bai-ping, Isao Kubo, Takeshi Matsumoto, and Yuji Hayashi. 1990. “Congeners of Norditerpene Dilactones from Podocarpus Nagi.” *Phytochemistry* 29 (12): 3953–55.

Yoshikawa, Masayuki, Seikou Nakamura, Xuezheng Li, and Hisashi Matsuda. 2008. “Reinvestigation of Absolute Stereostructure of (−)-Rosiridol: Structures of Monoterpene Glycosides, Rosiridin, Rosiridosides A, B, and C, from Rhodiola Sachalinensis.” *Chemical and Pharmaceutical Bulletin* 56 (5): 695–700.

Yousuf, Maryam H Al, Ahmed K Bashir, Gerald Blunden, Trevor A Crabb, and Asmita V Patel. 2002. “6-Methylcryptoacetalide, 6-Methyl-Epicryptoacetalide and 6-Methylcryptotanshinone from Salvia Aegyptiaca.” *Phytochemistry* 61 (4): 361–65. https://doi.org/https://doi.org/10.1016/S0031-9422(02)00283-2.

Zdero, C, F Bohlmann, and R M King. 1992. “Clerodane Derivatives FromDiplostephium.” *Phytochemistry* 31 (1): 213–16. https://doi.org/https://doi.org/10.1016/0031-9422(91)83038-M.

Zhang, Zhizhen, Dean Guo, Changling Li, Junhua Zheng, Kazuo Koike, Zhonghua Jia, and Tamotsu Nikaido. 1999. “Two Diterpenoids from the Roots of Gaultheria Yunnanensis.” *Journal of Natural Products* 62 (2): 297–98.

Zhao, Jianglin, Jingfeng Lou, Yan Mou, Peiqin Li, Jianyong Wu, and Ligang Zhou. 2011. “Diterpenoid Tanshinones and Phenolic Acids from Cultured Hairy Roots of Salvia Miltiorrhiza Bunge and Their Antimicrobial Activities.” *Molecules* 16 (3): 2259–67.

Zhao, Li-Min, Xiao-Tian Liang, and Lian-Niang Li. 1996. “Prionitisides A and B, Two Phenolic Glycosides from Salvia Prionitis.” *Phytochemistry* 42 (3): 899–901.

Zheng, Cheng-Jian, Bao-Kang Huang, Yang Wang, Qi Ye, Ting Han, Qiao-Yan Zhang, Hong Zhang, and Lu-Ping Qin. 2010. “Anti-Inflammatory Diterpenes from the Seeds of Vitex Negundo.” *Bioorganic & Medicinal Chemistry* 18 (1): 175–81.

Zheng, Wei, and Shiow Y Wang. 2001. “Antioxidant Activity and Phenolic Compounds in Selected Herbs.” *Journal of Agricultural and Food Chemistry* 49 (11): 5165–70.

A
